# Regulatory mechanisms of PD-1/PD-L1 in cancers

**DOI:** 10.1186/s12943-024-02023-w

**Published:** 2024-05-18

**Authors:** Xin Lin, Kuan Kang, Pan Chen, Zhaoyang Zeng, Guiyuan Li, Wei Xiong, Mei Yi, Bo Xiang

**Affiliations:** 1 NHC Key Laboratory of Carcinogenesis and Hunan Key Laboratory of Cancer Metabolism, Hunan Cancer Hospital and the Affiliated Cancer Hospital of Xiangya School of Medicine, Central South University, Changsha, 410013 Hunan China; 2 FuRong Laboratory, Changsha, 410078 Hunan China; 3 Cancer Research Institute, School of Basic Medical Sciences, Central South University, Changsha, 410008 Hunan China; 4 The Key Laboratory of Carcinogenesis and Cancer Invasion of the Chinese Ministry of Education, Central South University, Changsha, 410078 Hunan China; 5 Department of Dermotology, National Clinical Research Center for Geriatric Disorders, Xiangya Hospital, Central South University, Changsha, 410008 Hunan China; 6 Hunan Cancer Hospital and the Affiliated Cancer Hospital of Xiangya School of Medicine, Central South University, Tongzipo Road, Changsha, 410013 Hunan China

**Keywords:** Tumor immunity, PD-1, PD-L1, Regulatory mechanism, Combination therapy

## Abstract

Immune evasion contributes to cancer growth and progression. Cancer cells have the ability to activate different immune checkpoint pathways that harbor immunosuppressive functions. The programmed death protein 1 (PD-1) and programmed cell death ligands (PD-Ls) are considered to be the major immune checkpoint molecules. The interaction of PD-1 and PD-L1 negatively regulates adaptive immune response mainly by inhibiting the activity of effector T cells while enhancing the function of immunosuppressive regulatory T cells (Tregs), largely contributing to the maintenance of immune homeostasis that prevents dysregulated immunity and harmful immune responses. However, cancer cells exploit the PD-1/PD-L1 axis to cause immune escape in cancer development and progression. Blockade of PD-1/PD-L1 by neutralizing antibodies restores T cells activity and enhances anti-tumor immunity, achieving remarkable success in cancer therapy. Therefore, the regulatory mechanisms of PD-1/PD-L1 in cancers have attracted an increasing attention. This article aims to provide a comprehensive review of the roles of the PD-1/PD-L1 signaling in human autoimmune diseases and cancers. We summarize all aspects of regulatory mechanisms underlying the expression and activity of PD-1 and PD-L1 in cancers, including genetic, epigenetic, post-transcriptional and post-translational regulatory mechanisms. In addition, we further summarize the progress in clinical research on the antitumor effects of targeting PD-1/PD-L1 antibodies alone and in combination with other therapeutic approaches, providing new strategies for finding new tumor markers and developing combined therapeutic approaches.

## Introduction

Immune suppression largely contributes to cancer occurrence and progression. The programmed cell death protein 1 (PD-1, also known as PDCD1 and CD279) was originally identified by Ishida et al. in apoptotic mouse T-cell tumors [1]. PD-1 is a transmembrane protein belonging to the CD28/CTLA-4 superfamily. It is widely expressed at the surface of activated T cells, B cells, monocytes, and other immune cells, and negatively regulates human immune response through binding with its two ligands, namely programmed cell death 1 ligands (PD-L1 or PD-L2). PD-L1 (B7-H1; CD274) and PD-L2 (B7-DC; CD273) belong to the B7 family of T cell co-inhibitory molecules. PD-L1 is widely expressed in antigen-presenting cells and tissues, such as heart and lung [2, 3]. The interaction of PD-1 with PD-L1 or PD-L2 provides inhibitory signals responsible for inhibiting T cell signaling, mediating the mechanisms of tolerance, and providing immune homeostasis. Therefore, PD-1 suppresses autoimmunity and prevents the occurrence of autoimmune diseases. In addition, PD-L1 or PD-L2 expressed by cancer cells binds to PD-1 on the surface of T cells, thereby inhibiting T cell activation and leading to cancer immune escape [4]. Numerous studies revealed that PD-L1 expression is very high in lung cancer, melanoma, glioma, breast cancer and other malignant tumor cells, forming an immunosuppressive tumor microenvironment [5].

PD-1 mainly consists of extracellular IgV-like domain region, hydrophobic transmembrane region and cytoplasmic region, and the tail of the cytoplasmic region has immunoreceptor tyrosine-based inhibitory motif (ITIM) and immunoreceptor tyrosine-based switch motif (ITSM) [6, 7], which is an important structural basis for PD-1 to transmit inhibitory signals and perform immunosuppressive functions. PD-L1 is structurally similar to PD-1 and is more conserved and widely expressed than PD-L2 [8], so it plays the leading effect in tumor cells immune evasion. In recent years, antagonistic antibodies against PD-1 or PD-L1 have been approved by the FDA to treat cancer, opening a new chapter in tumor immunotherapy across the era [9].

Anti-PD-1/PD-L1 inhibitors have become effective immune checkpoint inhibitors (ICIs) and are rapidly becoming the standard therapy for various cancers. Tumor immunotherapy aims to block the activity of inhibitory immune checkpoint proteins and promote T cell activation to achieve anti-tumor immune effects [10]. Owing to their safety and precision, these inhibitors hold significant promise in tumor immunotherapy. Research indicates that the PD-1/PD-L1 pathway plays a crucial role in regulating autoimmunity responses and peripheral tolerance. Notably, anti-PD-1/PD-L1 immunotherapy can effectively block the PD-1/PD-L1 signaling pathway, restore T cell activity, enhance anti-tumor immunity, and then eliminate tumor cells [11, 12]. Therefore, the discovery of multiple immunotherapies, such as PD-1 and PD-L1 inhibitors, has significant clinical implications for tumor-specific immunotherapy. This paper aims to provide a relatively comprehensive review of the mechanisms of PD-1/PD-L1 signaling in autoimmunity and tumor immunity, as well as its clinical efficacy in different tumors. It serves as a reference for exploring the internal regulatory mechanisms and resistance mechanisms of cancer immunotherapy targeting PD-1/PD-L1. We aim to offer new strategies for identifying novel tumor markers and developing combined drug treatments, thereby achieving early diagnosis and personalized treatment for patients.

### The physiological function and immune regulation mechanism of the PD-1/PD-L1 axis as an immune checkpoint

Under normal physiological conditions, PD-1 and PD-L1 molecules are among the primary participants in maintaining immune homeostasis. Their binding reduces autoimmune cell attacking on self-tissues, maintains immune balance by inhibiting the activation of T cells. T cell activation primarily relies on a “dual-signal” system. Antigen-presenting cells (APCs) capture and process bacterial antigens, subsequently presenting them to the T-cell receptor (TCR) through the major histocompatibility complex (MHC) molecule. Upon recognition and binding to form the TCR-MHC complex, a series of co-stimulatory signals are required to further induce the immune response of effector T cells [13, 14]. Co-stimulatory molecules expressed on APCs, such as CD80 and CD86, bind to CD28 on T-cells to provide co-stimulatory signals, thoroughly activating T-cells to specifically target and kill cells infected by pathogens. Negative co-stimulatory molecules like CTLA4 and PD-1/PD-L1 play an essential role in preventing tissue inflammation and autoimmune diseases under normal physiological conditions by avoiding excessive activation of T cells. When cells become cancerous, they will exploit this inhibitory pathway to escape the siege of the immune system [15, 16]. Furthermore, the interaction between TCR and MHC is highly specific and sensitive, enabling T cells to detect rare antigenic epitopes on APCs. Due to central and peripheral immune tolerance, autoreactive T cells that recognize specific antigens are usually in an unresponsive state [17]. This might explain why reactive T cells detected in patients typically fail to control or eradicate advanced diseases.

**Fig. 1:**
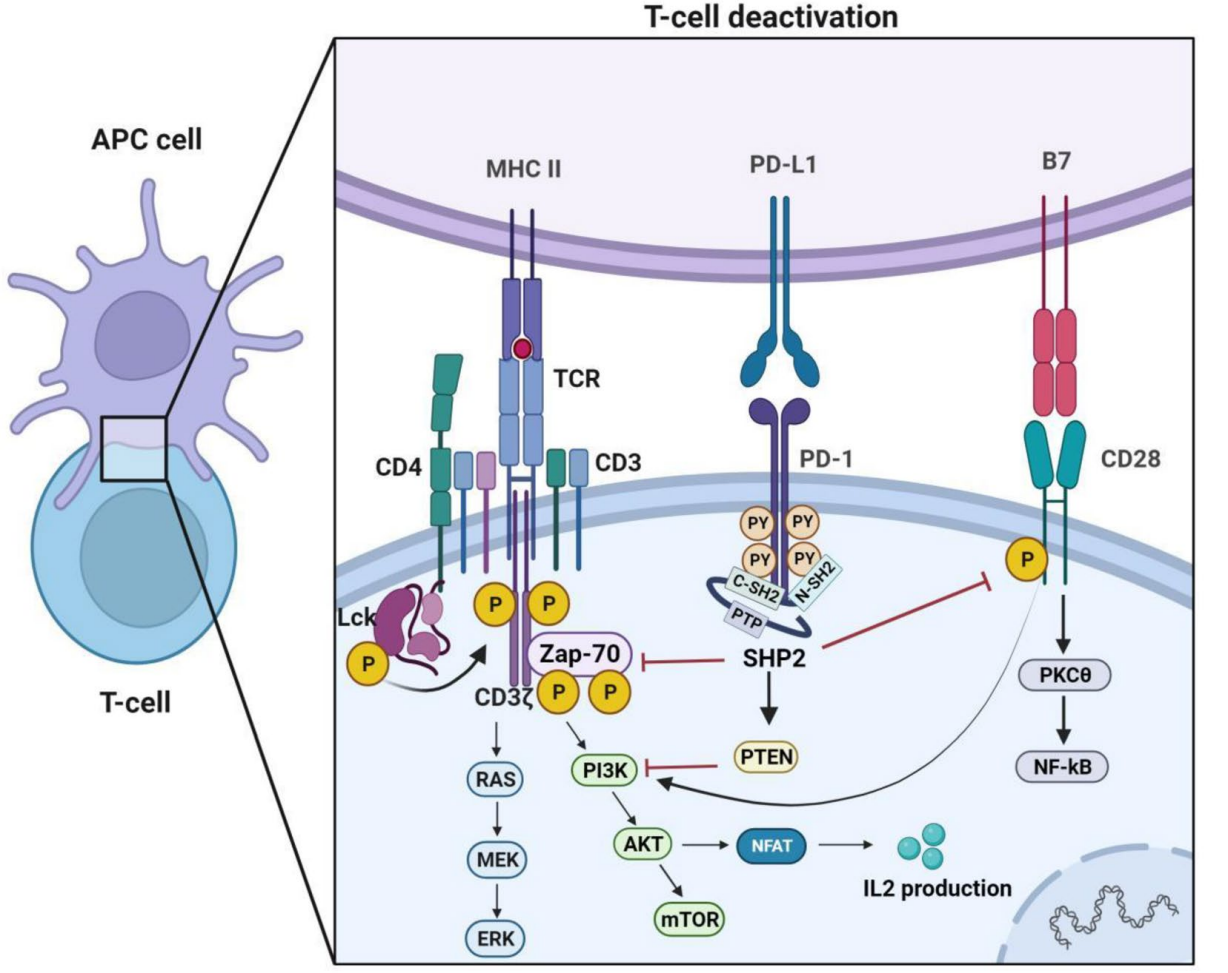
The immune regulation mechanism of the PD-1/PD-L1 axis. Antigen-presenting cells (APCs) deliver tumor antigens to the T-cell receptor (TCR) via the major histocompatibility complex(MHC), and when the MHC-antigen complex specifically binds to the TCR, it triggers a series of signal transductions, including the phosphatidylinositol signaling and mitogen- activated protein kinases signaling pathways, thus activating the immune responses of effector T cells. Upon PD-L1 binding to PD-1, phosphorylation of tyrosine residues in the ITSM and ITIM domains of the PD-1 cytoplasmic region occurs, recruiting and activating SHP2. Subsequently, recruited SHP-2 mediates dephosphorylation of TCR-associated CD3 and ZAP70 signalosomes, while inhibiting CD28 co-stimulatory signals. This further attenuates downstream TCR signaling strength and cytokine secretion, such as IL-2, ultimately inhibiting the function of T cells. Figure created with BioRender

In the human immune system, the binding of PD-L1 to PD-1 primarily exerts an immunosuppressive regulatory effect through Src homology region 2 domain-containing phosphatase-2 (SHP2), thereby attenuating the immune response of T cells [18]. SHP2 is a tyrosine phosphatase composed of N-SH2, the C-SH2 structural domain, the PTP structural domain, and the C-terminal tail, among which N-SH2 plays a core function in the activation process of SHP2. In the inactive state, an intramolecular interaction occurs between the N-SH2 and PTP domains of SHP2, effectively obstructing the entry of SHP2 substrates into the catalytic site. The activation of SHP2 requires the removal of the PTP domain from the N-SH2 domain while concurrently binding to a specific phosphotyrosine motif, thereby destroying its self-inhibition state and exerting its dephosphorylation function [19]. When PD-1 binds to PD-L1, it can induce phosphorylation of tyrosine residues in the ITSM and ITIM structural domains in the cytoplasmic region of PD-1. The phosphorylated ITSM then binds C-SH2 with high affinity, recruiting SHP2 to PD-1. The phosphorylated ITMM binds to N-SH2 and activates SHP2 [20, 21]. Subsequently, activated SHP-2 dephosphorylates TCR-associated CD3 and ZAP70 signalosomes, further attenuating TCR downstream signaling intensity and cytokine secretion such as IL-2 [7]. In bone marrow myelocytes, the PD-1-SHP-2 axis also restrains myeloid cell differentiation by impeding HOXA10 and IRF8 phosphorylation. Targeted deletion of PD-1 or SHP-2 in mice induces the differentiation of myeloid cells into monocytes with strengthened antigen presentation and T cell co-stimulation capabilities, thereby boosting immunity [22]. However, research also shows that, under chronic viral infections, SHP-2 is dispensable for T cell exhaustion and PD-1 signaling [23]. Additionally, the inhibitory target of SHP2 is likely the T-cell co-stimulatory receptor CD28. PD-1 can inhibit T cell function through SHP2-mediated dephosphorylation of CD28 rather than directly inhibiting TCR signaling [24]. This also indicates that, besides TCR, the major targets of PD-1 signaling need to extensively consider the role of CD28 or other co-stimulatory molecules. Therefore, based on the aforementioned molecular regulatory mechanisms of the PD-1/PD-L1 signaling pathway, it is not difficult to see that pathogen-infected cells utilize the PD-1/PD-L1 axis to promote the occurrence of immune inflammation in the local tumor microenvironment, breaking the immune balance of the organism and evading the attack of the host immune system. Blocking the PD-1/PD-L1 pathway can enhance the efficacy of T cells, making pathogen-infected cells more sensitive to immune checkpoint blockade therapy (Fig. 1).

The PD-1 molecule plays a crucial role in cellular adhesion and migration. Activated Tregs highly expressed PD-1, PD-1/PD-L1 signaling regulates T cell migration across lymphatic endothelial cells through PI3K/Akt in vivo [25]. The binding of PD-1 to PD-L1 also regulates the formation of memory T cells, affecting the memory and persistence of immune responses. Tissue-Resident Memory T Cells mediate protective immune responses and control tissue immune homeostasis in the human pancreas through the PD-1/PD-L1 inhibitory pathway [26]. Additionally, PD-1 can influence glycolysis and other metabolic pathways by inhibiting the downstream signal transduction of T cell signaling molecules, thereby obstructing cellular bioenergetics [27]. Moreover, PD-1 plays a key physiological role in both the central and peripheral nervous systems. Junli Zhao et al. find that PD-1/PD-L1 signaling in hippocampal neurons regulates synaptic transmission and cognitive behaviors. Inhibiting PD-1 alleviates the decline in learning and memory following traumatic brain injury [28]. Under physiological conditions, PD-L1 is widely expressed across numerous tissues, though its exact role in tissue and organ development and regeneration remains unclear. Recent research has uncovered that PD-L1 is abundantly present in breast stem cells and promotes the development and regeneration of mammary glands, offering new insights into the physiological functions of PD-L1 [29]. During pregnancy, the expression of PD-L1 in the placenta contributes to the maternal immune tolerance to fetal antigens and prevents immune attacks on the fetus [30]. A study has shown that PD-L1^+^ senescent cells, which are resistant to T cell surveillance, gradually accumulate with ageing. Blocking the PD-L1 signaling by PD-1 antibody activates CD8^+^ T cells to clear p16^+^ senescent cells as well as PD-L1^+^ senescent cell population in vivo, ameliorating ageing-related phenotypes in mice [31].

### Roles of PD-1/PD-L1 in transplantation and autoimmune diseases

The PD-1/PD-L1 signaling pathway is critical in autoimmune diseases, viral infections, transplantation and tumor immunity [32, 33]. Under a normal physiologic state, PD-1/PD-L1 signaling can effectively inhibit the excessive activation of immune cells, thereby avoiding severe persistent tissue damage and participating in maintaining immune tolerance to self-antigens. Disruption of the balance between PD-1 and PD-L1 can lead to the occurrence of various autoimmune diseases, including type 1 diabetes mellitus (T1DM), multiple sclerosis (MS), systemic lupus erythematosus (SLE), and rheumatoid arthritis (RA) [34].

### Transplantation

**Fig. 2:**
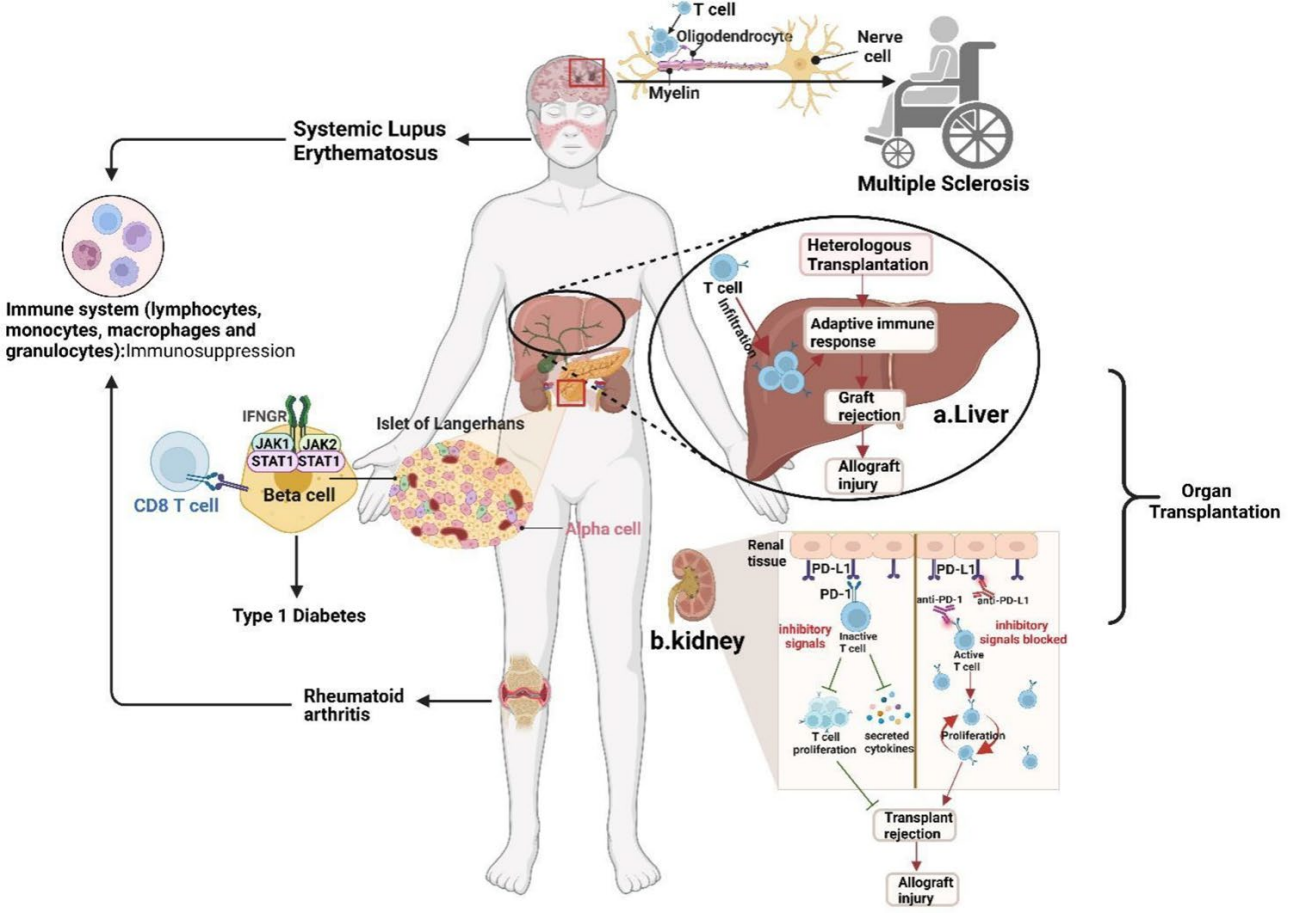
Role of PD-1/PD-L1 in transplantation and autoimmune diseases. During organ transplantation, PD-1 is highly expressed on the surface of infiltrating T cells in grafts. The negative regulatory signal mediated by PD-1/PD-L1 can inhibit the excessive activation of T cells, induce immune tolerance, and effectively reduce the immune rejection between the host and the donor after surgery. Blocking PD-1/PD-L1 promotes proliferation of infiltrating T cells in grafts, exacerbating post-transplant immune rejection reactions and inducing severe and persistent tissue damage. Similarly, the disruption of the balance between PD-1 and PD-L1 signals can lead to the occurrence of many autoimmune diseases, such as type 1 diabetes mellitus (T1DM), multiple sclerosis (MS), systemic lupus erythematosus (SLE), and rheumatoid arthritis (RA). Figure created with BioRender

After transplantation, due to mutual intolerance between the graft and the host, it often leads to reciprocal attack and rejection of immune cells between the recipient’s immune system and the donor graft. The most common reaction is the host versus graft reaction (HVGR) during organ transplantation, while individual patients also experience a graft versus host reaction (GVHR) where T/B immune cells in the graft attack the host [35]. The negative regulatory signals mediated by PD-1/PD-L1 can inhibit the activation and proliferation of T cells, induce T cell dysfunction, and effectively reduce immune rejection between the host and the donor [36]. For example, the PD-1/PD-L1 signaling pathway participates in the rejection of human allografts. Shi’s team performs biopsies on transplanted livers of recipients experiencing acute rejection induced by inflammation. They note that PD-L1 expression is increased in the portal veins and lobular regions of the transplanted liver, as well as high expression of PD-1 on infiltrating T cells within the graft, with critical interactions between the donor PD-L1 and recipient PD-1. Blocking PD-1/PD-L1 enhances the proliferation of infiltrating T cells within the graft, exacerbating the immune rejection after transplantation [37]. Similar experimental results are observed in the mouse model of renal allograft rejection. PD-1 is widely expressed on most T cells within the transplanted kidney, and the upregulation of PD-L1 expression on renal tubular epithelial cells may inhibit T cell activation and proliferation, reducing T cell-mediated excessive immune damage [38]. These results suggest that upregulation of PD-1/PD-L1 expression may be a negative feedback protective mechanism in the immune response to parenchymatous organ transplantation, reducing postoperative immune reactions (Fig. 2).

Clinical data has shown that recipients with recurrent and metastatic liver cancer after liver transplantation may experience immune-related adverse reactions after undergoing immunotherapy, leading to a poor prognosis for the patients. For example, Friend et al. describe two patients with recurrent and refractory hepatocellular carcinoma (HCC) after orthotopic liver transplantation, who rapidly develop irreversible acute liver rejection and eventually die shortly after receiving the PD-1 inhibitor nivolumab [39]. Likewise, Fisher et al. also evaluated the safety and efficacy of immunotherapies such as PD-1 inhibitors in 57 metastatic cancer patients with a history of solid organ transplantation (liver, kidney, and heart). The results demonstrate that 37% of the patients experience organ rejection reactions, and 14% die due to graft rejection. Among them, the rejection rate is highest after using nivolumab, and patients who use Ipilimumab have the highest rate of malignant tumor progression. Kidney transplant recipients exhibit the highest rejection rate, followed by liver transplant recipients [40]. In a clinically relevant case, a 63-year-old female patient with type 1 diabetes develops pulmonary metastatic melanoma 10 years after kidney transplantation. Within one week of initiating first-line treatment with nivolumab for melanoma, the patient experiences acute renal allograft rejection, renal failure, and concurrent diabetic ketoacidosis [41], suggesting a potential association between the use of anti-PD-1 antibodies and allograft rejection. Therefore, the PD-1/PD-L1 pathway is critical for inducing and maintaining transplant tolerance.

So how can we apply immunotherapy to exert anti-tumor effects in the body without triggering immune rejection? I believe that the first step is to identify biomarkers that can predict the risk of transplant rejections and tumor responses. For example, recipients experiencing acute rejection show high expression of PD-L1 in biopsy samples. Based on this, we can characterize the expression of PD-L1 on grafts before using anti-PD-1 drugs and select the appropriate immune checkpoint inhibitor for blockade therapy according to the patient’s individual condition and the possibility of rejection. Secondly, when administering immune checkpoint blockade therapy to patients with a history of organ transplantation, we must be cautious and consider the risk of immune rejection in the transplant rather than purely the anti-tumor treatment effects. Finally, it is important to develop novel immunosuppressive therapies to minimize the use of ICI therapy and devise personalized immunosuppression plans for patients. For example, targeting PD-1 with appropriate agonists (such as PD-L1 Ig fusion protein) can reduce rejection reactions and optimize therapeutic approaches. Thus, PD-1/PD-L1 signaling may have a major role in graft tolerance and the prevention of late rejection after transplantation.

### Autoimmune diseases

PD-1/PD-L1 also has significant clinical relevance to autoimmune diseases (Fig. 2).

**Type 1 diabetes mellitus (T1DM)** is a severe auto-immune disease caused by severe destruction of insulin-producing β-cells in the pancreas, resulting in hyperglycemia [42]. Recently, it has been discovered that IFN-α and IFN-γ can induce PD-L1 expression in human islet cells, which can limit the immunological killing of islet cells after the activation of autoreactive T cells, thereby preventing autoimmune damage. Blocking JAKs or IRF1 (the recommended strategies for T1DM treatment) can prevent PD-L1 induction [43]. During the progression of T1DM in NOD (non-obese diabetic) mice, T cells attack pancreatic islet cells, causing the loss of beta cells. PD-L1 expression is significantly increased in beta cells that can withstand autoimmune attacks and survive for a long time, thus preventing type 1 diabetes [44]. Similarly, platelets genetically engineered to overexpress PD-L1 accumulate in pancreas and inhibit the activity of islet-specific autoreactive T cells, reversing new-onset T1D [45]. PD-1 also regulates autoimmunity to limit the development of diabetes by inhibiting the proliferation and infiltration of pancreatic T cells. Single-cell RNA-seq and TCR-seq of spontaneous T1D vs. anti-PD-1 induced T1D show that PD-1-induced T1D significantly increases the proliferation of exhausted/effector-like T cells [46]. When using PD-1/PD-L1 inhibitors, the PD-L1 molecule on β cells can’t bind to PD-1 on autoreactive T cells, while the inflammation-stimulated autoreactive T cells are over-activated, greatly promoting the progression of diabetes. This means that PD-1 or PD-L1 antibodies used in cancer immunotherapies may induce autoimmune insulin-dependent diabetes mellitus. For instance, a case-review analysis by Stamatouli et al. [47] found that among 2960 patients treated with immune checkpoint inhibitors, 27 cases (an incidence of 0.9%) experienced acute episodes of insulin-deficient diabetes. Of these 27 diabetic patients, 59% also have diabetic ketoacidosis, 42% have pancreatitis, 85% exhibit rapid loss of beta-cell function, manifested by rapid progression of hyperglycemia. Moreover, this type of immune-associated diabetes occurs more frequently in patients receiving anti-PD-1/PD-L1 than in those treated solely with anti-CTLA-4, indicating that T1DM is primarily associated with anti-PD-1/PD-L1 immunosuppressive therapy. Increasing clinical data suggests that in a majority of cancer patients, the use of PD-1 or PD-L1 antibody treatments can induce diabetic ketoacidosis (DKA) or trigger acute onset of type 1 diabetes shortly after initiation, significantly reducing the clinical efficacy of antitumor therapies [48, 49] Therefore, based on the above research and case analysis, we know that tumors associated with autoimmune diabetes have no advantage during immunotherapy. For patients with a previous history of diabetes, the risk of anti-PD-1/PD-L1-induced autoimmune diabetes should be evaluated before choosing immunotherapy. If biomarkers related to the progression of diabetes can be identified for personalized drug management, it would contribute to reducing immune-related adverse effects in patients and even preventing diabetes onset. Meanwhile, patients should be closely followed up during first-line immunotherapy, with routine blood glucose monitoring before each administration and periodic testing for islet autoantibodies to prevent DKA.

**Systemic lupus erythematosus (SLE)** is one of the most common systemic auto-immune diseases, which is characterized by the destruction of self-antigen immune tolerance and excessive accumulation of self-antibodies, resulting in multi-organ damage [50]. Among them, type I interferons and Toll-like receptors regulate PD-1 and its ligand PD-L1 expression by activating NF-κB or STAT1, playing a crucial role in SLE pathogenesis [51]. PD-1 is significantly upregulated on CD4 T cells and in a pathogenic activation state during SLE progression, the mitochondrial membrane potential of PD-1^+^CD4 T cells shows abnormal activation, thus promoting the progression of SLE. When using cell membrane biomimetic membrane nanoparticles with high PD-L1 expression (IM-MNPs/DXM, a functional-driven, disease-relevant CD4 T cell-targeted drug carrier), it kills pathogenic CD4 T cells and inhibits the expression of pro-inflammatory factors, reshaping the balance between the effector T cells and Tregs in the microenvironment, thereby alleviating SLE [52]. Similarly, in SLE mouse models, PD-1 blockade can activate suppressive CD8^+^ T cells, thereby delaying the onset of renal disease and the progression of SLE, ultimately leading to an increase in the survival rates of the mice [53]. Recent studies have found that anti-PD-1 autoantibodies are elevated in the serum of newly diagnosed SLE patients, promoting T cell proliferation and positively correlating with the disease activity of SLE [54]. This indicates that PD-1 antibodies may accelerate the severity of lupus by blocking the biological functions of PD-1 signaling, providing new insights into the regulatory pathways of PD-1 in SLE. Furthermore, the frequency of PD-L1 expressing neutrophils in SLE patients is significantly increased, it is positively correlated with disease activity and severity, serving as a novel indicator for evaluating SLE [55]. We hypothesize that neutrophils expressing PD-L1 may inhibit T cell-mediated immune responses through multiple mechanisms, it may be a negative feedback mechanism to prevent potential tissue damage caused by excessive autoimmune responses in SLE patients. In conclusion, the regulatory function of the PD-1/PD-L1 pathway in SLE patients is complex and may be related to PD-1 polymorphisms or the quantity and immunosuppressive function of Tregs in SLE. It may also be context-dependent, requiring further preclinical studies to elucidate its contributions to immune regulatory functions.

**Multiple sclerosis (MS)** is an inflammatory demyelinating disease of the central nervous system mediated by immune dysregulation, mainly characterized by motor and sensory disorders, ataxia, etc. PD-1/PD-L1 signaling plays a key role in the pathogenesis of MS by downregulating the immune responses of autoreactive T cells in the central nervous system [56]. Previous studies have shown increased expression of PD-L1 and PD-1 in the central nervous system of animal models, such as experimental autoimmune encephalomyelitis (EAE). Blocking PD-1 in EAE leads to antigen-specific T cell expansion and increased cytokine production, thereby enhancing the autoimmune response and accelerating the progression of EAE symptoms [57]. Antje Kroner et al. find that the PD-1 intron 7146G/A polymorphism is associated with disease progression in MS. In patients carrying the PD-1 polymorphism, mutations in the regulatory region of the PD-1 gene may affect PD-1 signal transduction, thereby impairing the function of inhibiting T cell proliferation mediated by PD-1 and accelerating disease progression in MS patients [58]. Koto et al. observe that PD-1^+^ CD8 T cells were reduced in the peripheral blood of MS patients during the MS disease remission state. Conversely, during MS relapses, IFN-β stimulation induces the enrichment of PD-1^+^CD8 T cells in the cerebrospinal fluid, which contributes to self-immune regulation and is correlated with a positive response to subsequent treatment [59]. In summary, these findings suggest that PD-1/PD-L1 plays a protective role in EAE and can be used as a new target for therapeutic intervention.

**Rheumatoid arthritis (RA)** is an autoimmune systemic inflammatory disease with a complex pathogenesis, primarily characterized by the production of autoantibodies such as immunoglobulin G (IgG) and anti-citrullinated protein antibodies (ACPA). Overexpression of PD-1/PD-L1 in macrophages and synovial T cells in RA patients has been significantly associated with disease progression and a lower overall survival rate [60, 61]. Wan et al. find that IFN-γ and TNF-α selectively induce overexpression of negative co-stimulatory molecules such as PD-1 and CTLA-4 in synovial T cells and APCs of RA patients. However, the overexpression of these negative co-stimulatory molecules, which downregulates T cell responses, presents a clear contradiction with the persistent activation of autoreactive T cells in RA. Further research reveals that this functional abnormality of negative co-stimulatory molecules appears to be mediated by soluble PD-1 (sPD-1), which is secreted abundantly in the serum and synovial fluid of RA patients. Specifically, the PD-1 alternative splice variant (PD-1Δex3) derived from RA T cells produces sPD-1 and antagonizes the inhibitory efficacy of membrane PD-1 on T cells, which is specific to RA and unrelated to the activation state of T cells. The use of a human recombinant fusion protein of soluble PD-1 (hPD-1-Ig) can effectively block the immunosuppressive function mediated by membrane PD-1 and PD-L1 [62]. This study provides new evidence for the role of PD-1 soluble factors in the pathogenesis of RA and other autoimmune diseases, contributing to the development of new therapeutic strategies. RA-related inflammatory factors are also involved in PD-L1 regulation, leading to the high expression of PD-L1 in RA. For example, Haiyan Zhou et al. evaluated circulating T cell subsets and cytokine expression profiles in RA patients. The results exhibited a remarkable increase in pro-inflammatory cytokines such as MCP-1, IL-6, and IL-1β in RA. PD-1^+^ T_EM_ (effector memory T cell), PD-1^+^ T_CM_ (central memory T cell), and PD-1^+^ Tfh (follicular helper T cells) subsets were positively correlated with RA disease activity DAS28, suggesting a potential as a new indicator for RA treatment [63]. Additionally, Kostine et al. conducted a retrospective assessment of the incidence of rheumatic immune-related adverse events (irAEs) and the tumor response rates in 524 patients receiving ICIs. The results show that 35 patients (6.6%) are referred to rheumatology, nearly all rheumatic irAEs occur in the context of anti-PD-1/PD-L1 antibody treatments, and patients with rheumatic irAEs show a higher tumor response rate [64].

These above findings provide preliminary evidence for the regulatory functions of PD-1/PD-L1 signaling in autoimmune diseases, the exact mechanisms still need further elucidation in future studies. Traditional approaches involving gene knockout or inhibition of PD-1/PD-L1 may cause activated immune cells to become uncontrolled by immune checkpoints, eventually exacerbating autoimmunity mediated by T cells and B cells in autoimmune diseases. So, is there a therapy that can preserve normal immune cells while targeting and eliminating dysfunctional immune cells? In recent years, in addition to the research targeting PD-1/PD-L1 checkpoint alone, researchers have found that depletion of PD-1^+^ T lymphocytes also significantly improved the progression of autoimmune diseases. For example, targeted immunotoxins against PD-1 (composed of the anti-PD-1 single-chain variable fragment, the albumin binding domain and the Pseudomonas exotoxin) selectively bind to and eliminate PD-1 expressing cells, as well as reduce the proliferation of autoreactive T cells in inflamed organs [65]. Further studies found that in the T1D mouse models, immunotoxin-treated PD-1 cell depletion significantly inhibited the onset of T1D and prolonged the mice’s survival. In the EAE mouse models, treated mice even regained walking abilities with no signs of relapse. Importantly, each mouse retains normal immune responses and their immune functions are not compromised. The efficacy of immunotoxin-induced PD-1 cell depletion can be further improved through pharmacokinetic-pharmacodynamic optimization, thereby preventing autoimmune diseases. In the future, targeted depletion of PD-1^+^ cells may be an efficient and widely applicable treatment to alleviate autoimmunity.

### Roles of PD-1/PD-L1 axis in tumors

Tumor invasion, metastasis, and recurrence are complex processes with multi-gene involvement and multi-step development, which are formed by the interaction of intrinsic factors and external factors such as viral infection, and represent a dynamic interplay of clearance, balance, and evasion between the immune system and the tumor [66]. The interaction between PD-1 and PD-L1 can transmit inhibitory signals within cells, suppressing lymphocyte proliferation and activation, thereby diminishing immune capacity. Consequently, tumor cells often promote their own occurrence and growth by ‘hijacking’ this axis [67, 68]. When tumor-infiltrating lymphocytes (TILs) recognize tumor antigens, they initiate “adaptive resistance” mediated by the PD-1/PD-L1 pathway to assist tumor immune evasion and distant metastasis [69]. Specifically, this is manifested as follows, (I) PD-1/PD-L1 interaction suppresses the activation and proliferation of T cells, promoting T cell dysfunction and apoptosis. (II) PD-1/PD-L1 interaction enhances the function of regulatory T cells (Tregs) and induces immune tolerance. (III;) PD-1/PD-L1 interaction promotes the polarization of TAM and other immune cells into tumor-promoting phenotypes, facilitating immune escape and cancer progression. (III) Signaling of PD-L1 within cancer cells may prevent the apoptosis of tumor cells themselves, and the interaction of PD-L1 with CD80 can suppress the immune response, etc [70].

### PD-1/PD-L1 axis promotes tumor occurrence and development

#### PD-1/PD-L1 interaction promotes effector T cell exhaustion and apoptosis

**Fig. 3:**
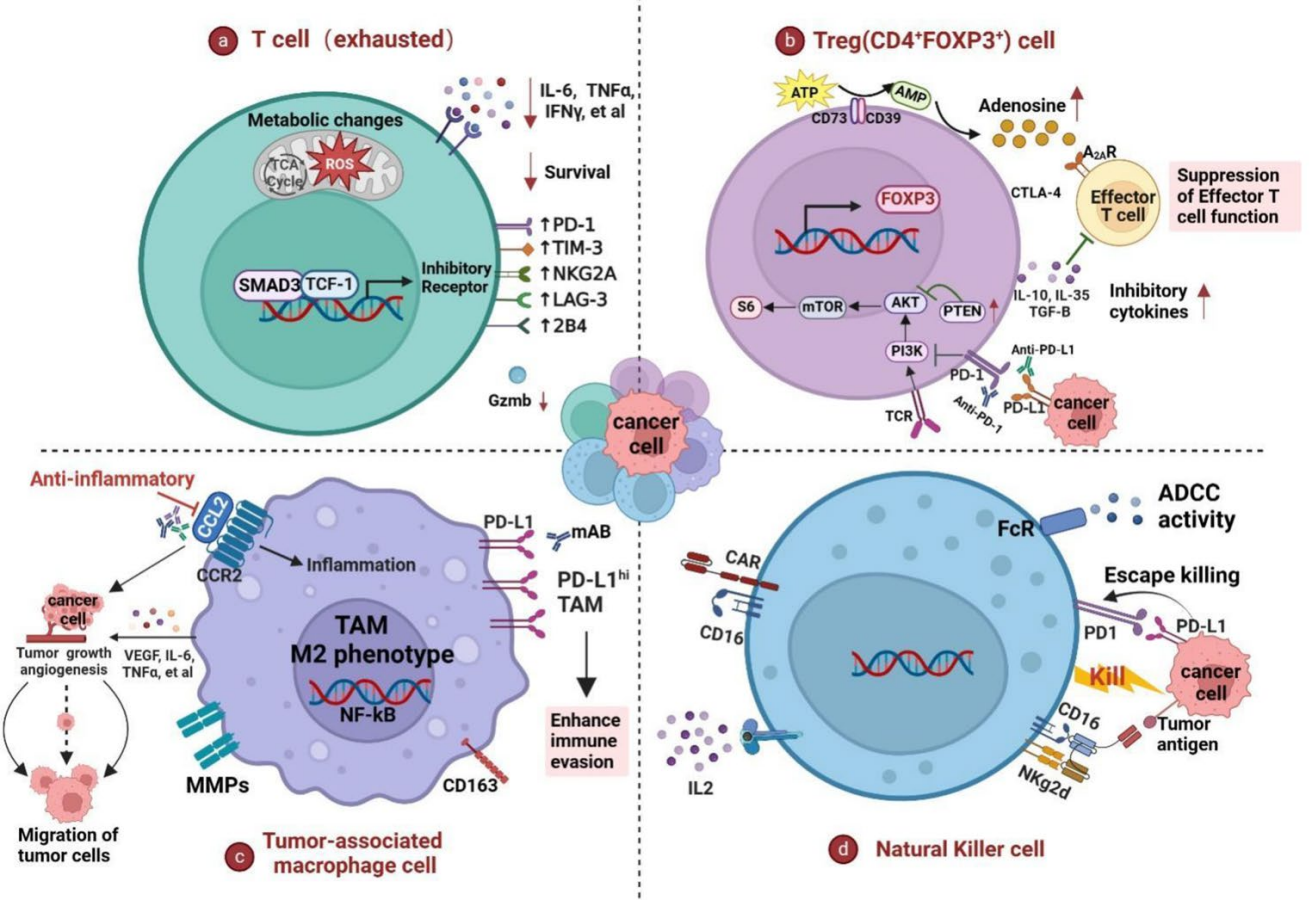
The regulation of PD-1/PD-L1 signaling on immune cells. **a**. The PD-1/PD-L1 pathway promotes the exhaustion and apoptosis of effector T cells. Tex cells are characterized by increased expression of highly inhibitory receptors, including PD-1, LAG3, and TIGIT, decreased cytokine production such as TNF, IL-2, and IFN-γ, metabolic alterations, and impaired proliferative capacity and survival. **b**. PD-1/PD-L1 promotes the generation and development of induced Tregs (iTregs) by reducing the phosphorylation of PI3K/Akt/mTOR and S6, while enhancing PTEN, thus enhancing the immune suppression functions of Treg cells and inducing immune tolerance. **c**. PD-1/PD-L1 can promote the polarization of tumor-associated macrophages (TAM) toward the M2 phenotype, releasing large amounts of fibroblast growth factor, VEGF, TNF-α, and other cytokines to promote angiogenesis and support immune suppression, invasion, and metastasis of cancer cells, accelerating cancer progression. **d**. PD-1 on NK cells binds to PD-L1 on cancer cells, inhibiting the degranulation and cytotoxic function of NK cells, decreasing their ability to kill tumor cells, and promoting tumor immune escape. The use of PD-1 and PD-L1 inhibitors may reactivate the anti-tumor immune response of the above immune cells. Figure created with BioRender

T cells play a major role in tumor immunity, as well as being an important mediator of the activation or inhibitory signals transmitted by T cell co-inhibitory receptor molecules. PD-1, a checkpoint receptor expressed on T cells, causes T cell exhaustion and prevents excessive immune stimulation when it receives signals from its ligand PD-L1 [71]. Under normal conditions, PD-L1 is lowly expressed on most cells, but it is highly expressed on the surface of human cancer cells. Prolonged exposure to viral antigen stimulation may result in T cell subset dysregulation or dysfunction, being in a state of diminished responsiveness, known as “T cell exhaustion” (Tex cells) [72]. Tex cells have impaired killing effects and undergo different patterns of differentiation. They are characterized by increased co-expression of inhibitory receptors (PD-1, LAG3, and TIGIT, etc.), reduced production of cytokines (IL-2, TNF, and IFN-γ, etc.), metabolic changes, and impaired proliferation capacity and survival rate [73]. Finally, T cells are completely exhausted and undergo apoptosis, many mechanisms can explain this process (Fig. 3a).

Upon the binding of PD-1 on tumor-infiltrating lymphocytes to PD-L1 on tumor cells, an inhibitory signal is transmitted to T cells, which can block the TCR signal cascade responses and its co-stimulatory signal transduction, inducing dysfunction and even exhaustion of effector T cells, thus causing tumor cell immune escape [74]. Previous studies mostly considered that PD-1 is preferentially expressed on chronic virus-specific CD8 T cells and is a major regulator of T cell exhaustion and apoptosis [75]. PD-1 blocks the anti-apoptotic gene Bcl-xL expression by inhibiting Akt activation, which in turn reduces T cell survival rates [76]. Blocking the PD-1/PD-L1 interaction with mAb restores T cell proliferation, cytokine secretion, and cytotoxicity, to a certain extent reversing T cell exhaustion [77], thus identifying PD-1 as a promising therapeutic approach for restoring HIV-specific exhausted T cells. In tumors or chronic viral infections, PD-1 is overexpressed on functionally exhausted T cells. Persistent activation and PD-1 signaling can inhibit glycolysis and mitochondrial metabolism, this biological energy deficiency exacerbates early T cell dysfunction and exhaustion [78], indicating that the intensity of PD-1 signaling also determines the severity of T cell exhaustion. PD-1 signaling also prevents the conversion of CD8^+^ central memory T cells (T_CM_) into CD8^+^ effector memory T cells (T_EM_) [79], reducing the long-term immune memory that may prevent future metastatic diseases. Additionally, PD-L1 on tumor cells also interacts with CD80 on activated T cells, decreasing the activation of effector T cells and even inducing apoptosis of activated T cells [80]. Diskin et al. found that, under the stimulation of tumor antigens and inflammatory cytokines, the expression of PD-L1 in tumor-infiltrating T cells can be activated. At this time, PD-L1 acts as both ligand and receptor to transmit forward and backward signals to regulate the immune responses in tumor tissues. Specifically, PD-L1 in T cells induces intracellular signaling, its inhibitory effect is the same as that of PD-1. PD-L1^+^ T cells also promote the differentiation of STAT6-dependent M2 type macrophages and inhibit the function of adjacent effector T cells through the typical PD-L1/PD-1 axis [81]. Thus, blocking PD-1/PD-L1 can reduce T cell apoptosis and promote anti-tumor immunity.

PD-1/PD-L1 blockade therapy is the foundation of current tumor immunotherapy, but the specific anti-tumor immune mechanisms are still unclear. It was previously generally believed that the anti-tumor immune effect was exerted by restoring the activity of exhausted T cells or inhibiting the activity of Treg cells within the tumors [82]. In recent years, there has been ongoing controversy regarding whether exhausted T cells can be reverted back to an active state, as increasing evidence suggests that blockade of the PD-1/PD-L1 axis may not be able to “rescue” terminally exhausted T cells. So, what specific type of T cell can actually respond to PD-1/PD-L1 inhibitor therapy? Margaret et al. found that new T cell clones appeared after anti-PD-1 therapy, recruiting tumor-infiltrating lymphocytes to the tumor microenvironment [83]. Miller et al. found that differences among exhausted CD8^+^ T cell subsets affected their anti-tumor functions and the efficacy of ICI therapy. Compared to terminally exhausted T cells, progenitor exhausted CD8^+^ TILs are better at controlling tumor growth, melanoma patients with a higher proportion of progenitor exhausted CD8^+^ TILs have a longer response to PD-1 blockade therapy [84]. Subsequently, Chen et al. discovered that combining Alarmin HMGN1 peptide with PD-L1 blockade therapy promotes the proliferation and activates the anti-tumor activity of stem-like/progenitor-exhausted CD8^+^ T (Tstem/Tpex) cells [85]. Therefore, a detailed understanding of T cell exhaustion, especially the subset of stem-like CD8 T cells, is crucial for developing effective combination immunotherapies. According to the latest research by Lilin Ye et al., there exists a group of TCF-1^+^ TOX^−^ tumor-specific memory CD8^+^ T cells (T_TSM_) in tumor-draining lymph nodes (TdLN), which have a typical memory T cell phenotype and potent anti-tumor capabilities with less exhaustion, representing the real CD8^+^T cell subpopulation that responds to PD-1/PD-L1 inhibitors [86]. Treatment with PD-L1 inhibitors promoted substantial expansion of TdLN-T_TSM_ cells, resulting in the accumulation of TPEX (TCF-1^+^TOX^+^, exhausted precursor T cells) and TEX (TCF-1^−^ TOX^+^) cells in TME. Injecting PD-L1 inhibitors into TdLNs effectively inhibits tumor growth, the anti-tumor effects of PD-L1 inhibitors disappear after the surgical excision of TdLNs, providing mechanistic support for preoperative immune neoadjuvant therapy. A study by Zhu Bo’s team also redefined the unique hematopoietic development pattern in the tumor context. Erythroid precursor cells CD45^+^EPC, already on the erythroid development trajectory, shifted towards differentiation into tumor-promoting erythroid-derived myeloid cells (EDMCs) under the “coercion” of tumors [87]. The expansion of EDMCs was positively associated with intra-tumor PD-L1 expression, T cell exhaustion and immune tolerance in TME, which significantly inhibited antitumor immune responses of CD8^+^ T cells and greatly reduced the efficacy of immune checkpoint inhibitors, thus providing valuable prognostic biomarkers and a new therapeutic goal for clinical ICI treatment. Therefore, the above findings indicate that although the extent of T cell exhaustion in the TME of patients with effective ICI therapy is reduced, this does not necessarily result from exhausted T cells regaining vitality. Further exploration into how ICI therapy improves and restores the function of exhausted T cells is needed.

### PD-1/PD-L1 interaction promotes the generation and function of Treg cells

Regulatory T cells (Tregs) are a special subset of CD4^+^ T cells. Tregs can produce certain pro-inflammatory cytokines in the inflammatory microenvironment and suppress autoimmunity by secreting cytokines including adenosine, TGF-β1, and IDO [88], thereby mediating tumor cell immune escape and promoting tumor growth. As many tumors are highly infiltrated by immunosuppressive Treg cells, this may further trigger strong immunosuppression and hinder the functions of effector T cells, which is associated with poor prognosis in various cancers [89].

Recent research has revealed that the PD-1 or PD-L1 pathway can promote the generation of Treg cells and enhance their function in TME, which is closely correlated with the immune escape of tumor cells and tumor development. For instance, Francisco et al. discovered that PD-L1 itself can promote the generation and development of induced Tregs (iTregs) by reducing the phosphorylation of Akt–mTOR, S6, and ERK2, while enhancing PTEN, thereby strengthening the immune suppressive functions of Tregs and suppressing the responses of autoreactive T cells [90]. High levels of PD-L1 expression usually lead to an increase in the number of Foxp3^+^ Treg cells [91]. Patients with tumors infiltrated by a large number of Foxp3^+^Treg cells often have a poor prognosis [92, 93]. Removing Foxp3^+^Treg cells from tumor tissues can enhance anti-tumor immunity, hence a combined strategy of blocking PD-L1/PD-1 while depleting Tregs might show promise in improving treatment outcomes for these patients. Similar to effector T cells, Treg cells also express PD-1, and their survival and functions in the TME depend on TCR and CD28 signals [94]. The regulation of these signaling molecules promotes the induction and maintenance of Treg cells, inhibits T cell responses, and participates in the distant metastasis and recurrence of tumors. PD-1 expression on Treg cells can also promote the transformation of naive CD4^+^T cells into Treg cells by inhibiting the phosphorylation of Smad3 by cyclin-dependent kinase (Cdk2), thereby suppressing normal immune responses [95]. (Fig. 3b).

During the process of tumor immunotherapy, the application of PD-1 or PD-L1 antibody blockade therapy affects the proliferation and function of Tregs, and may even trigger drug resistance and hyper-progressive disease (HPD). On the one hand, PD-1/PD-L1 antibodies inhibit the differentiation, proliferation, and function of Tregs induced by PD-1/PD-L1. For example, PD-L1, which is upregulated in glioblastoma (GBM), induces and maintains Treg proliferation by interacting with PD-1 on Tregs, thereby exerting its immunosuppressive function. Using nivolumab (a PD-1 inhibitor) suppresses PD-1/PD-L1-induced Treg expansion, significantly prolonging the survival of glioma patients [96]. Similarly, Gambichler et al. evaluate the effects of PD-1 antibody blockade treatment on Treg subsets in the blood of melanoma patients, showing that the use of nivolumab or pembrolizumab leads to a rapid decrease in PD-1^+^ Tregs in peripheral blood, reducing the risk of melanoma-specific death [97]. Conversely, in some patients with poor innate immunity, T cells remain at a low level. When PD-L1 expression is upregulated on tumor cells, immune therapy mediated by PD-1/PD-L1 antibodies may enhance the TCR signaling and CD28 signaling of Tregs, thereby activating Tregs and enhancing their inhibitory functions, resisting tumor immunity and ultimately leading to the occurrence of hyper-progressive disease (HPD). For example, PD-L1/PD-1 interactions restrict effector regulatory T cells (eTregs) during homeostasis and infection. Under steady-state conditions, activated T cell receptors promote the production of PD-1 in eTregs, and blockade of PD-1 signaling enhances the activity of eTregs. During Toxoplasma infection, IFN-γ promotes PD-L1 upregulation in bone marrow cells through STAT1, associated with a reduction in the Treg population. Blockade of PD-L1 prevents the loss of eTregs and mitigates the development of immunopathology [98]. Anti-PD-1 mAbs treatments cause proliferation of PD-1^+^ Treg cells in some gastric cancer patients, thereby increasing Treg cell-mediated immunosuppressive activity and promoting disease hyper-progression during PD-1 blockade therapy [99]. Studies have shown that Tregs efficiently uptake lactic acid from the tumor microenvironment through MCT1, convert lactic acid into malic acid and citric acid, and then transfer it to mitochondria to participate in the tricarboxylic acid cycle, enhancing PD-1 expression and Treg immunosuppressive function, and reducing the effectiveness of immunotherapy [100]. We speculate that this may also be a reason for the occurrence of disease hyperprogression. Lactate can also regulate the generation of Tregs by lactylation at Lys72 of MOESIN, thereby improving the interaction between MOESIN and TGF-β/SMAD3 signaling. Combined treatment with anti-PD-1 and lactate dehydrogenase inhibitors has a stronger anti-tumor effect [101], thereby preventing hyper-progression disease. This suggests that in immunotherapy, the exhaustion of PD-1^+^ Tregs or weakening their immunosuppressive function combined with ICI therapy can be potential interventions for HPD, thus improving the treatment efficacy. In conclusion, these studies indicate that Tregs serve as biomarkers to predict the occurrence of HPD in anti-PD-1/PD-L1 immunotherapies. More research is still needed to clarify the dual effects of PD-1/PD-L1 antibody blockade therapy on Tregs, which may depend on the regulation of multiple factors, such as differences in Treg subgroups, the effect of Tregs on different target cells (such as APCs) in chronic viral infections or cancer, and different downstream molecular mechanisms of regulation, etc. In the future, we should deeply explore the complex relationships between PD-1/PD-L1 and Tregs in tumor immunity and combination therapy to provide new strategies for improving the efficacy of immunotherapies for solid tumors.

### PD-1/PD-L1 interaction promotes cancer progression by polarizing tumor-associated macrophages (TAMs)

Macrophages are mainly divided into M1 and M2 types. M1 type macrophages exert immune surveillance functions by recruiting chemokines (CXCL10) and activating other immune effector cells to target cancer cells indirectly. In contrast, M2 type macrophages promote immunosuppression, cancer cell invasion and metastasis, and angiogenesis by releasing various cytokines, playing a key role in tumor progression [102]. Tumor-associated macrophages (TAMs) refer to a specific type of macrophage that infiltrates into tumors, typically characterized by two polarization states but whose functional phenotype is closer to that of M2 macrophages [103]. The autophagosome TRAP released by tumor cells induces macrophages to polarize to the M2 phenotype and upregulates PD-L1 and IL-10 expression in TAMs through the TLR4-MyD88-p38-STAT3 signaling pathway, suppressing T cell proliferation and promoting tumor growth [104]. TAM not only secretes basic vascular endothelial growth factor A, fibroblast growth factor and adrenomedullin to stimulate angiogenesis but also degrades the extracellular matrix, providing nutrition to tumor cells and supporting distant metastasis of tumors [105].

TAMs secrete various cytokines (such as IFN-γ, TNF) to upregulate the PD-L1 expression in tumor cells. With the growth of tumors, PD-1 expression in TAMs increases exponentially [106]. PD-1^+^ TAMs expressed more CD206, CD11c, and fewer MHC class II molecules [107], suggesting that PD-1 promotes macrophage polarization towards the M2 phenotype. When PD-1 is deficient, it drives macrophage polarization to the M1 phenotype by increasing the phosphorylation of STAT1/NF-κB p65 and downregulating p-STAT6, ultimately leading to richer M1 macrophage infiltration and higher levels of pro-inflammatory cytokine secretion [108]. However, due to long-term interaction with macrophages, CD8^+^ T cells may mediate lymphocyte trapping, making it difficult for lymphocytes to migrate and infiltrate into tumor islets [109]. Thus, macrophage exhaustion may reactivate CD8^+^ T cell migration into tumor islets, enhancing the efficacy of anti-PD-1 therapy. Moreover, in patients with various cancers, PD-L1 induces an immune-suppressive phenotype in macrophages through negative signaling, usually associated with poor prognosis. Anti-PD-L1 treatment can reverse this phenotype and induce macrophage-mediated anti-tumor activity [110, 111]. Studies in nearly 500 cases of NSCLC have found that 80% of CD68 macrophages highly expressed PD-L1, these patients had longer overall survival (OS) when treated with anti-PD-1/PD-L1 axis therapy [112]. In hepatocellular carcinoma cells, tumor-specific SLFN11 inhibits hepatocellular carcinoma proliferation and metastasis, knockdown of SLFN11 in HCC cells promotes macrophage migration and polarization towards the M2 phenotype in a CCL2 cytokine-dependent manner, thereby activating the NF-κB pathway, upregulating the expression of PD-L1 and accelerating tumor progression. Pharmacological antagonism of the CCL2/CCR2 signals can enhance the antitumor effect of anti-PD-1 monoclonal antibodies [106]. The Hippo signaling pathway reduces T cell infiltration by directly regulating IL-34 transcription and PD-L1 expression in TNBC, thereby promoting TAM recruitment and reshaping the tumor immune microenvironment. Targeting TAMs with IL-34/CSF-1R inhibitors enhances the antitumor efficacy of immune checkpoint inhibitors [113]. IFN-γ, secreted by inflammatory cells such as macrophages and NK cells, upregulates PD-L1 expression on tumor cells [114]. With the upregulation of PD-L1 expression on tumor cells, it will produce adaptive resistance to IFN-γ released by cytotoxic T lymphocytes, thus forming a vicious cycle that aggravates disease progression and produces primary resistance to ICIs treatment.

### PD-1/PD-L1 axis inhibits NK cell killing function to promote immune escape

Natural killer cells (NK) originate from pluripotent hematopoietic stem cells in the bone marrow, which can recognize and kill tumor or virus-infected cells. They also coordinate immune responses by releasing immunomodulatory cytokines such as PGE2, IDO, and TGF-β, serving as the first line of defense against viral infections and preventing malignant transformation of cells, having a crucial role in immune surveillance [115]. Therefore, enhancing the quantity and killing capacity of NK cells in cancer patients contributes to improving the effects of anti-tumor immunotherapy.

In recent years, there has been an increasing exploration of NK cell- based tumor immunotherapy. However, it is still unclear regarding the mechanisms of PD-1/PD-L1 molecule expression on NK cells, their impact on NK cell functions, and their role in regulating anti-tumor immunity. Most research has demonstrated that, in addition to T cells, the PD-1/PD-L1 axis also inhibits NK cell-induced antitumor immunity in vivo. Joy Hsu et al. find that PD-1 is highly expressed on activated NK cells, and its binding to PD-L1 on cancer cells inhibits NK cell degranulation and cytotoxic functions, thereby reducing its antitumor immune responses, promoting tumor immune evasion and progression. Using PD-1 and PD-L1 inhibitors reactivates NK cell anti-tumor responses [116]. Huang et al. find that PD-1 signal transduction on the NK cell inhibits lytic granule polarization and disrupts the “ inside-out” signal of integrin, suppressing the cytotoxic effects of NK cells, such as granzyme secretion, and reducing their ability to kill tumor cells [117] (Fig. 3d). However, Judge et al. find that under various heterogeneous conditions that stimulate high NK cell activation in vitro and in vivo, there is no significant expression of PD-1 on NK cells of humans and mice [118]. This contradictory phenomenon has prompted us to wonder, if NK cells do not express PD-1 themselves, how does the PD-1/PD-L1 pathway inhibit NK cell activity? Recent studies have revealed that in leukemia mouse models, PD-1 on the surface of NK cells is not endogenously expressed but entirely derived from leukemic cells in a SLAM receptor-dependent manner. Further research has found that frequent trogocytosis (cell nibbling) between lymphocytes and tumor cells leads to NK cells acquiring PD-1 from leukemic cells, thus inhibiting NK cell-mediated tumor immune surveillance functions [119]. Trogocytosis refers to lymphocytes “gnawing off” part of the cell membrane from the antigen-presenting cells through the immune synapses, this part of the cell membrane usually carries the cell membrane surface molecules of donor cells, such as PD-1 [120]. This study provides a new perspective on the regulatory mechanisms of PD-1 expression in NK cells, we believe more similar discoveries will be made in the future.

In terms of clinical treatment, attention has begun to focus on NK cells. For example, Lin et al. evaluate the efficiency and safety of NK cell re-infusion combined with PD-1 antibody therapy in patients suffering from advanced non-small cell lung cancer. The results show that, compared to patients treated with Pembrolizumab monotherapy, patients receiving multiple NK cell re-infusions combined with Pembrolizumab have a prolonged overall survival of 18.5 months and an objective response rate (ORR) of up to 36.5%. Furthermore, tumor markers such as circulating tumor cells (CTC) significantly decrease after treatment, preventing cancer recurrence and metastasis [121]. Dong et al. find that PD-L1 expression on NK cells can be induced via the PI3K/AKT/NF-κB signaling pathway in human leukemia, increasing the cytotoxic function of NK cells. The use of anti-PD-L1 mAbs directly targets PD-L1^+^ NK (PD-L1-expressing NK) cells through p38 signaling to counteract PD-L1-tumors [122]. Therefore, the combined therapy of anti-PD-L1 mAb and PD-L1^+^ NK cells significantly improves the efficacy of cancer immunotherapies. This anti-tumor mechanism, independent of PD-1, provides new insights into the activation of NK cells and for the first time provides a potential explanation for why some patients lacking PD-L1 expression on tumor cells still respond to anti-PD-L1 monoclonal therapies. Additionally, most clinical trials indicate that NK cell immunotherapy does not significantly shrink tumors in advanced cancer patients but can serve as a postoperative adjuvant therapy to inhibit recurrence. In the future, we aim to truly achieve the precise killing of tumor cells through various immune combination therapies.

### PD-1/PD-L1 signaling affects cellular energy synthesis and metabolism

**Fig. 4:**
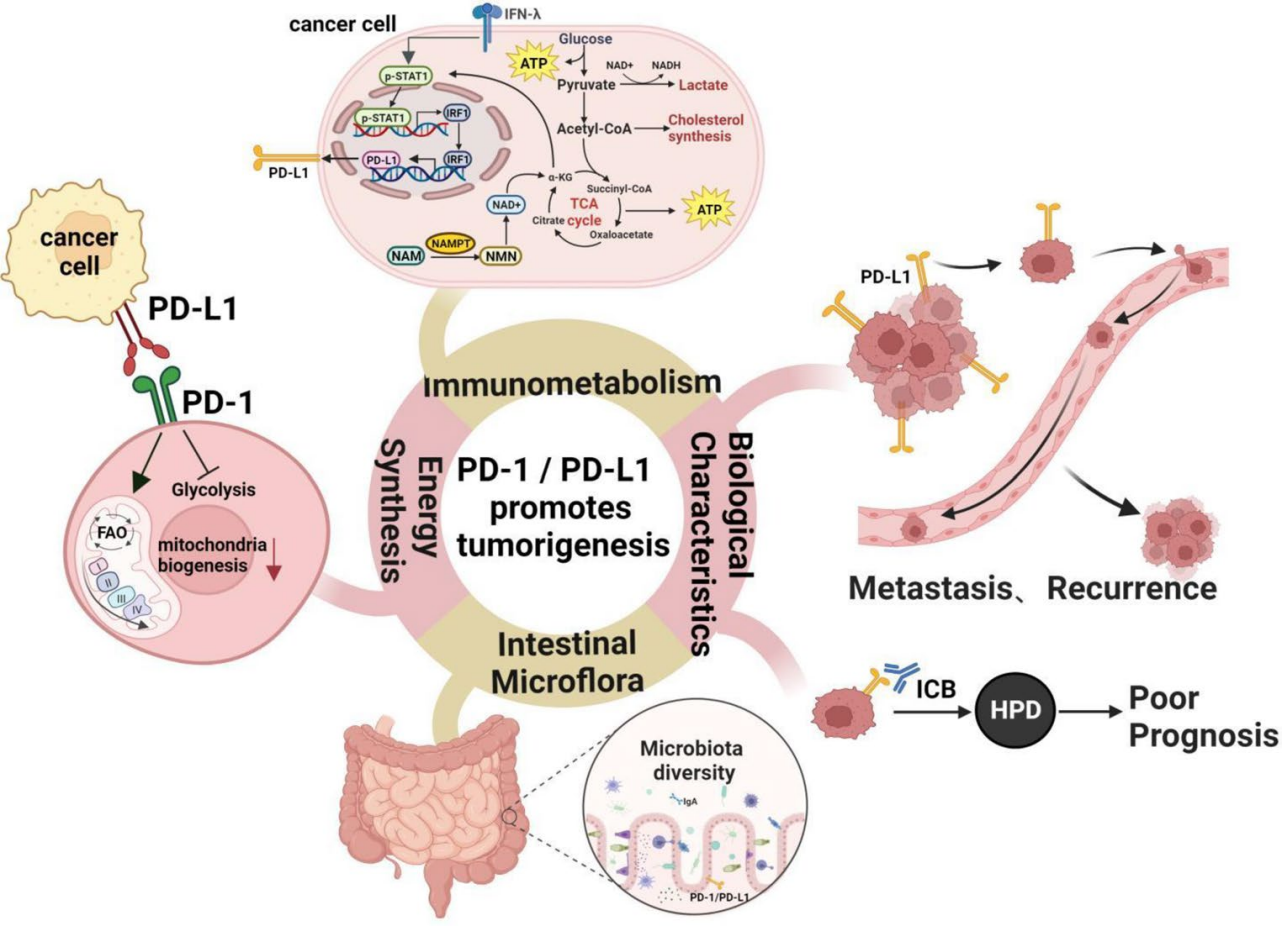
PD-1/PD-L1 promotes tumorigenesis and development. PD-1/PD-L1 signaling can alter cellular energy synthesis and metabolic pathways by disrupting aerobic glycolysis, thereby promoting fatty acid oxidation (FAO) as the main source of energy in T cells. Additionally, NAMPT, a component of NAD^+^ metabolism, can enhance PD-L1 expression in tumor cells through Stat1-dependent IFN-γ signaling, thereby inhibiting T cell function and remodeling the local tumor microenvironment, ultimately exerting significant effects on tumor metastasis, recurrence, and prognosis. Furthermore, the gut microbiota can also improve tumor progression and enhance T cell immune responses, suggesting its potential use in improving the efficacy of PD-1 blockade therapy. Figure created with BioRender

As tumor cells grow rapidly, some may die due to insufficient oxygen and nutrients. Under conditions where cell growth is rapid and surrounding nutrients are lacking, the anaerobic metabolism of glucose increases, leading to the accumulation of lactic acid. Lactic acid derived from tumors causes upregulation of PD-L1 in lung cancer cells and inhibits the function of T cells by disrupting aerobic glycolysis [123], thus creating an optimal environment for PD-1/PD-L1 interactions and tumor immune escape. Besides the supply of biological energy and biosynthesis, metabolic pathways such as glycolysis activation, increased lipid metabolism, and enhanced mitochondrial biosynthesis in TME also play a role in PD-1/PD-L1 signaling, reshaping the local tumor microenvironment and altering the metabolic adaptability of immune cells (Fig. 4). For example, PD-1 signaling stimulates the overexpression of the rate-limiting enzyme CPT1A in fatty acid oxidation (FAO), leading T cells to rely on FAO over glycolysis as their main source of energy [124]. This transition facilitates the transformation of effector T cells into memory T cells, FAO stimulates the production of more Treg cells, thus shaping an immunosuppressive microenvironment that promotes tumor escape. T cells require significant energy to combat cancer. Creatine is an important metabolic regulator controlling T cell anti-tumor immunity, it serves as a “molecular battery” to store bioenergy and provides power for T cell activity [125]. Insufficient intake of creatine severely impairs the anti-tumor immune responses of T cells, supplementing mice with creatine can significantly inhibit tumor growth. Moreover, the combination of creatine supplementation with PD-1/PD-L1 blockade therapy provides a continuous supply of energy to T cells, effectively preventing tumor progression for a long time and significantly improving T cell-based cancer immunotherapies.

In terms of immunometabolism, NAMPT (nicotinamide phosphoribosyltransferase) in NAD^+^ (nicotinamide adenine dinucleotide) metabolism increases PD-L1 expression in tumor cells through the Stat1-dependent IFN-γ signaling pathway, inducing CD8^+^ T cell exhaustion, thus resulting in tumor immune escape and poor prognosis for patients [126]. Further studies have shown that tumors with high expression of NAMPT are more sensitive to PD-1/PD-L1 blockade therapy. Supplementing with NAD^+^ precursors (β-nicotinamide mononucleotide, NMN) significantly increases sensitivity to immune therapy in tumors resistant to such treatments. This strategy of combining NAD^+^ supplementation with anti-PD-1/PD-L1 antibodies provides a novel therapeutic strategy for immune therapy-resistant tumors (Fig. 4). Additionally, Chao Zhong’s team [127] found that during DSS-induced colitis, the activation of intestinal lymphoid tissue inducer cells (LTi) is accompanied by increased PD-1 expression. Blocking the PD-1 signaling pathway leads to metabolic reprogramming of LTi cells, mainly manifested by reduced glycolysis, increased FAO, and decreased production of IL-22, eventually resulting in intestinal inflammation. Since excessive fatty acids promote FAO in LTi cells, obese patients may be more prone to intestinal complications caused by anti-PD-1 antibodies. Inhibiting FAO may have significant implications for intervening in intestinal inflammation. Qorraj et al. [128] find that the metabolic phenotype of monocytes is altered in chronic lymphocytic leukemia (CLL). Glucose uptake, glycolysis molecules, and glucose transporter expression decrease. Further research finds that the interaction between the PD-1 expressed on monocytes and the PD-L1 on CLL cells impairs glycolysis, phagocytosis, and BTK signaling, contributing to tumor immune evasion and weakening immune-based therapy. Conversely, disrupting PD-1/PD-L1 signaling reverses these immune metabolic dysfunctions and restores the anti-tumor activity of monocytes in CLL.

The gut microbiota environment is also an essential factor in regulating the immunity and sensitivity of PD-1 blockade therapies (Fig. 4). For example, Andrew’s team uses melanoma models and anti-PD-1/CTLA-4 therapy to demonstrate that immune checkpoint therapy induces the translocation of specific intestinal bacteria to secondary lymphoid organs and tumors, thus activating Dendritic Cells (DCs) and triggering anti-tumor responses of T cells. The use of antibiotics reduces the translocation of the gut microbiome to the mesenteric lymph nodes (MLN) and tumor-draining lymph nodes (TDLN), decreasing the responses of DCs and effector CD8 T cells [129]. This study suggests that the gut microbiome might also be a key mechanism in synergistically promoting extra-intestinal anti-cancer immunity and is critical for ICT treatment. Matson et al. find that the symbiotic microbiome improves tumor control, enhances T cell responses, and increases the effectiveness of anti-PD-L1 therapy in patients with metastatic melanoma, so maintaining a healthy gut microbiota helps patients fight against cancer [130]. Gopalakrishnan et al. investigate the oral and intestinal microbiota of 112 patients receiving anti-PD-1 immunotherapies, where 30 “responsive” patients contain a higher abundance of Ruminococcaceae family bacteria in their feces and a more abundant synthetic metabolic pathway in their bodies, resulting in stronger systemic immunity and anti-tumor immunity [131]. Zhao’s team selected 65 patients with advanced hepatobiliary cancer undergoing anti-PD-1 therapies and divided them into the clinical benefit response (CBR) group and the non-clinical benefit (NCB) group. Bioinformatics analysis discovers that gut microorganisms might influence immune therapy responses by mediating metabolic pathways. The enriched microbiome in the CBR group is related to energy metabolism, significantly prolonging the OS of patients undergoing immunotherapy. Whereas the enriched microbiome in the NCB group is related to amino acid metabolism, with patients having a shorter OS [132]. In conclusion, these findings have tremendous implications for cancer immunotherapy, suggesting that modulating the gut microbiota and metabolism improves the effectiveness of PD-1 blockade therapy. Metabolic pathways involving different gut microbes may represent potential important mechanisms affecting the effects and survival benefits of anti-PD-1 immunotherapy in hepatobiliary tumors, serving as efficacious biomarkers for predicting immunotherapy. Perhaps one day, supplementing the body with specific gut bacteria beneficial for the action of PD-1 antibodies or undergoing specific fecal transplantation, can increase the effectiveness of immunotherapy.

### Effects of PD-1/PD-L1 signaling on tumor metastasis, recurrence, and prognosis

PD-1/PD-L1 is involved not only in the regulation of tumor immunity but also plays a certain role in tumor metastasis and recurrence. For example, PD-1 or PD-L1 promotes the malignant growth and autophagy of tumors such as ovarian cancer and melanoma by activating the mTORC signaling pathway [133, 134]. PD-L1 is lowly expressed in poorly differentiated and metastatic GC cells, knocking down PD-L1 reduces the expression of E-cadherin, promotes cell migration and wound healing capabilities, and prolongs the survival time of patients [135]. PD-L1 also promotes the growth and metastasis of cervical cancer by activating the ITGB4/SNAI1/SIRT3 signaling pathway. Specifically, PD-L1 activates the AKT/GSK3β signaling pathway by directly binding to integrin β4 (ITGB4), thereby inducing the expression of the transcriptional repressor SNAI1. SNAI1, in turn, affects the gene expression involved in EMT and regulates glucose metabolism by inhibiting the activity of the SIRT3 promoter [136]. Therefore, overexpression of PD-L1 significantly increases tumor glucose uptake, promotes lymph node metastasis, and is significantly associated with poor prognosis in patients, targeting PD-L1 and its downstream effector molecules as a potential approach to interfere with cervical cancer growth and metastasis. In addition, CD8^+^ T cells induce PD-L1 expression on hepatocellular carcinoma cells in an IFN-γ-dependent manner, which in turn promotes the apoptosis of CD8^+^ T cells, leading to disease progression and postoperative recurrence, blocking PD-L1 reverses this phenomenon [137].

High PD-L1 expression makes tumor cells more sensitive to PD-1/PD-L1 inhibitors, using PD-1 or PD-L1 antibody therapy can stimulate immune cells to “recognize” cancer cells again, thus enhancing their own immunity to attack cancer cells and significantly prolonging patient survival. For example, results from first-line clinical trials in advanced NSCLC patients show that compared to platinum-based chemotherapy, treatment with Pembrolizumab provides PD-L1^+^ patients with longer progression-free survival and overall survival, as well as fewer immune-related adverse events [138]. However, some patients with advanced malignancies may be at risk of experiencing hyper-progressive disease after receiving ICI therapy. Kim et al. evaluated 263 patients with recurrent and metastatic NSCLC receiving PD-1/PD-L1 inhibitors. The results show that PD-1/PD-L1 blockade leads to the occurrence of HPD in many NSCLC patients. The frequency of severely exhausted CD8 T cells (TIGIT^+^ PD-1^+^ CD8 T cells) in the peripheral blood of HPD patients is higher than that of non-HPD patients, and the frequency of effector/memory subsets (CCR7^−^ CD45RA^−^) is lower, with worse PFS and OS [139]. Li et al. find that specific carcinogenic pathways promote the development of HPD following PD-1/PD-L1 blockade therapy. ICI-activated cytotoxic CD8^+^ T cells released IFN-γ, promoting tumor FGF2 signaling, thereby inhibiting PKM2 activity and reducing NAD levels, enhancing SIRT1-mediated acetylation of β-catenin, ultimately activating the Wnt-β-catenin pathway and leading to HPD [140] (Fig. 4).

The above studies suggest that the overexpression of PD-1/PD-L1 in cancer can promote the occurrence of malignant tumor behavior through multiple signaling pathways. Although targeting PD-L1-mediated extracellular signaling pathway brings effective anti-cancer therapies, some patients have experienced rapid cancer progression during ICI immunotherapy, making it difficult for patients to benefit from clinical treatment. It can be seen that the regulatory roles of the PD-1/PD-L1 pathway in tumor initiation and progression are complex and varied, possibly related to specific genetic backgrounds, tumor biology behavior, or post-translational modifications specific to PD-L1 or PD-1. In the future, exploring and identifying biomarkers that can predict the risk of HPD in response to ICI treatment, preventing the occurrence of HPD by exhausting CD8^+^ T cells, and overcoming tumor immune resistance to prevent tumor metastasis and recurrence, holds significant clinical significance.

### The effect of PD-1/PD-L1 signal on the tumor itself

Traditionally, most studies have emphasized the molecular interactions and modifications between PD-1 and PD-L1 to seek novel tumor immunotherapy approaches. Recent research has revealed that PD-L1 exhibits extensive intracellular and extracellular distribution. It can be expressed in various forms in tumor cells, such as cytoplasmic PD-L1 (cPD-L1), membrane PD-L1 (mPD-L1), soluble PD-L1 (sPD-L1), and extracellular vesicular PD-L1 (EV PD-L1) [141]. Currently, only the function of mPD-L1 has been widely recognized and confirmed. mPD-L1 inhibits T cell function by binding to PD-1 on T cells, and its high expression is often related to tumor progression and poor prognosis in patients [142]. The distribution and immune regulatory function of non-cellular membrane PD-L1 have not been fully demonstrated. Recent studies have suggested that besides its role in promoting tumor cell escape from immune surveillance, PD-L1 is also considered a crucial effector molecule that transduces intrinsic signals in tumor cells to prevent their own apoptosis or to facilitate tumor progression in an immune-independent manner.

**Fig. 5:**
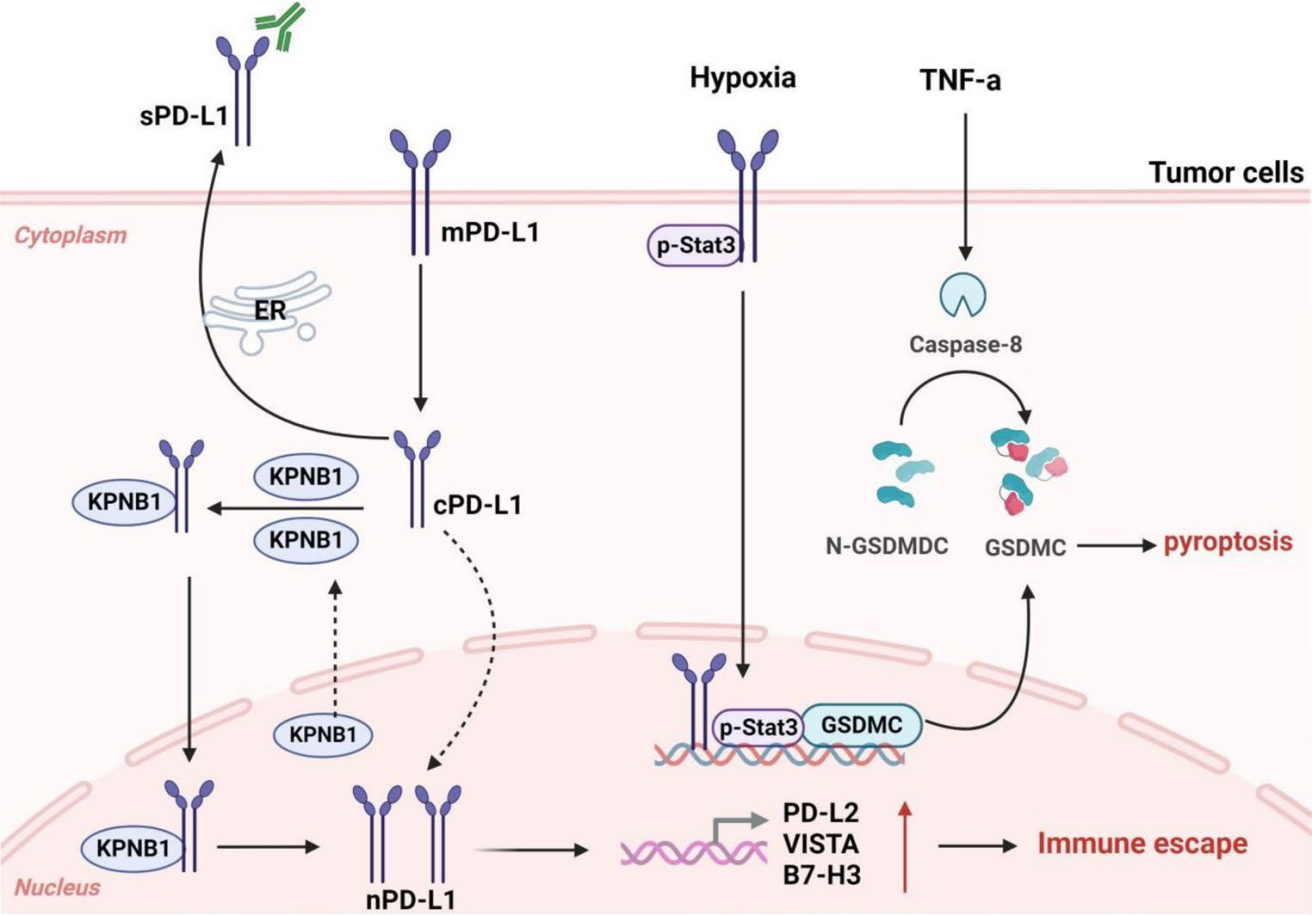
The effects of nuclear PD-L1 on the tumor itself. PD-L1 not only promotes tumor cells to evade immune surveillance but also facilitates tumor progression in an immune-independent manner. PD-L1, which is expressed at high levels in tumor cells, can enter the nucleus by binding to the nuclear entry protein KPNB1 and exert pro-cancer effects. Nuclear PD-L1 can also trigger the upregulation of immune checkpoint genes, including PD-L2 and VISTA, thereby enhancing the anti-tumor responses of PD-1 inhibition. Under hypoxic conditions, treatment with TNFα and CHX can promote the nuclear translocation of PD-L1, which then interacts with p-Stat3-Y705. Subsequently, p-Stat3-Y705 combines with the GSDMC promoter region, leading to the upregulation of GSDMC gene expression. Furthermore, GSDMC is cleaved and activated by caspase-8, triggering pyroptosis in cells and necrosis in the hypoxic areas of tumors. Figure created with BioRender

As early as 2010, Ghebeh et al. reported that chemotherapy induces nuclear translocation of PD-L1 in breast cancer cells, suggesting that PD-L1 has functions beyond inhibiting T cells [143]. Subsequently, the Shulin Li team found that circulating tumor cells (CTCs) from patients with prostate and colorectal cancers have higher expressions of nPD-L1, indicating that nuclear localization of PD-L1 may be involved in cancer progression and metastasis, holding a significant prognostic value [144]. In TNBC, nuclear PD-L1 is important for regulating the cohesion of sister chromatids and the genomic stability of cancer cells. PD-L1 deficiency leads to the formation of multinucleated cells and the separation of sister chromatids, thereby promoting tumor growth [145]. Du et al. find that in non-small cell lung cancer, highly expressed PD-L1 enters the nucleus by binding to the nucleoprotein KPNB1, then activates the Gas6/MerTK and its downstream AKT and Erk signaling pathways, thereby promoting tumor cell proliferation [146], offering a novel perspective for the optimization of lung cancer immunotherapy. Gao et al. find that under hypoxic conditions, treatment with TNFα and CHX promotes the nuclear translocation of PD-L1, where it interacts with phosphorylated (p)-Stat3-Y705. Subsequently, p-Stat3-Y705 binds to the gasdermin C (GSDMC) promoter region, upregulating GSDMC gene expression, and then caspase-8 cleavage and activation of GSDMC, inducing pyroptosis in cells and resulting in necrosis in the hypoxic region of tumors [147]. Moreover, nuclear PD-L1 also induces tumor cells to express other immune checkpoint molecules, including PD-L2 and VISTA, thus enhancing the anti-tumor responses to PD-1 blockade [148]. Since these molecules are not the targets of the PD-1/PD-L1 blockade, they may contribute to acquired resistance to immune therapy. In bladder cancer, intrinsic PD-L1 can also activate intrinsic cancer cell signals in a PD-1 independent manner and enhance cancer cell proliferation and survival by activating mTOR and inhibiting autophagy [149]. Therefore, the non-immune-dependent functions of nuclear PD-L1, such as participating in tumor pro-inflammatory pathways and reprogramming the expression of genes related to immune responses, may be significant for immunotherapy (Fig. 5).

Not only that, the inherent PD-1 in tumor cells can act as a potential tumor suppressor to inhibit tumor growth. For instance, Wang’s team found that not only PD-L1 is highly expressed in various tumor cells, including lung and liver cancer, but PD-1 is also widely expressed. PD-1 reduces tumor cell proliferation and colony formation by inhibiting the classical AKT and ERK1/2 signaling pathways. Similarly, Shisuo Du et al. also find high expression of inherent PD-1 in tumor cells of NSCLC patients. Treatment with PD-1 targeted antibodies enhances the survival ability of lung cancer cells in vitro, and even potentially develops resistance to PD-1 inhibitor therapy [150]. This also provides some evidence for why some patients do not respond effectively to PD-1 antibody treatment. In contrast, Kleffel et al. detect PD-1 expression on the surface of melanoma cells. The inherent PD-1 binds to its ligand PD-L1 and activates the downstream mTOR signaling through PI3K/AKT-independent pathway, thereby promoting tumor cell self-proliferation, even in mice lacking adaptive immunity. Additionally, PD-L1 expressed in melanoma also promotes tumor growth through paracrine or autocrine interaction with PD-1 [134]. Gupta et al. discover that tumor-initiating cells (TICs) highly expressed PD-L1 in ovarian cancer and melanoma, the intrinsic cell signals produced by PD-L1 promote TIC proliferation and mTORC1 activation, and PD-L1 expression also increases the sensitivity of TIC to IFN-γ and rapamycin, thereby resisting tumor immunotherapy and promoting tumor recurrence [151]. So does PD-1 expressed in tumors play a role in promoting or inhibiting cancer? We speculate this may be context-dependent, as different types of tumor cells or selective signaling pathways may mediate different regulatory functions in tumor cells. More studies are still needed to explain the distinct roles of PD-1 signaling in different cancer types, as well as the specific molecular and cellular mechanisms involved in its regulation. In conclusion, these studies suggest that atypical local PD-1 and PD-L1 may also serve as new targets for cancer therapy, offering new insights for devising optimal ICI treatment strategies to benefit cancer patients.

### The regulatory mechanisms of PD-1/PD-L1 expression in cancers

The activation of PD-1/PD-L1 signaling is not only associated with maintaining autoimmune homeostasis and establishing peripheral immune tolerance, but also involved in tumor progression and immune evasion, serving as a crucial inhibitor of both innate immune and adaptive immune systems [152]. However, the underlying mechanisms regulating the expression of the PD1/PD-L1 pathway are not fully understood. We speculate that specific signals transmitted by cells or inflammatory factors in the tumor may play a dominant role in the regulation of PD-1/PD-L1 expression. Therefore, we summarize the previous regulatory mechanisms of PD-1/PD-L1 expression in tumors, and on this basis, look forward to the application prospects of tumor immunotherapy targeting the PD-1/PD-L1 axis, which could contribute to exploring new therapies for tumor immunity.

### Regulation of PD-1 expression in tumor immunity

PD-1 is a major immune checkpoint that regulates T cell activation and ensures peripheral tolerance. It is typically highly expressed in activated T and B lymphocytes, NK cells, and tumor-infiltrating lymphocytes. PD-1 expression is a complex and dynamic regulatory process influenced by various factors, including antigen signaling stimulation, inflammatory factors, and cell types within TME.

During the initial immune phase, T cells, under the stimulation of the MHC-TCR signaling pathway, enhance the enhancer activity within the major transcription regulatory regions (CR-B and CR-C) of PD-1 through reversible demethylation actions [153], and activate the initial transcription of PD-1 under the synergistic actions of various transcription activators such as NF-κB [154], NFATc1 [155], STAT3/4 [156]. When acute virus infection occurs, TCR signaling gradually diminishes as the antigen is cleared, the methylation level of PD-1 enhancers gradually recovers, weakening their transcriptional activation and strengthening their inhibitory effects [157–159]. Thus, PD-1 is not expressed on resting memory T cells during acute infection. Conversely, under chronic stimulation and persistent antigen infection, cytotoxic T lymphocytes may become dysfunctional, with impaired secretion of cytokines including TNF-α, IFN-γ, and IL-2, leading to a significant increase in PD-1 expression on T cells. Continuously high expression of PD-1 will transmit inhibitory signals, inducing immune tolerance. For example, upregulation of PD-1 expression in melanoma promotes tumor evasion from immune surveillance, protecting cancer cell growth [134]. At this time, the regulation of PD-1 expression occurs as follows, sustained TCR signaling leads to irreversible demethylation of PD-1 enhancers, relieving the restriction of TCR signaling and driving PD-1 into a state of “uncontrolled” transcriptional activation [160, 161], which further exacerbates T cell functional exhaustion and results in severe immune suppression.

Moreover, other signaling pathways and factors are still involved in the subsequent expression regulation of PD-1 due to the influence of the tumor microenvironment and abnormal cytokine secretion. For example, the transcriptional activator FoxO1 enhances PD-1 expression by binding to the corresponding element within CR-C of PD-1, thereby activating inhibitory signaling pathways, leading to impaired downstream PI3K/AKT/mTOR signaling, so that FoxO1 accumulates in the nucleus and forms a negative feedback regulatory mechanism to enhance the expression of PD-1 [162]. IFN-α activates the JAK/STATs pathway, forming an ISGF3 complex composed of STAT1-STAT2 heterodimers and interferon response factor 9 (IRF9), the complex binds to the interferon-sensitive response element within CR-C to enhance PD-1 expression [163]. Type I IFN also induces the secretion of IL-7 to suppress PD-1 expression in acute viral hepatitis, maintaining antiviral CD8^+^ T cell response and internal balance in vivo [164]. The inflammatory factor TGF-β1 upregulates PD-1 and CTLA-4 expression in T lymphocytes through the calcium-regulated phosphatase nuclear factor of activated T cells 1 (CaN/NFATc1) pathway, thereby inducing immune escape [165]. More signaling pathways associated with PD-1 expression regulation await further research.

Increasing research has discovered that tumors maintain a state of T cell exhaustion through PD1 signaling, blocking the PD-1 signaling pathway restores or reverses the activity and immune function of exhausted T cells to some extent, enhancing the effectiveness of cancer immunotherapy [77, 166, 167]. Currently, PD-1 blockade therapy has been applied in the immunotherapy of various advanced cancers including melanoma [168], non-small cell lung cancer [169], and colorectal cancer [170].

### Regulatory mechanisms of PD-L1 expression in cancers

The impact of PD-L1 in tumors is not fully understood and represents a dynamic and variable process. Numerous reports have indicated that PD-L1 expression is regulated by different intrinsic and extrinsic mechanisms, including genomic alterations, post-transcriptional regulations, post-translational modifications of proteins, carcinogenic signals, inflammatory cytokines, etc. Increased PD-L1 expression in TME restricts host immune responses and induces the development of an immunosuppressive microenvironment in cancer [171, 172]. Therefore, PD-L1 is widely regarded as a crucial immune checkpoint associated with cancer progression and poor patient prognosis, and it holds broad clinical application prospects as a predictive biomarker and therapeutic target in the field of oncology.

### Regulatory mechanisms of PD-L1 expression at the gene level

The PD-L1 gene is located on chromosome 9p24.1. Research has shown that PD-L1 gene amplification and structural variations (SVs), chromosomal aberrations, and chromosomal diversity can all cause the upregulation of PD-L1 expression in tumors, making it a promising biomarker for tumor treatment. Copy number variations (CNVs) of the PD-L1 gene affect PD-L1 gene expression levels. In nodular sclerosing Hodgkin’s lymphoma, amplification of the 9p24.1 chromosome is directly related to increased PD-L1 expression on Reed-Sternberg cells [173]. Tumors with an amplified PD-L1 gene often have a higher mutational burden, closely associated with increased PD-L1 expression in malignant cells and poor patient outcomes [174, 175]. However, PD-L1 gene amplification is not always associated with high PD-L1 positivity [176]. PD-L1 amplification occurs only in a small subset of malignancies, such as oral squamous cell carcinoma [177], non-small cell lung cancer [178], cervical squamous cell carcinoma [179], and triple-negative breast cancer [180]. Additionally, in the classical Hodgkin lymphoma and the primary mediastinal large B-cell lymphoma, the amplified region of the 9p24.1 chromosome also includes the Janus kinase 2 (JAK2) locus. JAK2 amplification can enhance PD-L1 expression by activating the IFN-γ pathway and increase sensitivity to JAK2 inhibition [181, 182]. Using the JAK2 inhibitor TG-101348 significantly reduces PD-L1 protein expression levels. The amplification of PD-L1 is caused by genomic rearrangements, ultimately resulting in high PD-L1 expression. Chong LC et al. comprehensively characterized 36 novel PD-L1 structural rearrangements (SRs) in B-cell non-Hodgkin’s lymphoma, including intrachromosomal inversions, duplications, deletions, and translocations of locus fragments, and found that PD-L1 SRs are significantly correlated with PD-L1 protein expression [183]. PD-L1 chromosomal translocations also lead to PD-L1 overexpression to promote tumorigenesis in some diffuse large B-cell lymphomas [184]. In addition, some non-CNV changes in the PD-L1 gene, such as deletion of PD-L1 exons, SNP site mutation [185], DNA double strand break [186], and PD-L1 3’ UTR structural variation [187], are emerging as new genetic mechanisms regulating PD-L1 gene expression.

### Regulatory mechanisms of PD-L1 expression at the epigenomic level

Apart from the gene level, epigenetic modifications (DNA methylation, histone methylation, histone acetylation, etc.) in the PD-L1 promoter are also involved in the regulation of PD-L1 expression. Aberrant promoter methylation and histone modifications of PD-L1 may be potential mechanisms leading to its upregulation.

PD-L1 promoter methylation relates to PD-L1 expression, methylated DNA downregulates PD-L1 expression by suppressing its gene transcription. Methylation of the PD-L1 promoter has been found to be negatively correlated with its mRNA and protein expression in various cancers such as gastric cancer [188], diffuse low-grade glioma [189], and non-small cell lung cancer [190]. Hypomethylation of the PD-L1 promoter typically results in upregulated PD-L1 expression, thus exerting immune inhibitory effects [191]. DNMT (DNA methyltransferase) inhibitors induce PD-L1 protein expression and improve the response to PD-L1 blockade therapy in tumors [192]. In the EMT pathway of NSCLC, TNF-α/TGFβ1 induces demethylation of the PD-L1 promoter by reducing DNMT1 levels, which leads to upregulation of PD-L1 expression [193]. Low-dose decitabine (a DNA demethylation drug) combined with PD-1 inhibitors can promote the expansion and effector function of CD8^+^ exhausted precursor T cells by maintaining the activity of the AP-1/JunD pathway, thus achieving more potent and durable anti-tumor activity. The combination synergistically improves immune surveillance efficacy against tumors [194]. Data from a phase II clinical trial in relapsed or refractory Hodgkin lymphoma shows that, compared to monotherapy with camrelizumab, patients treated with Decitabine and camrelizumab (anti-PD-1 monoclonal antibody) combination therapy have an increased percentage of circulating peripheral central memory T cells, an overall remission rate (ORR) of up to 95%, and significantly prolong progression-free survival (PFS) [195]. Therefore, combining demethylation inhibitors with anti-PD-L1 antibodies may be a more effective oncology treatment strategy.

Acetylation of the histones in the PD-L1 gene promoter region can induce PD-L1 expression. HDAC primarily participates in regulating acetylation by removing acetyl groups from the N-acetylated lysine residues of histones [196]. In breast cancer, HDAC1/2 is recruited to the PD-L1 promoter by the TET2 protein, leading to deacetylation of H3K27ac, thus inhibiting PD-L1 transcription [197]. Treatment with HDAC inhibitors mainly upregulates histone acetylation on the upstream bases of the PD-L1 exons, causing chromatin relaxation and increasing the activity of the PD-L1 promoter fragment, thus enhancing PD-L1 gene expression [198]. In non-small cell lung cancer, hepatocellular carcinoma, and breast cancer, the reduction of COP1 (E3 ligase constitutive photomorphogenesis protein 1) increases c-Jun accumulation and subsequently inhibits the expression of histone deacetylase 3 (HDAC3), thereby enhancing histone H3 acetylation and promoting PD-L1 transcription [199]. Furthermore, BRD4 (bromodomain-containing protein 4), a member of the BET (bromodomain and extra-terminal domain) family, plays a crucial role in chromatin remodeling and transcriptional activation. BRD4 effectively enhances the PD-L1 gene transcription by RNA polymerase II through binding to acetylated histone H3K27Ac in the PD-L1 gene promoter region and distal enhancers [200]. In pancreatic cancer, histone acetyltransferase 1 promotes PD-L1 gene transcription by facilitating the binding of a complex containing BRD4 to acetylated histone H4 [201]. Romidepsin (selective inhibitors of HDAC1 and 2) mainly enhances PD-L1 expression in colorectal cancer by modulating histone H3 and H4 acetylation [202]. HDAC3 inhibitors quickly increase the recruitment of BRD4 to the PD-L1 gene promoter region, thus activating PD-L1 transcription [203].

In pancreatic cancer, the histone methyltransferase MLL1 can enrich H3K4me3 on the PD-L1 promoter, promoting the expression of PD-L1. Inhibiting MLL1 significantly reduces H3K4me3 enrichment in the PD-L1 promoter region [189]. Conversely, in liver cancer, the histone methyltransferase EZH2 acts as an intrinsic modifier to negatively regulate IFN-γ-induced PD-L1 expression. EZH2 suppresses PD-L1 expression by directly upregulating H3K27me3 in the promoters of PD-L1 and IRF1, without affecting the activation of the IFNγ-STAT1 signaling pathway [204]. In breast cancer, Histone Lysine-specific Demethylase 1 (LSD1) inhibitors increase the enrichment of H3K4me2 at the proximal element or core regions of the transcription start site on the PD-L1 promoter, increasing PD-L1 expression in TNBC cells. The combination of an LSD1 inhibitor and PD-1 mAb synergistically enhances tumor immunogenicity and the anti-tumor efficacy of immunotherapy [205]. In some HCC cells, Lysine-specific demethylase 1 A decreases MEF2D methylation by interacting with it, demethylated MEF2D binds to the PD-L1 promoter and activates PD-L1 expression, thus promoting immune inhibitory activity [206].

### Mechanisms of PD-L1 expression regulation in transcriptional and post-transcriptional

#### Transcriptional regulation

##### Transcription factors involved in PD-L1 regulation

The regulation of PD-L1 expression mainly involves the participation of transcriptional regulators, upstream and downstream signaling molecules and receptors. At present, multiple transcriptional regulators involved in regulating PD-L1 expression have been identified.

#### MYC

Overexpression of MYC is observed in approximately 70% of tumorigenesis. The transcription factor MYC can directly regulate PD-L1 transcriptional initiation through binding to the PD-L1 promoter and enhance PD-L1 expression. Inactivation of MYC has been found to downregulate PD-L1 mRNA and protein expression, enhancing the anti-tumor immune responses in tumor tissues such as hepatocellular carcinoma, acute T-lymphocytic leukemia, and melanoma [207]. In esophageal squamous cell carcinoma, ChIP sequencing results show that C-Myc upregulates the expression of PD-L1 through binding to the PD-L1 promoter, thereby promoting tumor immune escape and resulting in poorer overall survival in patients [208]. Treatment with c-Myc inhibitors significantly inhibits PD-L1 transcription, such as in lung cancer, where Bafetinib (a tyrosine kinase inhibitor) can reduce PD-L1 transcription by inhibiting the transcription of c-Myc [209].

#### CDK

The cyclin-dependent kinase (CDK) family also plays a major role in PD-L1 regulation. For instance, the transcription factor TFIIH is important in transcription and DNA repair, its CDK7 subunit can regulate the transcription of PD-L1 in a MYC-dependent manner and promote tumor progression. In NSCLC cells, the CDK7 inhibitor THZ1 inhibits MYC transcriptional activity by downregulating p38α, thereby suppressing PD-L1 expression and improving the anti-tumor immunity against PD-1 therapy in vivo by recruiting infiltrating CD8^+^ T cells [210]. Similarly, the expression levels of cyclin-dependent kinase 5 (CDK5) and PD-L1 are positively correlated in a variety of tumors. IFN-γ-induced upregulation of PD-L1 requires Cdk5. The absence of Cdk5 induces the expression of PD-L1 transcriptional inhibitors (IRF2 and IRF2BP2), maintaining transcriptional repression of PD-L1 after IFN-γ stimulation, resulting in increased infiltration of CD4 T cells and promoting anti-tumor immune responses [211].

#### STAT

After binding to its receptor, IFN-γ activates the downstream JAK-STAT signaling pathway, mainly through STAT1 to stimulate the expression of downstream transcriptional regulators. The induction of IRF1 by STAT acts as the major transcriptional regulator of PD-L1 expression induced by IFN-γ. Garcia-Diaz et al. have identified two IRF1 binding sites in the PD-L1 promoter region, the abolition of the IRF1 site leads to a reduction in PD-L1 levels [212]. TET2 is an important factor in the IFN-γ/JAK/STAT1 pathway, it can bind to STAT1 activated by IFN-γ, catalyze the hydroxylation of 5mC, and activate the expression of Th1-type chemokines and PD-L1 [213]. HDAC2 (histone deacetylase 2) enhances PD-L1 expression by increasing the phosphorylation of JAKs/STAT1 and recruiting STAT1 to the PD-L1 promoter. Simultaneously, HDAC2 is also recruited to the PD-L1 promoter by STAT1. Knockout of HDAC2 impairs the IFN-γ-induced H3K27 upregulation, H3K9 acetylation, and recruitment of BRD4 to the PD-L1 promoter region, inhibiting the immune escape and metastasis of triple-negative breast cancer (TNBC) [214]. Chidamide (a class I HDAC inhibitor) upregulates the expression of PD-L1 by activating the transcription factor STAT1 in chondrosarcoma [215]. STAT3 is another important oncogenic transcription factor that directly binds to the PD-L1 promoter to regulate PD-L1 transcription [216]. It also inhibits the elevated PD-L1 protein expression caused by NPM/ALK (nucleophosmin/anaplastic lymphoma kinase) mutations by silencing STAT3 [217]. Additionally, HDAC3 upregulates PD-L1 expression through the STAT3 pathway in pancreatic cancer. Using an HDAC3 inhibitor or knocking down HDAC3 weakens the binding of STAT3 to the PD-L1 promoter, thereby destroying the HDAC3/STAT3/PD-L1 signal transduction and improving the immunotherapy efficacy in pancreatic cancer [218]. Similarly, in melanoma, HDAC8 participates in the transcriptional regulation of PD-L1 by interacting with the transcriptional complex of HOXA5 and STAT3, thereby inhibiting PD-L1 expression [219].

#### NF-κB

PD-L1 expression in cancer cells also depends on the transcription factor NF-κB. Both the promoter region of the PD-L1 gene and the enhancer region 140 Kb downstream of the PD-L1 gene contain NF-κB binding sites, the NF-κB subunit RelA can bind to the PD-L1 promoter and directly participate in the regulation of PD-L1 expression [220]. NF-κB is activated by various oncogene mutations and inflammatory signaling pathways. Among which, TNF-α and INF-γ induce PD-L1 expression through the NF-κB pathway in breast cancer, prostate cancer, and colon cancer. In particular, TNF-α-dependent activation of IKKε induces the recruitment of p65 to the PD-L1 promoter region, thereby upregulating PD-L1 expression [193]. Ultraviolet radiation (UVR) promotes the immune escape of cancerous melanocytes and the development of melanoma by upregulating NF-κB and IRF3-dependent PD-L1 transcription [221]. There are reports that NF-κB inhibitors can significantly suppress the expression of PD-L1 [222].

#### Other oncogenic transcription factors

AP-1, a transcriptional regulator composed of FOS, c-Jun, MAF, and ATF subunits, binds to the enhancer region of the PD-L1 gene located about 5 kb downstream of the transcription start site to participate in the transcriptional regulation of PD-L1, promoting its expression in Hodgkin’s lymphoma [223]. Hypoxic conditions induce the activation of hypoxia-inducible factor-1α (HIF-1α) and the accumulation of lactic acid, with both HIF-1α and HIF-2α being able to directly bind to the hypoxia response element in the PD-L1 promoter region, participating in the transcriptional regulation of PD-L1 [224]. The transcriptional activator NRF2, upon stimulation by IFN-γ, directly binds to the PD-L1 promoter and induces the transcription of PD-L1, mediating its upregulation [225]. The endogenous transcriptional regulator nucleophosmin 1 specifically binds to the PD-L1 promoter and activates the PD-L1 transcription in triple-negative breast cancer cells, thereby increasing PD-L1 mRNA and protein expression [226].

#### The carcinogenic pathway participates in the transcriptional regulation of PD-L1

Several oncogenic signaling pathways, such as Akt/mTOR, JAK/STAT3, and EGFR/MAPK, or the loss or silencing of PTEN, ALK, and EGFR, are involved in the regulation of PD-L1 transcription, potentially leading to the upregulation of PD-L1 expression in tumor cells [227]. IFN-γ released by T cells, is a crucial regulator inducing PD-L1 expression in tumor cells, where the IFN-γ/JAKs/STATs/IRF1 axis is the main pathway to induce and maintain PD-L1 expression in malignancies. The binding of IFN-γ to its receptor leads to the phosphorylation of tyrosine on JAK1 and JAK2, initiating the phosphorylation of STAT1/STAT3 proteins [228]. STAT1 then translocates to the nucleus, binds as a transcription factor to genes that possess gamma interferon activation sites (GAS) to induce the transcription of IFN-stimulated genes (ISG), including IRF1 and IRF9. IRF1 binds to the PD-L1 promoter, significantly enhances PD-L1 expression [229, 230]. IFN-γ also directly upregulates the expression of PD-L1 in tumor and immune cells in TME by activating NF-κB and PKD2 signaling pathways [231]. Phosphorylation of JAK has also been shown to activate other important pathways, including PI3K, RAS, AKT, and MAPK [232]. Loss-of-function mutations in the JAK1/JAK2 pathway result in the absence of PD-L1, which triggers primary and acquired resistance to PD-1 blockade therapy. PD-L1 transcription is also regulated by PTEN/PI3K/AKT/mTOR signaling, where the loss of PTEN or the activation of mTOR can promote PD-L1 expression, high PD-L1 expression also in turn promotes the loss of PTEN expression in cells, suggesting a reciprocal interaction between the two [233, 234]. Some lymphocyte chemokines, such as CXCR2 and CXCL5, increase PD-L1 expression by activating the PI3K/Akt/GSK-3β/Snail signaling pathway, leading to epithelial-mesenchymal transition and promoting cancer invasion and metastasis [235].

**Fig. 6:**
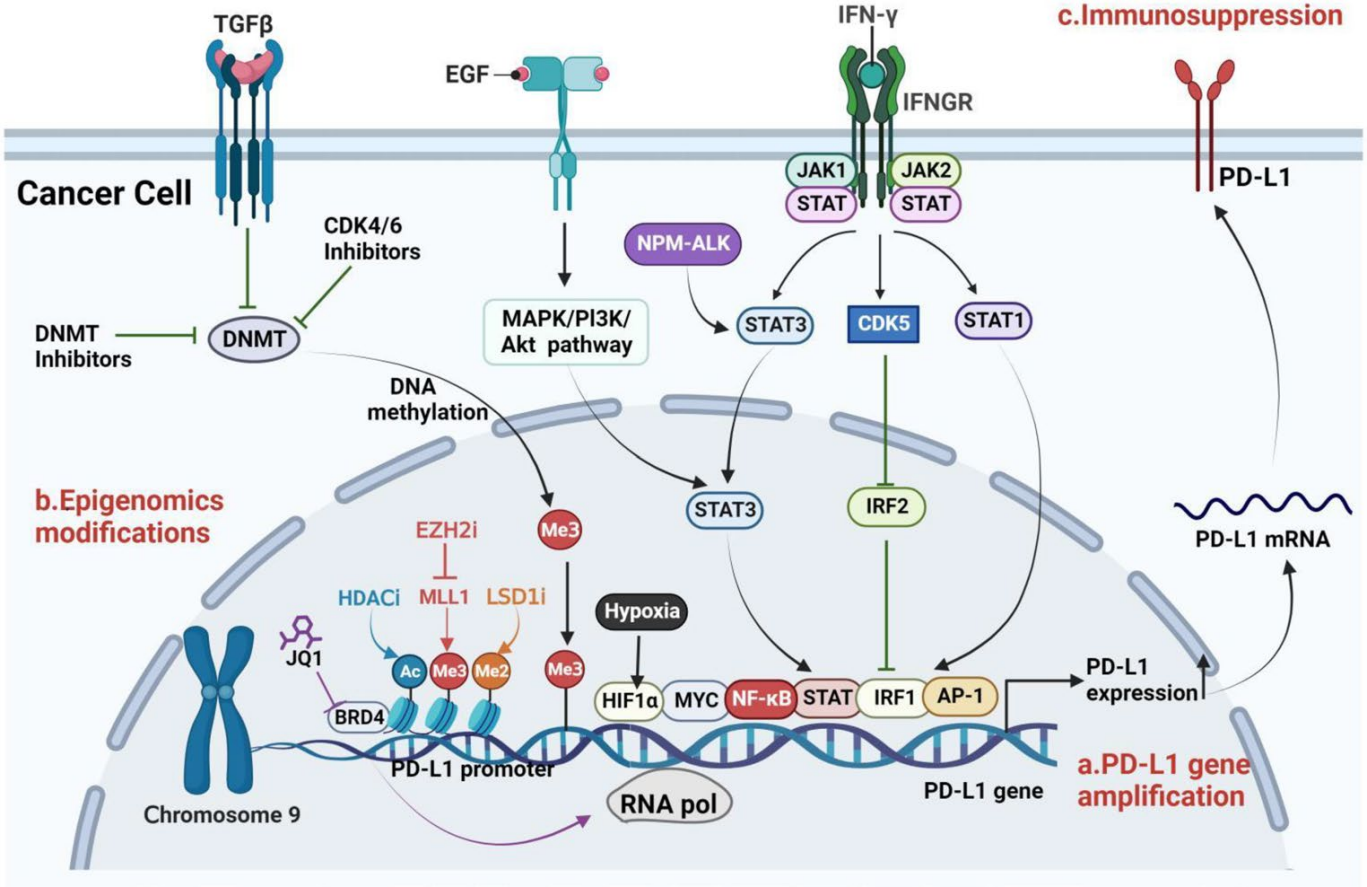
Genetic and transcriptional regulatory mechanisms of PD-L1 expression. At the gene level, tumors with PD-L1 gene amplification in chromosome 9p24.1 have a higher mutation load and a close correlation with increased PD-L1 expression. Epigenetic modifications of the PD-L1 promoter region, including DNA methylation, histone methylation, and acetylation, are also important in the regulation of PD-L1 expression. For example, TNF-α/TGFβ1 induces demethylation of the PD-L1 promoter by decreasing DNMT1 (DNA methyltransferase) levels, resulting in PD-L1 up-regulation and thus exerting immunosuppressive effects. At the transcriptional level, PD-L1 expression is primarily regulated by transcription factors, including STAT, MYC, NF-κB, IRF1, AP-1, and HIF-1α, as well as signaling pathway effector molecules such as MAPK/PI3K/Akt, JAK/STAT3, and EGFR/MAPK. Figure created with BioRender

In melanoma cells with BRAF mutations, PD-L1 expression is promoted through the activation of STAT3 and the downstream MAPK signal c-Jun, using MEK inhibitors significantly downregulates MAPK signaling and suppresses the production of PD-L1 [236]. When the Anaplastic Lymphoma Kinase (ALK) is mutated and abnormally activated, it often alters its own kinase activity, thereby activating downstream signaling pathways and leading to tumor development. For example, in ALK^+^ anaplastic large cell lymphoma, NPM-ALK activates downstream MEK-ERK and STAT3 pathways to promote PD-L1 expression [237]. KRAS mutations activate the downstream MEK/ERK signaling pathway, which promotes PD-L1 expression [238]. EGFR mutations, upon stimulation by its ligand EGF, activate downstream signaling pathways such as ERK, mTOR, NF-κBp65, STAT3, and JAK2-STAT1 to promote PD-L1 expression. Inhibiting the MAPK or WNT signaling pathways prevents the upregulation of PD-L1 protein induced by EGF (epidermal growth factor) and IFN-γ, making cancer cells resistant to chemotherapy and conventional cancer treatments [239–241] (Fig. 6).

### Post-transcriptional regulation

#### Non-coding RNAs involved in post-transcriptional regulation of PD-L1

Non-coding RNAs such as miRNA, lncRNA, and circRNA bind the 3’ untranslated region (3’-UTR) of mRNA or act as an upstream regulator to block PD-L1 expression. They are crucial to the development of different types of cancer, their aberrant expression has become an important diagnostic marker and therapeutic candidate.

##### MicroRNAs (miRNAs)

are small non-coding RNAs (18–25 nucleotides) that regulate PD-L1 gene expression by directly binding to the 3’-UTR (direct action) or by targeting other genes involved in the regulation of PD-L1 expression (indirect action) [242].

##### MicroRNAs

**Fig. 7:**
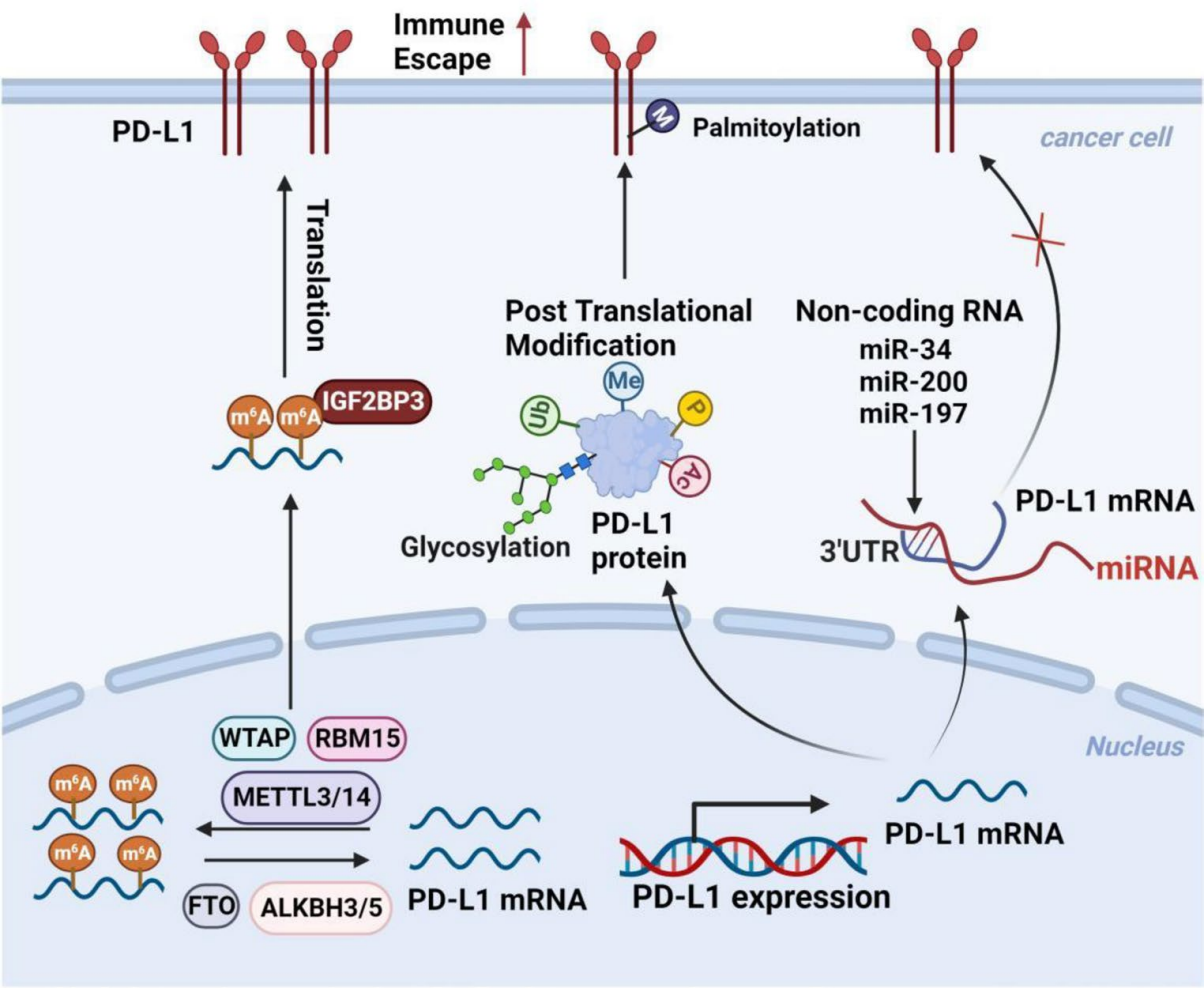
Mechanisms of PD-L1 expression regulation at post-transcriptional and post-translational. Non-coding RNAs such as miR-34, miR-200, and miR-197 can suppress PD-L1 mRNA expression by directly binding to PD-L1 3′UTR. The m^6^A modification of PD-L1 mRNA is critical for regulating the expression and stability of PD-L1 and mediating tumor immune escape. For instance, demethylases such as FTO and ALKBH can remove the m^6^A modification on PD-L1 mRNA, increasing its stability and promoting high PD-L1 expression. The METTL3/IGF2BP3 axis also enhances the stability of PD-L1 mRNA by upregulating its m^6^A modification, further promoting tumor immune evasion. Additionally, post-translational modifications, including phosphorylation, ubiquitination, glycosylation, and palmitoylation can regulate PD-L1 protein expression by influencing its activity, stability, and membrane expression in cancer cells. Figure created with BioRender

directly suppress PD-L1 mRNA post-transcriptional expression. miRNAs bind to the 3′-UTR of mRNA, resulting in mRNA cleavage or translation inhibition, thus negatively regulating the expression of PD-L1 at the post-transcriptional level. In NSCLC, wild-type P53-induced miR-34 directly binds to the 3′-UTR of PD-L1 to inhibit PD-L1 mRNA expression, the therapeutic delivery of miR-34a combined with radiotherapy can increase T cell infiltration and inhibit tumor growth [243]. Probes targeting the PD-L1 3’-UTR in lung cancers also enrich Let-7b miRNA, directly inhibiting PD-L1 expression. Inhalation nebulization treatment with let-7b significantly increases the anti-tumor function of CD8^+^TIL and reduces the expression of PD-L1 in lung cancer cells, showing remarkable tumor inhibitory effects with lower side effects [244]. Daniel et al. find that treating HDLEC (human dermal lymphatic vascular endothelial cells) with the pro-inflammatory cytokines IFN-γ and TNF-α induces miR-155 expression. Further studies reveal that the PD-L1 3’-UTR contains two functional miR-155 binding sites, overexpression of miR-155 inhibits PD-L1 expression, while inhibition of miR-155 results in increased PD-L1 expression following IFN-γ and TNF-α stimulation [245]. However, in diffuse large B-cell lymphoma, miR155 upregulates PD-L1 expression by directly binding to PD-L1 3′-UTR and recruits CD8^+^ T cells in a PD-1/PD-L1-dependent manner [246]. For these contradictory results, we speculate that there may be multiple cytokines or pathways to regulate PD-L1 expression, and miR-155 is just one of them. More mechanisms need to be further investigated (Fig. 7).

##### MicroRNAs

also indirectly regulate PD-1/PD-L1 through upstream and downstream pathways. For example, miR-3127-5p activates the phosphorylation of STAT3 by inhibiting autophagy, thereby inducing the upregulation of PD-L1 expression, inhibiting T cell proliferation and promoting immune evasion and drug resistance in lung cancer cells [247]. After treating breast cancer cells with exosomes derived from cancer-associated fibroblasts, the expression of miR-92 increases. The target gene of miR-92, LATS2, interacts with YAP1 and binds to the enhancer region of PD-L1, thereby enhancing the transcriptional activity of PD-L1 [248]. miR-23a-3p, derived from exosomes released by HCC cells under endoplasmic reticulum stress, upregulates PD-L1 expression in macrophages by the PTEN/AKT pathway and inhibits the function of T cells [249]. In gastric cancer, miR-675-3p (derived from GC-EV) inhibits the expression of the target gene CXXC4 and promotes PD-L1 expression by activating the MAPK pathway, thereby stimulating immune evasion of GC cells [250].

##### Long non-coding RNAs (lncRNAs)

are a highly conserved class of non-coding RNAs longer than 200 nucleotides that have become important regulators of gene expression in various diseases. lncRNA mainly acts as an upstream regulator of PD-1/PD-L1, affecting anti-tumor immunity [251]. Recent research has shown that lncRNAs interact with miRNAs to regulate physiological and pathological processes by inhibiting the transport of target mRNAs or promoting mRNA degradation, thus promoting PD-L1 expression [252]. For example, LncRNA MALAT1 promotes tumor genesis and immune escape in diffuse large B-cell lymphoma by sponging miR-195 to directly target PD-L1 mRNA, while treatment with shMALAT1 increases miR-195 levels and decreases the expression of PD-L1 [253]. LINC01140 directly inhibits miR-377-3p and miR-155-5p expression levels by sponging, and both miR-377-3p and miR-155-5p directly bind to PD-L1 3′-UTR, which leads to upregulation of the downstream target PD-L1 and promotes proliferation, migration and invasion of lung cancer cells [254]. LncRNA HOTAIR also induces PD-L1 overexpression by abnormally activating the TNFα/NF-κB signaling, promoting the immune escape of glioma cells [255]. The latest research shows that the lncRNA IFITM4P upregulates the expression of PD-L1 in oral cancer through a dual mechanism. Firstly, IFITM4P reduces PTEN transcription by increasing the binding of KDM5A to the PTEN promoter in the nucleus, upregulating PD-L1 expression. Secondly, IFITM4P acts as a scaffold in the cytoplasm, recruiting SASH1 to bind and phosphorylate TAK1 (Thr187), thereby promoting phosphorylation of NF-κB (Ser536) and concomitantly inducing PD-L1 expression, inhibiting tumor immune responses [256]. Apart from PD-L1 mRNA, the PD-L1 gene in human LUAD also produces a long non-coding RNA (PD-L1-lnc) by alternative splicing, whose expression is significantly upregulated by IFN-γ. PD-L1-lnc promotes the progression of lung adenocarcinoma by directly binding to c-Myc and enhancing c-Myc transcriptional activity [257].

##### Circular RNAs (circRNAs)

are a kind of functional non-coding circular RNAs that regulate biological processes by mediating RNA alternative splicing (AS), cis-regulation of transcription, and acting as competitive endogenous RNAs [258]. Many CircRNA sequences contain nucleotide binding sites corresponding to miRNAs, functioning as miRNA sponges to participate in post-transcriptional gene expression and regulation, which are of significant importance in the progression, metastasis, treatment resistance, and drug resistance of tumors [259]. The regulation of the PD-1/PD-L1 pathway by circRNAs is based on the binding of miRNAs to immune checkpoint mRNA. In melanoma, circ_0020710 acts as a sponge for miR-370-3p and directly binds to it, which in turn upregulates CXCL12 expression. CXCL12 recruits suppressive immune cells to form an immune-inhibitory microenvironment, ultimately promoting the proliferation, migration and invasion of melanoma cells [260]. In HNSC, miR-382-3p downregulates the expression of PD-L1 through binding to PD-L1 3′-UTR, while circ_0000052 binds to miR-382-3p through a competitive endogenous RNA mechanism, thereby relieving its inhibitory effect on PD-L1 and inducing excessive PD-L1 expression [261]. similarly, circ-CPA4 targets and weakens the expression of let-7 miRNA, then upregulates PD-L1 expression to promote tumorigenesis and EMT in NSCLC cells. Exosomes derived from NSCLC cells, containing PD-L1, also promote stemness and increase the resistance of NSCLC cells to cisplatin [262].

#### Post-transcriptional m^6^A modification of PD-L1

RNA methylation is an important post-transcriptional modification, where the N^6^-methyladenosine (m^6^A) modification serves as the most common type of mRNA methylation in mammals. The m^6^A modification maintains mRNA stability, pre-mRNA splicing, enhances translation efficiency, and promotes polyadenylation and other biological processes. This post-transcriptional m^6^A modification of RNA is dynamically reversible, mainly involving three types of regulatory factors, methyltransferases (writers), demethylases (erasers), and m6A-binding proteins (readers) [263].

M^6^A RNA modifications are critical regulators of PD-L1 expression, stability, and T cell-mediated tumor killing. Reader proteins, including the YTHDF family, IGF2PBs, and HNRNPs, recognize the base information modified by m^6^A methylation and further interact with RNA to exert major regulatory effects, such as participating in downstream translation and mRNA degradation processes [264]. During the process of PD-L1 transcription from DNA to RNA, adenosine is methylated at the N6 position under the action of methyltransferases including METTL3, METTL14, WTAP, and RBM15, these types of methyltransferases are referred to as writers. For example, in breast cancer, METTL3-mediated activation of PD-L1 mRNA is IGF2BP3-dependent, the METTL3/IGF2BP3 axis upregulates the m^6^A modification of PD-L1 mRNA, further increasing the stability of PD-L1 mRNA and inhibiting tumor immune surveillance [265]. In colorectal cancer and melanoma, defects in METTL3 or METTL14 promote IFN-γ-Stat1-Irf1 signaling by stabilizing STAT1 and IRF1 mRNA expression in an m^6^A-YTHDF2-dependent manner, thus upregulating PD-L1 expression and making tumors more sensitive to PD-L1 [266]. In HCC, lipopolysaccharide (LPS) promotes the m^6^A methylation of the lncRNA MIR155HG by upregulating METTL14, thereby maintaining the stability of MIR155HG expression in the reader protein ELAVL1-dependent pathway. Subsequently, MIR155 HG acts as a competitive endogenous RNA through the miR-223/STAT1 axis to upregulate PD-L1 expression, leading to immune escape in HCC cells [267]. Erasers refer to a class of demethylases that mediate the demethylation of m^6^A, mainly including FTO and ALKBH enzymes, making RNA methylation a reversible response. For example, in colorectal cancer, fat mass and obesity-associated protein (FTO) removes the m^6^A modifications of PD-L1 mRNA, enhancing its stability and promoting high expression of PD-L1 in an IFN-γ-independent way. Depleting FTO downregulates PD-L1 expression in colon cancer cells [268]. Xinyao Qiu et al. confirm through N6-methyladenosine sequencing (m^6^A-seq) that PD-L1 mRNA is a direct target of m^6^A modification [269]. The demethylase ALKBH5 maintains the stability of PD-L1 mRNA in intrahepatic cholangiocarcinoma (ICC) cells by interacting with PD-L1 mRNA and inhibiting T cell anti-tumor immunity in a PD-L1-dependent manner. Lack of ALKBH5 enhances m^6^A modification on the PD-L1 3′UTR region and accelerates its degradation in a YTHDF2-dependent manner, thereby reducing PD-L1 protein levels (Fig. 7).

In the above studies, it was observed that m^6^A modification mediated by methyltransferases and demethylases has a dual regulatory role in different types of cancer. So does m6A modification play a role in maintaining or reducing the stability of PD-L1? We speculate that this may not be related to the m^6^A modification itself but depends on the regulatory function of the bound reader protein, it may even be associated with immune cell infiltration in TME. For instance, in a Meta-analysis o*n* the expression and prognosis of m^6^A regulators and PD-L1 in HCC, they find that the transcription of regulators is upregulated in HCC patients and demonstrate a significant association with PD-L1 expression, the expression of the YTHDF family is also significantly related to immune infiltration in the HCC microenvironment. Furthermore, overexpression of YTHDF1/2, YTHDC1, RBM15, and METTL3 is significantly correlated with the clinical stage of hepatocellular carcinoma, while downregulation of ZC3H13 and METTL14 is associated with poor prognosis [270]. In recent years, despite the fact that the study of m^6^A modification has made gratifying progress, its underlying mechanisms in early-stage malignancies remain unknown. Therefore, targeting m^6^A modification holds promise as an unknown field in future research on tumor immunotherapy.

In addition to m^6^A modifications, RNA also undergoes other modifications such as N1-methyladenosine (m^1^A), 5-methylcytosine (m^5^C), and N7-methylguanosine (m^7^G). For example, Yun et al. [271] identify through bioinformatics analysis that m^5^C methylation regulators are related to poor prognosis in patients with pancreatic ductal adenocarcinoma, tumors with high m^5^C scores exhibit lower CD8 T cell infiltration and higher PD-L1 expression levels. In conclusion, the current evidence for the impact of these modifications on the regulation of immune checkpoint molecules remains unclear and requires further investigation.

#### Regulatory mechanisms of PD-L1 expression in post-translational modification

In general, the eIF4F complex, a eukaryotic translation initiation complex, regulates the translation of mRNA encoding STAT1 (one of the main transcriptional regulators of PD-L1) by binding to the RNA sequence in the 5’-UTR of STAT1, thereby modulating the expression of IFN-γ-induced PD-L1 on cancer cells [272].Subsequently, post-translational modifications, including phosphorylation, ubiquitination, glycosylation, and palmitoylation regulate the expression of PD-L1 protein by affecting activity, stability, and membrane expression in cancer cells [273, 274] (Fig. 7).

### N-glycosylation modification of PD-L1 protein

Glycosylation is a major post-translational modification that is important for protein folding, localization, intracellular transport, and immunogenicity [275]. N-glycosylation is the primary form of glycosylation modification for PD-L1, which is crucial in maintaining the stability of PD-L1 itself.

There are multiple regulatory mechanisms for PD-L1 glycosylation in cancer cells. Studies have shown that PD-L1 is N-glycosylated only at N35, N192, N200, and N219, which stabilizes the protein and prevents its degradation by the 26 S proteasome [276]. N-glycosylation modifications at these sites produce a steric hindrance effect, obstructing the interaction between glycogen synthase kinase 3β (GSK3β) and PD-L1, thus stabilizing PD-L1 and suppressing cytotoxic T cell activity. Non-glycosylated PD-L1 is less stable and, when bound to GSK3β, is phosphorylated and degraded through the ubiquitin/proteasome system [277]. For example, in breast cancer, EGF-induced inactivation of GSK3β induces an increase in PD-L1 glycosylation, maintaining stable PD-L1 expression. However, gefitinib destabilizes PD-L1 by inhibiting EGF signaling, enhancing anti-tumor T cell immunity [277]. EGF/EGFR signaling also promotes PD-L1 glycosylation at positions N192 and N200 by upregulating the expression of the glycosyltransferase B3GNT3, ensuring PD-1/PD-L1 binding. The specific recognition of the poly-LacNAc part by tomato lectin blocks the interaction between PD-L1 and PD-1, thereby promoting PD-L1 internalization and degradation [278]. Therefore, constructing monoclonal antibody conjugate complexes (STM108-ADC) targeting the N192 and N200 glycosylation sites of PD-L1 can serve as a promising target for post-translational modifications of immune checkpoints. Research also finds that the N-glycosylation modification of PD-L1 and its binding with PD-1 are closely related to immunosuppression, blocking the glycosylation of PD-L1 greatly improves the anti-tumor effects. For instance, EMT induces N-glycosyltransferase STT3 transcription by β-catenin, which in turn maintains the stability of STT3-dependent PD-L1 N-glycosylation and induces the upregulation of PD-L1 expression in tumor stem cells, enabling immune evasion. This situation can be reversed when using etoposide [279]. In hepatocellular carcinoma, IL-6 induces phosphorylation of the Y112 site of PD-L1 by JAK1, this phosphorylation facilitates STT3A-catalyzed glycosylation of PD-L1. Blocking the IL-6/JAK1 signaling axis inhibits the binding of STT3A to PD-L1, leading to PD-L1 degradation [280]. N-glycosylation of the PD-L1 protein is detrimental to the detection of PD-L1, making it difficult to bind with diagnostic antibodies for PD-L1. After the glycan chains of PD-L1 are hydrolyzed using N-glycosidase (PNGase F), the detection rate of the PD-L1 protein is significantly improved, increasing the affinity for binding to PD-L1 antibodies [281]. Therefore, deglycosylated PD-L1 protein is expected to become a biomarker for guiding cancer immunotherapy.

### **Phosphorylation modification of PD-L1 protein**

PD-L1 phosphorylation sites are predominantly located in the extracellular domain, different kinases induce PD-L1 phosphorylation at different sites, producing diverse effects. For example, when using EGFR inhibitors activates GSK3α by inhibiting AKT activity, subsequently GSK3α promotes the phosphorylation of Ser279 and Ser283 residues of PD-L1 [282]. Meanwhile, GSK3β, an isomer of GSK3α, induces non-glycosylated PD-L1 to phosphorylate, resulting in polyubiquitination and degradation of PD-L1 in the cytoplasm [277]. PARP1 inhibitors olaparib [283] and c-MET inhibitors [284] can downregulate PD-L1 expression by modulating GSK3β activity. Furthermore, AMPK activated by metformin directly binds to PD-L1 in the endoplasmic reticulum (ER), causing PD-L1 to be phosphorylated at the S195 site, leading to abnormal glycosylation of PD-L1 and endoplasmic reticulum associated degradation (ERAD) [285]. The energy state changes induced by the ketogenic diet activate AMPK, which then phosphorylates the Ser283 site on PD-L1, thereby destroying its interaction with CMTM4, subsequently triggering PD-L1 degradation through lysosomes [286].

## **Ubiquitination modification of PD-L1 protein**

Ubiquitination modification refers to the processes where a ubiquitin molecule covalently binds to a target protein molecule under the action of E1, E2, and E3 ligases and specifically modifies it. When the target protein binds to a single ubiquitin molecule, it is called monoubiquitination. When multiple lysines of the target protein are simultaneously tagged by a single ubiquitin molecule, it is referred to as polyubiquitination [287]. PD-L1 expression is highly regulated by ubiquitin-proteasome systems (UPS), such as STUB1 and SPOP, promoting PD-L1 polyubiquitination and degradation, while CSN5 and the deubiquitinating enzyme DUB antagonize its degradation [288].

### Ubiquitin ligase

NEDD4 is an E3 ubiquitin ligase of the HECT domain family that acts as a regulator of PD-L1 ubiquitination. In bladder cancer, NEDD4 is phosphorylated by FGFR3, followed by interacting with PD-L1 and catalyzing its polyubiquitination via Lys48 (K48) linkage [289]. Within the ER, the E3 ubiquitin ligase HRD1 (HMG-CoA reductase degradation 1) initiates the polyubiquitination of abnormally glycosylated PD-L1, resulting in endoplasmic reticulum associated degradation [285]. CyclinD-CDK4 and the Cullin3^SPOP^ E3 ligase regulate PD-L1 expression through ubiquitin-proteasome system-mediated degradation. During the S and G2 phases, CyclinD-CDK4 phosphorylates SPOP (speckle type BTB/POZ protein) and dissociates it from Cullin 3, thereby enhancing the stability of Cullin 3, inducing PD-L1 polyubiquitination and reducing PD-L1 expression levels. Inhibition of CDK4 and CDK6 in vivo reduces SPOP phosphorylation, promoting APC/Cdh1-mediated SPOP degradation, resulting in high PD-L1 expression [290]. Additionally, inhibition of CDK5 increases ubiquitin E3 ligase FBXO22 levels, which degrades PD-L1 and makes tumor cells more sensitive to DNA damage [291]. In breast cancer, the transmembrane and ubiquitin-like structural domain-containing protein 1 (TMUB1) competes with HUWE1 (an E3 ubiquitin ligase) for binding to PD-L1 and inhibiting its ubiquitination at K281 in the ER [292]. The E3 ubiquitin ligase STUB1 (STIP1 homology and U-box containing protein 1) also polyubiquitinates PD-L1 in the cytoplasmic domain at lysine residues and downregulates PD-L1 expression, whereas CMTM6 maintains PD-L1 stability by blocking ubiquitination in tumor cells [293]. Wu et al. identify a novel E3 ubiquitin ligase ARIH1 that targets the PD-L1 protein. In breast cancer cells, ES-072 (an EGFR inhibitor) increases the phosphorylation levels at S279 and S283 sites of the PD-L1 protein intracellular segment through the EGFR-GSK3α pathway, promoting the interaction between PD-L1 and ARIH1, thereby regulating PD-L1 degradation through the proteasome and promoting anti-tumor immunity [282].

### Deubiquitinating enzymes(DUB)

Ubiquitination is a dynamic and reversible modification in which ubiquitin molecules and polyubiquitin chains on target proteins can be dissociated by deubiquitinating enzymes, thus stabilizing PD-L1 protein levels in cancer cells. For example, in gastric cancer, PD-L1 expression is positively related to the expression of ubiquitin-specific protease 7 (USP7), which directly interacts with PD-L1 and promotes its deubiquitination and stabilization. Targeting USP7 enhances CD8 T cell infiltration and cytotoxicity in TME and reduces PD-L1 expression in tumor cells [294]. In pancreatic cancer, ubiquitin-specific protease 8 (USP8) catalyzes the deubiquitination of PD-L1 by inhibiting ubiquitin-regulated proteasome degradation, the combined therapy of USP8 inhibitors with anti-PD-L1 effectively inhibits pancreatic tumor growth by activating cytotoxic T cells [295]. In hepatocellular carcinoma, the deubiquitinating enzyme USP22 directly interacts with the C-terminus of PD-L1, catalyzing its deubiquitination and stabilization. USP22 is highly expressed in HCC, closely correlated with poor prognosis in patients [296]. Furthermore, pro-inflammatory cytokines enhance the deubiquitination of PD-L1, thereby increasing its stability. For example, TNF-α released by M2 macrophages induces the binding of NF-κB p65 to the CSN5 promoter, promoting CSN5 transcription and increasing CSN5 expression. Subsequently, TNF-α also inhibits the ubiquitination and degradation of PD-L1 protein through COP9 signalosome subunit 5 (CSN5), thereby enhancing the stability of PD-L1 and sensitizing cancer cells to immunotherapy [297].

#### Palmitoylation modification of PD-L1

Palmitoylation modification refers to the attachment of fatty acyl groups to the cysteines of the target proteins through thioester bonds, which in turn regulates the membrane localization and function of the protein. Recent studies have indicated that palmitoylation of PD-L1 has become a novel post-translational modification, which is critical for maintaining PD-L1 stability and improving the efficacy of tumor immunotherapy. However, it is still in the research stage [298].

In different types of tumors, the palmitoyltransferases mediating the palmitoylation modification of PD-L1 may vary. In the study conducted by the Xu lab using a mouse model of colon cancer, the cysteine residue at position 272 (Cys272) of the PD-L1 protein is palmitoylated by the palmitoyltransferase DHHC3 (aspartate-histidine-histidine-cysteine 3), thereby inhibiting the ubiquitination and degradation of PD-L1 and increasing PD-L1 expression levels. Treatment with 2-bromopalmitate or silencing DHHC3 activates anti-tumor immunity by inhibiting PD-L1 palmitoylation. They also designed a competitive inhibitor of PD-L1 palmitoylation according to the amino acid sequences near the Cys272 site, which specifically inhibits PD-L1 palmitoylation, reduces PD-L1 expression in cancer cells and enhances the anti-tumor immune responses mediated by T cells [274]. In the same year, Yang et al. also reported that in breast cancer, the palmitoyl transferase DHHC9 (aspartate-histidine-histidine-cysteine 9) mediates palmitoylation modification of PD-L1. DHHC9 covalently attaches palmitate to cysteine residues(C272) of PD-L1, thereby blocking the ubiquitination of PD-L1 to increase its stability. Disrupting PD-L1 palmitoylation through specific point mutations at the Cys272 site or inhibiting ZDHHC9 expression makes breast cancer cells sensitive to T-cell killing, thereby inhibiting tumor growth [299]. PD-L1 is highly palmitoylated in cisplatin-resistant bladder cancer cells, inhibition of fatty acid synthase (FASN) suppresses PD-L1 expression and palmitoylation, suggesting that the FASN-PD-L1 targeting therapy may be used for bladder cancer patients [300].

#### Lysosomal-mediated degradation

In addition to the above proteasome degradation pathways, lysosomes in cells also participate in the degradation of various endocytic proteins. PD-1/PD-L1, as a cell membrane protein, can be imported into lysosomes for degradation. For example, the HIP1R protein carries a lysosomal targeting signal and delivers PD-L1 to lysosomes for degradation by binding to PD-L1, thereby enhancing the killing effect of T cells on cancer cells. Moreover, a peptide (PD-LYSO) designed based on the lysosomal sorting signals and the binding sequences of HIP1R and PD-L1can successfully deplete PD-L1 expression in tumor cells [301]. Furthermore, cancer cells have also evolved mechanisms to enhance PD-L1 stability and inhibit lysosomal degradation. The transmembrane proteins CMTM4 and CMTM6 (CKLF-like MARVEL transmembrane domain containing 4/6) are key regulators of PD-L1, primarily stabilizing PD-L1 protein expression through the lysosomal pathway [293]. The binding of CMTM6 to PD-L1 inhibits the lysosomal degradation of PD-L1, after knocking out CMTM6, the E3 ubiquitin ligase STUB1 induces PD-L1 polyubiquitination in the circulating endosomes, thereby degrading it via the lysosomal pathway [302].

### Regulation of PD-L1 by tumor microenvironment

Previous research has concentrated on the intrinsic mechanisms regulating PD-L1 expression in tumor cells, but the expression of PD-L1 is also influenced by a pro-inflammatory tumor microenvironment (TME) created by exposure to infiltrating immune cells, which includes pro-inflammatory cytokines, exosomes, metabolites, etc [303]. Beyond the aforementioned interferon signaling pathways, there are many specific cytokines within the TME that significantly modulate the expression of PD-1/PD-L1. For example, IL-17 A increases PD-L1 expression in colorectal cancer through the p65/NRF1/miR-15b-5p pathway and promotes resistance to anti-PD-1 therapies [304]. IL-6 derived from glioblastoma induces PD-L1 expression on bone marrow cells via a STAT3-dependent mechanism, therapeutic blockade of IL-6 signaling inhibits tumor growth and improves survival rates [305]. Tumor-derived TNF-α induces PD-L1 expression on mast cells through activating the NF-κB pathway, thus inhibiting T cell immunity and gastric cancer progression [306]. TGF-β from TAM inhibits T cell activity by reducing the expression levels of IFN-γ and granzyme B through the inhibition of Smad2/3 phosphorylation and mitochondrial respiration [307]. Nutrients in the tumor microenvironment, especially glucose, induce cancer cells to upregulate PD-L1 expression. Specifically, in human glioblastoma cells, high glucose levels promote the dissociation of hexokinase 2 (HK2) from mitochondria, subsequently mediating phosphorylation at the IκBα T291 site and promoting IκBα degradation, thus activating the NF-κB pathway and upregulating PD-L1 expression at the transcriptional level. Combined treatment with an HK2 inhibitor and anti-PD-1 antibody significantly inhibits tumor growth in mice and prolongs survival [308]. Exosomes secreted by cells contain abundant miRNAs, mRNAs, and functional proteins, which mediate intercellular signaling in the TME [309]. Bo Gong et al. find two secretory PD-L1 splicing variants lacking transmembrane domains in aPD-L1-resistant NSCLC patients, which can mediate resistance to PD-L1 antibody blockade treatment by inhibiting T cell activity [310]. Exosomes derived from glioblastoma stem cells further enhance immunosuppression by upregulating phosphorylated STAT3, thus promoting macrophage polarization to M2 macrophages and increasing PD-L1 expression [311]. Exosomes released by metastatic melanoma carry PD-L1 on their surface, the levels of circulating exosomal PD-L1 are positively correlated with IFN-γ levels. IFN-γ stimulation increases the amount of PD-L1 on these vesicles, thereby inhibiting CD8 T cell function and promoting tumor growth [312]. These findings collectively highlight the crucial roles of various molecules in PD-L1 regulation in different cancer cells, indicating their potential as targets for immunotherapy.

In summary, PD-1/PD-L1 expression is subject to multiple regulatory mechanisms and varies greatly at different stages of tumor development. Given the unique microenvironment and intricate signaling pathways in tumors, it is necessary for future research to delve into which PD-1/PD-L1 regulatory mechanisms play a key role in certain types of cancer. These studies will advance our understanding of tumor immune evasion mechanisms and provide new insights for tumor immunotherapy. We believe that in the near future, as the veil is progressively lifted on the various regulatory mechanisms governing the expression of immune checkpoint molecules, the prospects for inhibiting tumor immunity via these checkpoints will be more promising.

### Blockade of PD-1/PD-L1 signaling in tumor immunotherapy

Nowadays, tumor immunotherapy, compared to traditional therapy methods such as surgery and chemotherapy, focuses more on utilizing own immune system to eliminate tumors. Due to its fewer adverse side effects and broader applicability, it has made significant progress in treating various types of cancer. Currently, the therapeutic approaches targeting PD-1/PD-L1 include immune checkpoint inhibitors, adoptive T cell therapy (ACT), gene editing therapies, and tumor vaccines, which have been widely applied clinically in advanced cancer patients, particularly in melanoma and lung cancer, achieving remarkable early success and greatly improving the overall survival and progression-free survival of cancer patients [313]. Unfortunately, there are still limitations such as low response rates and resistance in some cancer patients, resulting in only a minority of patients benefiting clinically. Therefore, identifying suitable candidates for immune checkpoint inhibitor therapy, clarifying patient resistance mechanisms, and exploring novel strategies for combined drug immunotherapy hold significant research significance.

With the discovery of immune escape mechanisms, drug development has been greatly advanced. At present, ICIs based on the PD-1/PD-L1 axis have received clinical approval in many countries. These ICIs reactivate T cell immune responses against tumors by inhibiting immune checkpoints, thereby enhancing immune system surveillance and exerting anti-tumor effects [314]. Antibodies targeting the PD-1/PD-L1 pathway are already approved to treat various human cancers, such as head and neck cancer (pembrolizumab and nivolumab), melanoma (pembrolizumab and nivolumab), urothelial carcinoma (nivolumab, pembrolizumab, durvalumab, atezolizumab, and avelumab), non-small cell lung cancer (pembrolizumab, nivolumab, and atezolizumab), renal cell carcinoma (nivolumab), Hodgkin lymphoma (pembrolizumab and nivolumab), skin squamous cell carcinoma (cemiplimab), and so on [315, 316]. However, these therapies need to be used cautiously, as most patients will develop drug resistance. Excessive blockade of PD-1/PD-L1 may lead to autoimmune diseases and produce side effects.

### Targeting PD-1/PD-L1 by antibodies

PD-1 or PD-L1 antibodies can specifically block the binding between PD-1 and PD-L1, thereby reactivating T cells and restoring anti-tumor immune function, making them potential anti-tumor drugs. The drug development teams utilize the molecular targeting principle of monoclonal antibodies and the theory of tumor immunology as the basis for developing PD-1 and PD-L1 monoclonal antibodies. PD-1 monoclonal antibodies work by reducing the stimulatory signals produced by the binding of PD-1/PD-L1, restoring the T cell immune responses and enhancing T cell attack against cancer cells. PD-L1 monoclonal antibodies follow the principle that drug molecules preferentially target PD-L1 on cancer cells, reducing the negative regulatory factors released by cancer cells to the immune system [317]. Moreover, PD-L1 inhibitors are particularly effective against cancers with a high mutation burden, such as colon cancer with microsatellite instability. A*s* heightened mutagenicity triggers responses from pre-existing T cells, using PD-L1 inhibitors reduces the immunosuppressive effect on T cells [318]. So far, six immune checkpoint inhibitors targeting PD-1 or PD-L1 have already been approved for clinical therapy in cancer patients, including PD-1 inhibitors like pembrolizumab, nivolumab, cemiplimab, and PD-L1 inhibitors like atezolizumab, avelumab, and durvalumab [319] (Table 1).

#### Anti-PD-1 antibody

#### Pembrolizumab

Pembrolizumab (trade name Keytruda) is a high-affinity humanized IgG4 anti-PD-1 antibody, it was the first treatment globally approved by the FDA for patients with metastatic melanoma [320] and the first therapy approved to treat all high-mutated solid tumors [321]. Phase I clinical trials have proven the safety and efficacy of pembrolizumab in treating melanoma, and phase II clinical trials have shown that pembrolizumab’s efficacy in treating advanced melanoma is significantly superior to that of ipilimumab (anti-CTLA-4 antibody) [322]. Due to its significant anti-tumor effects observed in clinical trials, pembrolizumab has been approved for first-line therapy in various advanced melanomas [323]. Subsequently, its indications have extended to recurrent head and neck squamous cell carcinoma [324], advanced or metastatic NSCLC with PD-L1 positivity [325], metastatic urothelial carcinoma [326], cervical cancer [327], classical Hodgkin lymphoma [328], microsatellite-unstable or mismatch repair-deficient cancer [329], gastric cancer [330] and primary mediastinal large B-cell lymphoma [331], among others. In conclusion, pembrolizumab treatment has greatly improved the overall survival of cancer patients.

#### Nivolumab

Nivolumab (trade name Opdivo) is a humanized IgG4 anti-PD-1 antibody that exhibits high affinity for PD-1, effectively blocking the binding between PD-1 and PD-L1 [332]. Nivolumab can be used to treat unresectable or metastatic melanoma following Ipilimumab (anti-CTLA-4), with a standalone objective response rate (ORR) of 31.7% [333]. After the success of the phase I-III clinical trial, Nivolumab is approved as a first-line treatment for metastatic melanoma [334], second-line therapy for NSCLC [335] and renal cell carcinoma (RCC) [336]. In NSCLC patients, nivolumab’s objective response rate is 23.7%, significantly prolonging the overall survival, response rate, and progression-free survival (PFS) in patients in a manner independent of PD-L1 expression [337]. Additionally, Nivolumab has been demonstrated to be effective against many other malignancies, including classical Hodgkins lymphoma [173], recurrent head and neck squamous cell carcinoma [338], locally advanced or metastatic urothelial carcinoma [339], and microsatellite instability or mismatch repair-deficient colorectal cancer [340].

### Cemiplimab

Cemiplimab (trade name Libtayo) is an anti-PD-1 antibody with high affinity and is the first immune checkpoint therapeutic drug designed for the specific treatment of metastatic cutaneous squamous cell carcinoma (CSCC) [341]. Clinical phase 1 trials in advanced CSCC patients demonstrated that after Cemiplimab treatment, there were no signs of disease recurrence for over 16 months, and the response was persistent [342]. The phase II trial of Cemiplimab treatment showed an objective response rate of up to 47% [343]. Subsequently, Cemiplimab has also been approved for the first-line clinical therapy of advanced NSCLC with high PD-L1 expression [344] and metastatic basal cell carcinoma [345].

In addition, four other PD-1 antibodies (Sintilima, Toripalimab, Penpilimab, and Zzimberelimab) have been approved in China for the therapy of resectable or metastatic melanoma, nasopharyngeal carcinoma, urothelial carcinoma, as well as relapsed or refractory classical Hodgkin lymphoma [346].

### Anti-PD-L1 antibody

#### Atezolizumab

Atezolizumab (trade name Tecentriq) is a phage-derived human IgG1 antibody with a genetically engineered modified Fc fragment. Consequently, Atezolizumab can block PD-L1 on the tumor surface while avoiding antibody-dependent cell-mediated cytotoxicity effects [347]. As a PD-L1 immune checkpoint inhibitor, Atezolizumab has demonstrated significant efficacy in various cancers, including breast cancer, renal cell carcinoma, and bladder transitional cell carcinoma. It significantly improves the survival rate of NSCLC patients, and this response is associated with PD-L1 expression in tumor-infiltrating immune cells [348]. In advanced or metastatic urothelial carcinoma, atezolizumab is also approved as a first-line treatment, where increased PD-L1 expression on immune cells can enhance the responses to Atezolizumab [349]. Atezolizumab may also be applied to patients with EGFR-sensitive mutations who are receiving targeted therapy [350].

#### Avelumab

Avelumab (trade name Bavencio) is a human IgG1 monoclonal antibody that has been approved for the therapy of refractory metastatic urothelial carcinoma [351] and Merkel cell carcinoma [352]. The therapeutic action of Avelumab involves the blockade of PD-L1 to reinvigorate T cells and induce the occurrence of antibody-dependent cell-mediated cytotoxicity through its natural Fc region [353]. In the therapy of metastatic Merkel cell carcinoma, Avelumab has shown an objective response rate of 62.1% [354], and many other studies on Avelumab as a monotherapy or in combination with other therapies are underway.

#### Duravulumab

Duravulumab (trade name Imfinzi) is a humanized antibody that can directly target PD-L1, blocking the interaction between PD-L1 and PD-1, thereby preventing tumor immune evasion and enhancing the immune responses [355]. Durvalumab is mainly approved for the therapy of platinum-resistant primary advanced or metastatic urothelial carcinoma in PD-L1-positive patients [356] and small cell lung cancer [357]. According to reports, Durvalumab’s overall response rate (ORR) in the treatment of HNSCC is 9.2% [358], with progression-free survival rates of 20% for all HNSCC patients, reaching 25% in PD-L1-positive patients [359]. In NSCLC, the ORR of Durvalumab treatment can reach 66.3%, significantly extending the overall survival of patients with phase III and unresectable NSCLC [360]. The drug has tolerable toxicity characteristics and appears to be more effective in patients with high PDL-1 expression. However, further research is needed to determine its efficacy at different disease stages and its applicability to other tumor types.

**Table 1:** Clinical application of anti-PD-1/PD-L1 drugs

Target	Name	class	Tumor type	Adverse events
PD-1	Pembrolizumab	IgG4,human	Advanced melanoma, head and neck squamous cell carcinoma, non-small cell lung cancer, etc.	Arthralgia, pneumonia, hepatotoxicity, etc.
	Nivolumab	IgG4,human	Metastatic melanoma, non-small-cell lung cancer, renal cell carcinoma, etc.	Endocrine toxicity, cardiotoxicity, etc.
	Cemiplimab	IgG4,human	Advanced non-small cell lung cancer, metastatic basal cell carcinoma, etc.	Rash, gastrointestinal reactions, liver toxicity, etc.
PD-L1	Atezolizumab	IgG1,human	Advanced or metastatic urothelial carcinoma, breast cancer, etc.	Hyperthyroidism, nausea and vomiting, etc.
	Avelumab	IgG1,human	Refractory metastatic urothelial carcinoma, metastatic merkel cell carcinoma, etc.	Rash, pneumonia, endocrine disease, fatigue, etc.
	Durvalumab	IgG1,human	Platinum-resistant primary advanced or metastatic urothelial carcinoma, Stage III Non-Small-Cell Lung Cancer	Liver injury, gastrointestinal injury, dyspnea, etc.

### Targeting PD-1/PD-L1 by small molecule inhibitors

Due to the fact that large molecule monoclonal antibodies such as PD-1 and PD-L1 monoclonal antibodies only recognize receptors on the surface of cells and cannot act directly in the cells, resulting in low drug utilization, long-term use may easily cause acquired drug resistance. Consequently, combination therapy with other treatments is necessary to improve therapeutic efficacy. In recent years, researchers have expanded the inhibition of PD-1/PD-L1 pathways into the realm of traditional small-molecule drugs. Non-monoclonal antibodies that block PD-1/PD-L1 binding, such as small molecule inhibitors, cyclic peptide inhibitors, and other macrocyclic compounds, have gained more and more attention [361, 362]. In the following discussion, we will focus on PD-1/PD-L1 small-molecule inhibitors.

Small-molecule inhibitors are divided into sulfonamides, biphenyls, oxadiazoles, and other heterocyclic compounds [363]. In the molecular structure, the transmembrane protein PD-L1 participates in PD-1/PD-L1 binding in a dimeric form. While the small-molecule compounds BMS202 and BMS-8 act on the PD-L1 protein, causing PD-L1 to form dimers. Subsequently, BMS202 and BMS-8 bind to the cylindrical hydrophobic cavities of the two dimerized PD-L1s respectively, preventing normal interaction between PD-1 and PD-L1 [364], reducing the negative regulation of immune function by PD-1/PD-L1. Additionally, the BMS family’s BMS-1001 and BMS-1166 alleviate PD-1/PD-L1-mediated Jurkat T lymphocyte exhaustion by inhibiting PD-L1, activating T lymphocyte immune activity [365].

The research findings on small-molecule drugs for tumor immunotherapy currently require extensive clinical trials for validation, and many compounds with potential biological activities are still in the preclinical research stage. With in-depth research on the interaction information between PD-1 and PD-L1 and the surface binding of the formed dimers, it will provide an essential structural basis for the design of small-molecule drugs. Furthermore, there are many tumor-associated regulatory factors and signaling pathways, which also offer abundant targets for small-molecule compounds.

### Combination therapy

Owing to the heterogeneity of cancers and genetic differences between individuals, the efficacy of targeting the PD-1/PD-L1 pathway alone is unsatisfactory [366]. Therefore, there is an urgent need for combination therapies that can be tailored to different patients in order to increase the response rates to PD-1/PD-L1 inhibitors and overcome resistance to anti-PD-1/PD-L1 therapy. Recent studies have shown that combination therapies such as radiotherapy and chemotherapy, targeted therapies such as VEGF/VEGFR, cellular therapy, tumor vaccines, and oncolytic viruses can regulate PD-L1 in certain cancers [10, 12], making them ideal candidates for combination therapy with anti-PD-1/PD-L1. These approaches have achieved initial success in treating various types of cancer.

### PD-1/PD-L1 inhibitors combined with other immune checkpoint inhibitors (ICIs)

#### PD-1/PD-L1 inhibitor combined with CTLA-4 inhibitor treatment

In T cells, CTLA-4 generates potent inhibitory signaling to prevent T cell activation and growth. Blocking CTLA-4 inhibits Tregs, reactivates T cells suppressed within the TME, and accompanies an increase in IFN-γ production, thereby inducing PD-L1 expression in the TME and subsequently inhibiting T cell anti-tumor responses [367]. For certain patients with low PD-L1 expression, CTLA-4 reduces the risk of resistance to PD-1 and demonstrates synergistic anti-tumor effects. Mounting evidence suggests that the dual blockade of PD-1/PD-L1 and CTLA-4 exhibits stronger anti-tumor activity in certain cancer types [368]. It has become the most widely used and deeply explored immunotherapy approach, approved for the therapy of metastatic melanoma, advanced renal cell carcinoma, and mismatch repair deficient/highly microsatellite instable colon cancer. When the PD-1 inhibitor Nivolumab is combined with the CTLA-4 inhibitor Ipilimumab, it significantly increases the progression-free survival rate and median overall survival in advanced NSCLC patients, greatly improving the patient prognosis with more durable therapeutic effects [369]. In B16 melanoma, the combined blockade of CTLA-4 and PD-1 negative co-stimulatory receptors significantly increases the Teff infiltration and production of inflammatory cytokines in tumors, and reduces Tregs and myeloid cells within the tumor, thus counteracting tumor-induced immune suppression and promoting tumor rejection [370]. In advanced melanoma, the five-year survival rates of combined therapy with Nivolumab and Ipilimumab reach 52%, with a positive response rate of 63.8% in clinical phase II and III trials [371, 372]. In clinical phase III trials of patients with metastatic renal cell carcinoma (mRCC) [373] or unresectable malignant pleural mesothelioma (CheckMate 743) [374], similar efficacy is observed in the combination of anti-PD-1 (nivolumab) and anti-CTLA-4 (ipilimumab) treatment, significantly improving patient overall survival. Moreover, a meta-analysis by Kongju et al. [368] reveals that the combination therapy of PD-1 with CTLA-4 is a feasible strategy, offering enhanced efficacy and acceptable adverse events.

### PD-1/PD-L1 inhibitors combined with other ICIs

In addition to targeting CTLA-4, other dual immune checkpoint blockade strategies, such as PD-1/PD-L1 inhibitors combined with immune checkpoint inhibitors like TIM-3, TIGIT, LAG-3, and PVRIG, are still under clinical trials [375]. LAG-3 and PD-1 synergistically regulate T cell functions, thereby attenuating anti-tumor immune responses. In untreated metastatic or inoperable melanoma patients, combination treatment of Relatlimab (an anti-LAG-3 monoclonal antibody) with nivolumab demonstrates anti-tumor activity, significantly improving patients’ progression-free survival [376]. TIM-3 inhibits anti-cancer immunity and mediates resistance to PD-1 and PD-L1 inhibitors. Clinical Phase Ia/b results of LY3321367 (a novel TIM-3 monoclonal antibody) show that the ORR and DCR (disease control rate) of PD-1 or PD-L1 inhibitors combined with TIM-3 inhibitors are 4% and 42%, respectively, with favorable pharmacokinetics/pharmacodynamics [377]. In the CITYSCAPE trial, PD-1 or PD-L1 inhibitors combined with a novel anti-TIGIT inhibitory immune checkpoint drug (Tiragolumab) showed clinically meaningful improvements in objective remission rates and progression-free survival rates, serving as a potential first-line therapy for locally advanced unresectable or metastatic NSCLC [378].

Furthermore, PD-1/PD-L1 inhibitors also regulate T cell function by combining with immune checkpoint agonists like CD137 (4-1BB), ICOS, CD134 (OX40), GITR, and CD27. For example, the results of a phase Ib clinical trial (NCT02179918) find that in advanced solid tumor patients treated with Utomilumab (a human monoclonal antibody agonist targeting the T cell co-stimulatory receptor CD137) combined with pembrolizumab, higher levels of activated memory/effector peripheral blood CD8 T cells are observed, leading to profound and durable anti-tumor activity and high safety [379]. Several ongoing clinical studies are exploring the combination of other co-stimulatory molecule agonists with PD-1/PD-L1 antibodies. Initial clinical data indicate that this therapy has superior anti-tumor efficacy, thus providing a solid scientific basis for subsequent cancer immunotherapy.

### PD-1/PD-L1 inhibitors combined with targeted drugs

Targeted therapy can precisely target transcription regulatory factors to activate or inhibit key signaling pathways in cellular metabolism, it activates T cells to exert immune functions and inhibits tumor cell proliferation, thus inhibiting tumor disease progression at the level of cellular metabolism. Since PD-1/PD-L1 antibodies take longer to take effect, typically 2 to 3 months, combining PD-L1/PD1 immunotherapy with targeted therapy significantly improves therapeutic effects.

### PD-1/PD-L1 inhibitors combined with anti-angiogenic drugs

Tumor growth depends on the formation of new blood vessels to ensure a continuous supply of oxygen and nutrients. Targeting the inhibition of tumor vasculature is primarily achieved by blocking the pathways of tumor angiogenesis, cutting off the nutrient supply to tumor cells and achieving a treatment for inhibiting tumor growth [380]. However, studies have found that inhibiting tumor angiogenesis may further aggravate vascular damage, making it difficult for drugs that kill cancer cells to reach the interior of the tumor. With the proposal and clinical application of the “vascular normalization” theory, new progress has been made in tumor treatment. The theory aims to induce structural and functional changes in tumor blood vessels by using anti-angiogenic drugs, rendering them more similar to normal blood vessels, thereby increasing blood flow and allowing cytotoxic drugs to more easily penetrate the tumors [381, 382]. Although vessel normalization delays tumor progression, the underlying regulatory mechanisms are poorly understood. Zhang’s team discovered that gene expression related to vascular normalization is linked to the activation of T-lymphocytes’ immune signaling pathways, helper T (Th) cells are crucial in vascular normalization and immune programming. In mice with defective vascular normalization, Th cell infiltration in tumors is reduced. When Th cells are activated using immune checkpoint blockade methods, the ability for vascular normalization is strengthened [383]. This suggests that there is mutual regulation between vascular normalization and the immune system. Huang et al. also discover that in mouse breast cancer models, using a low dose of vascular normalization agent, anti-VEGFR2 antibodies, can increase CD4^+^ and CD8^+^ T cell infiltration, reprogram the tumor microenvironment, and enhance the efficacy of immunotherapy [384]. Therefore, anti-angiogenesis therapy can not only suppress angiogenesis but also obstruct the accumulation of immunosuppressive cells through vascular normalization and promote the influx of effector T cells into tumors.

Currently, vascular endothelial growth factor (VEGF) is the major target of anti-angiogenic agents, which mainly sends signals through VEGFR2 and is critical for regulating the biological function of endothelial cells, vascular permeability, and angiogenesis [385]. Extensive clinical data indicate that the combination of PD-1/PD-L1 blockade with anti-angiogenic drugs significantly enhances the efficacy of PD-1/PD-L1 inhibitors and improves patient survival. Studies have found that anti-VEGF or VEGFR2 upregulates PD-L1 expression levels, related to the extent of CTL activity in tumors. Therefore, VEGF/VEGFR monoclonal antibodies or tyrosine kinase inhibitors make tumor cells more sensitive to PD-L1 inhibitors, combining them with PD-L1 inhibitors enhances the sensitivity and effectiveness of anti-angiogenic therapy [386]. Clinically, anti-angiogenic drugs are mainly divided into monoclonal antibodies targeting VEGF and VEGFR, and tyrosine kinase inhibitors (TKI) blocking the intracellular domain of VEGFR [387]. For example, patients with surgically unresectable HCC who received combination therapy with Bevacizumab (a VEGF antibody) and Atezolizumab (a PD-L1 antibody) have an objective response rate of 36% and a disease control rate of 71%, with a reduced risk of death [388]. This indicates that the combination therapy scheme can provide longer progression-free survival (PFS) than targeting PD-L1 alone. Subsequently, to compare this combination treatment with the current standard first-line therapy for HCC (sorafenib monotherapy), the Phase 3 IMbrave150 clinical trial (NCT03434379) is conducted. The data indicate that the overall survival rate with ATZ and BVZ combination therapy is 67.2% at 12 months, with a 42% reduction in the risk of death and significant improvements in OS and PFS compared to sorafenib treatment [389]. Therefore, combining atezolizumab and bevacizumab may improve the anti-tumor immune responses, providing more lasting clinical benefits for patients with hepatocellular carcinoma.

Moreover, Hiroto Kikuchi et al. find that when hepatocellular carcinoma is first treated with PD-1 inhibitors, subsequent administration of the anti-angiogenic agent sorafenib (a multi-kinase inhibitor with anti-VEGFR1-3 activity) can enhance the response of hepatocellular carcinoma to sorafenib. This enhancement is accompanied by an increase in CD8 T cell infiltration and the promotion of tumor vessel normalization, thereby inducing an effective anti-tumor immune response and increasing the survival rate of mice [390]. In a clinical phase 1b trial for advanced RCC patients, the combination of the VEGF receptor tyrosine kinase inhibitor Axitinib and the PD-1 monoclonal antibody Pembrolizumab yielded encouraging results. Out of the patients treated, 73% achieve objective responses, with 8% achieving complete remission, and more than 90% of patients exhibit tumor shrinkage, the median progression-free survival exceeds 20 months [391]. This indicates that the combined therapy of Axitinib and Pembrolizumab is safe and tolerable in the initial treatment of patients with advanced RCC, offering unprecedented anti-tumor activity. Subsequently, Rini et al. performed a phase III clinical trial for advanced RCC, enrolling a total of 861 patients. The findings reveal that compared to the standard targeted therapy drug Sunitinib for renal cancer, the combination therapy of Axitinib and Pembrolizumab achieves an objective remission rate of nearly 60%, with a maximum reduction of 47% in the risk of death [392]. This suggests that the combination therapy significantly prolongs OS and PFS, and achieves higher objective response rates, making it a potential first-line therapy for advanced RCC. In the same year, Motzer et al. conducted a comparison between PD-L1 antibody avelumab combined with Axitinib and Sunitinib monotherapy in 886 patients with advanced renal cancer. The results show that in 560 PD-L1 positive patients, the combination therapy significantly increased the objective response rates, there was little difference in side effects between the two groups during the treatment period [393]. Moreover, Ciccarese et al. conducted a meta-analysis and revealed that VEGFR-TKIs in combination with ICIs significantly improved the PFS of patients with mRCC [394].

In summary, these studies suggest that the development of combination therapies involving ICIs and anti-angiogenic drugs holds promising potential as a novel approach, enhancing the anti-tumor immune response in cancer patients. In the future, further analysis of its efficacy and safety should be conducted, with the goal of translating these findings into clinical practice to improve the immunotherapy outcomes of cancer patients.

### PD-1/PD-L1 inhibitors combined with EGFR-TKIs therapy

The Epidermal Growth Factor Receptor (EGFR) on the cell surface is phosphorylated to form a dimer after binding to its ligand, activating downstream pathways like RAS/RAF/MEK/MAPK and PI3K/AKT, subsequently causing tumor cell proliferation, invasion, and apoptosis [395]. EGFR tyrosine kinase inhibitors (EGFR-TKIs) are effective targeted drugs for NSCLC patients with EGFR mutations, acting as analogs of adenosine triphosphate (ATP) that competitively bind to the intracellular EGFR tyrosine kinase site, thereby blocking the activation of downstream pathways and inhibiting tumor growth. However, TKI-targeted therapy also promotes the occurrence of resistance in patients, primarily related to EGFR gene amplification or secondary mutations, and may even lead to hyper-progressive disease [396].

Recent research has shown that in NSCLC with EGFR mutations, EGFR-TKIs regulate PD-1 and PD-L1 expression by inhibiting downstream signaling. In NSCLC patients, activated EGFR signaling upregulates PD-L1 expression by phosphorylating ERK1/2 and c-Jun. Simultaneously, PD-L1 induces the apoptosis of T lymphocytes in the TME through the PD-L1/PD-1 axis, thereby mediating tumor immune evasion. If using EGFR-TKI to inhibit EGFR, it can suppress PD-L1 expression and reactivate anti-tumor immunity in T cells [397]. In a mouse model of EGFR-mutated lung adenocarcinoma, which has a non-inflammatory immunosuppressive tumor microenvironment, EGFR signaling recruits CD4^+^ regulatory T cells and CD8^+^ T cell infiltration by activating JNK/cJun and inhibiting IRF1. Treatment with erlotinib (EGFR-TKIs) in combination with anti-PD-1 mAb has better antitumor efficacy than monotherapy [398]. However, existing clinical research indicates that NSCLC patients with EGFR mutations have a poor response to EGFR-TKIs combined with PD-1/PD-L1 inhibitor immunotherapy, the toxicity reaction significantly increases. For instance, in a phase I clinical trial of 21 advanced EGFR-mutated NSCLC patients, the ORR for Nivolumab combined with Erlotinib (the first generation of EGFR-TKIs) is 15%, and the 24-week PFS rate is 48%. Although this combined therapy shows some preliminary efficacy, the incidence of AEs is also higher [399]. Similarly, in a phase Ib clinical trial of Osimertinib (a third-generation EGFR TKI) combined with the PD-L1 monoclonal antibody durvalumab for the therapy of NSCLC patients with EGFR mutations, the objective remission rate in the Osimertinib monotherapy group is 80%, but only 64% in the combination therapy group. The incidence of interstitial lung disease increases in patients receiving combination therapy, leading to the early termination of the clinical trial [400]. In addition, Su et al. find that EGFR-TKI primary-resistant patients have higher PD-L1 expression compared to those with acquired resistance, resulting in a significant reduction in the objective remission rate and shorting the progression-free survival [401]. This indicates that EGFR-mutated NSCLC patients may have a limited response to EGFR-TKI treatment. Therefore, the safety of the combination therapy of PD-1/PD-L1 inhibitors and EGFR-TKI therapy in NSCLC patients remains unclear, possibly related to PD-L1 expression, tumor mutation burden (TMB), and potential differences in different tumors. Hence, a cautious approach is required when selecting treatment options.

At present, the combination therapy of TKIs and immune checkpoint inhibitors is being studied in various clinical trials, but the results of these trials are not yet mature. Future research should focus on exploring the mechanisms of combination therapy, optimizing drug sequence and dosage, enhancing the research on overcoming TKI treatment resistance, and minimizing the adverse reactions related to tumor immunotherapy, aiming to maximize the benefits of TKIs combined with ICI in clinical treatment.

### PD-1/PD-L1 inhibitors combined with PARP kinase inhibitors

PARPs are a family of proteins involved in DNA damage repair, maintenance of genomic stability, and promotion of apoptosis in tumor cells. Inhibition of PARP may result in the accumulation of DNA damage and double-stranded DNA breaks, thereby increasing tumor mutation and neoantigen load. PARP plays an important role in tumor immunity, DNA damage caused by PARP inhibitors (PARPi) upregulates PD-L1 expression through the cGAS/STING pathway, inhibiting the functions of effector T cells. Alternatively, it promotes inflammatory responses by altering the extrinsic immunogenicity of the tumor. When combined with immune checkpoint inhibitors, PARPi regulates T cell activity in the immune microenvironment, thus activating anti-tumor immune responses [402].

Recently, researchers discovered that PARPi acts as immune modulators in tumor therapy, enhancing the efficacy of immune checkpoint inhibitors. In BRCA1 gene-deficient breast cancer, PARPi also upregulates PD-L1 expression on tumor cell surfaces by inactivating GSK3β, thereby inhibiting the activation of tumor-infiltrating T lymphocytes. Combining PARPi and PD-L1 inhibitors can reactivate anti-tumor immunity [283]. In BRCA1 gene-deficient ovarian cancer, PARPi upregulates PD-L1 expression by promoting the phosphorylation of CHK1 (a cell cycle checkpoint kinase), which is associated with reduced infiltration of CTL. The use of PD-L1 blockers reverses the inhibitory efficacy of PARPi on CD8 T cells, thereby enhancing the antitumor effects of PARPi [403], which also provides a theoretical basis for the combination therapy of PARPi and PD-L1 antibodies in ovarian cancer. Since the accumulation of DNA damage caused by PARPi, there has been an enhancement of the antitumor activity of the PD-L1 antibody by increased expression of new antigens and immune recognition of the tumor. Therefore, it is not difficult to find that cancer patients carrying BRCA gene mutations have specific DNA repair deficiencies and are more sensitive to PARP inhibitors that impede DNA repair. When combined with ICI inhibitors, the anti-tumor effect is better. More signaling regulatory mechanisms need to be further studied.

In the TOPACIO/KEYNOTE-162 trial of combined PARPi and ICI, researchers analyzed the efficacy of Niraparib combined with Pembrolizumab in recurrent ovarian cancer and metastatic TNBC [404]. The results show that in 60 patients with stage 1 and stage 2 ovarian cancer, the ORR is 18% and the DCR is 65%. Among them, 3 cases achieve a complete remission, 8 cases have a partial remission, and 28 cases are stable. Subsequent MEDIOLA clinical trial analysis shows that PARP inhibitor Olaparib combined with PD-L1 inhibitor durvalumab has good efficacy in metastatic breast cancer with BRCA1/2 mutations, with a response rate of 52% and a disease control rate of 80% [405]. Therefore, this combined therapy has shown promising anti-tumor activity in tumor therapy and also lays the foundation for further large-sample randomized controlled trials. Currently, the clinical application of PARPi in combination with ICIs for tumor treatment is still in its infancy, and the development of PARP inhibitors is becoming a hotspot in the field of tumor immunity.

### PD-1/PD-L1 inhibitors combined with targeted receptor agonists

IL-2, a type I cytokine, is the earliest approved for cancer therapy because of its significant and long-lasting efficacy, but the high toxicity of IL-2 at high-dose administration limits its use. NKTR-214 is a selective agonist targeting the IL-2R dimer CD122, it exhibits favorable pharmacological effects with relatively low toxicity [406]. Therefore, it has been used in combination with ICIs in recent years. Studies have shown that after patients receive NKTR-214 treatment, the number of CD8^+^ T cells and NK cells in tumors increases linearly, and the expression of PD-1 and PD-L1 on the cell surface increases [407], suggesting a potential synergistic mechanism between NKTR-214 and PD-1 inhibitors. Currently, results from phase I/II clinical trials combining nKTR-214 with nivolumab have shown a total ORR of 59.5% in 38 patients with specific immunotherapy-naive advanced solid tumors, including 7 complete remissions (18.9%), with good tolerability [408]. Therefore, targeting IL-2 receptor agonists combined with ICI therapy serves as a dual immunotherapy approach for a range of advanced solid tumors.

Toll-like receptors (TLRs) are pattern recognition receptors of the innate immune system, recently been considered key factors in the pathogenesis of tumors. TLR signaling not only affects immune cell activation, maturation and function, but also influences tumor metabolism and metastasis [409]. TLR agonists have been proven to enhance the efficacy of ICIs by increasing the proportion of tumor-specific cytotoxic T cells in preclinical models. For example, in a phase Ib clinical trial for advanced melanoma patients who are unresponsive or have failed PD-1 monotherapy, the TLR9 agonist Vidutolimod induces and attracts anti-tumor T cells by triggering a strong IFN response, thereby reversing PD-1 blockade resistance. When Vidutolimod is combined with pembrolizumab, durable responses and tumor regression are observed in 25% of patients, with controllable safety [410]. Other TLR agonists have also carried out phase I clinical trials combined with PD-1/PD-L1 inhibitors, but specific clinical data have not yet been reported.

Besides the targeted TLR and IL-2 receptor agonist therapies mentioned above, there are ongoing developments in new combinations and targets to enhance the efficacy of ICIs in patients. For example, inhibitors of the adenosine pathway, anti-HER2 agents in gastric cancer, BTK inhibitors, histone deacetylase (HDAC) inhibitors, and demethylating agents are being explored. These targeted therapeutic approaches provide a new way for ICI treatment, but clinical application still needs further study.

### PD-1/PD-L1 inhibitors combined with radiotherapy and chemotherapy

Radiation therapy directly interferes with a patient’s primary tumor, transforming it into situ vaccine. This process recruits more T lymphocytes to infiltrate the tumor lesion and releases immunogenic factors (such as TNF, IL-1, HMGB1, etc.) to indirectly stimulate the immune system, exerting an immunological killing effect [411]. Besides the tumor lesions irradiated by radiation therapy, distant non-irradiated metastatic lesions also shrink, this radiation-induced abscopal effect is likely related to the activation of systemic immune responses. It appears with a relatively higher probability in radiation therapy combined with immunotherapy, significantly increasing patients’ survival [412]. For instance, Yan Lan et al. find that a bifunctional fusion protein, bintrafusp alfa (BA), that simultaneously inhibits TGF-β and PD-L1, effectively synergizes with radiation therapy. The combination of BA^+^ and radiotherapy (BART) increases tumor-infiltrating leukocytes, overcoming tumor immune escape. It also reduces radiation-induced pulmonary fibrosis, ultimately eradicating cancer lesions while preserving normal tissues [413]. In malignant pleural effusion models, radiation tumor cell-released microparticles (RT-MPs) can precisely target M2-TAMs in the TME and convert them into M1-TAMs by activating the JAK-STAT and MAPK pathways, facilitating an anti-tumor immune response. The internalization of RT-MP also significantly enhances PD-L1 expression in TAMs, RT-MP combined with anti-PD-1 therapy shows good biocompatibility and elicits memory immune responses [414]. Combining nanoparticle-mediated immune radiotherapy with dual blockade of LAG3 and TIGIT can enhance the treatment effect of anti-PD-1 resistant lung cancer [415].

In clinical research, radiation therapy has demonstrated synergistic effects with various immunotherapies. Combining radiation therapy with PD-1/PD-L1 blockade therapy can activate tumor-specific T cells in the TME and reduce the accumulation of MDSCs and Tregs, thereby enhancing anti-tumor immunity [416]. A phase II trial reports that radiation therapy improves the response of MSS CRC and PDAC patients to ipilimumab and nivolumab immunotherapy [417]. Theelen *et al.’*s retrospective analysis found that patients with metastatic NSCLC benefit from the abscopal effect of radiotherapy in combination with Pembrolizumab immunotherapy, with a favorable clinical prognosis. The combination of PD-1 monoclonal antibody and radiation therapy (iRT) shows a significantly higher abscopal response rate (ARR) than the PD-1 monoclonal antibody treatment group (41.7% vs. 19.7%). For non-irradiated lesions, radiation therapy at least doubles the efficacy of PD-1 monoclonal antibody, significantly improving the treatment outcome and extending the patients’ survival [418]. These studies have also propelled the conduct of a randomized open-phase III trial of Nivolumab combined with radiation therapy for treating glioblastoma [419].

Chemotherapy mainly kills cancer cells by targeting their DNA synthesis and replication processes. Preclinical studies have shown that chemotherapy promotes the infiltration of T cells into tumor tissues and decreases the number of immunosuppressive cells, thus enhancing the sensitivity of tumor cells to immune checkpoint blockade therapy [420]. Therefore, the appropriate combination of chemotherapy drugs with PD-1/PD-L1 inhibitors enhances the effects of anti-PD-1/PD-L1 therapy and significantly improves overall survival in patients. For example, In clinical randomized trials, the combination of Pembrolizumab with chemotherapy can significantly improve the OS for patients with metastatic triple-negative breast cancer [421] and metastatic squamous NSCLC [422], and also prolong PFS. Similarly, in patients with advanced NSCLC and PD-L1 expression ≥ 50%, the combination of first-line cemiplimab (a PD-1 inhibitor) and chemotherapy significantly improves OS and PFS [423]. These findings indicate that adding PD-1/PD-L1 inhibitors to standard chemotherapy is important in the first-line treatment of various solid tumors. However, chemotherapy drugs not only kill tumor cells but often have serious side effects. Common side effects include bone marrow suppression, which leads to decreased white blood cells, red blood cells, and platelets. Severe bone marrow suppression results in extremely low immunity and coagulation disorders, early symptoms may not be apparent. If not promptly detected and corrected in time, it can lead to severe infection and vital organ bleeding, and other fatal complications [424]. Therefore, when choosing chemotherapy, patients need an experienced physician to develop reasonable plans, closely observe adverse reactions during chemotherapy, timely monitor routine blood tests and pay attention to other symptoms related to chemotherapy. If discomfort is found after chemotherapy, communicate with the attending physician in time and take relevant treatment measures.

In summary, more and more clinical trials suggest that when combining PD-1/PD-L1 inhibitors with different radiotherapy/chemotherapy protocols, the impact of different dosage regimens in terms of efficacy and prognosis is quite different. Therefore, the exploration of larger sample sizes is required to extensively investigate the underlying mechanisms behind the differential treatment outcomes among different groups, and to validate the feasibility of combined therapeutic approaches involving chemotherapy and PD-1 antibodies.

### Photodynamic therapy (PDT) combined with PD-1/PD-L1 inhibitors for anti-tumor therapy

Photodynamic therapy (PDT) is a novel adjuvant tumor therapy based on photosensitizers, oxygen molecules and light sources. When the photosensitizer is absorbed and metabolized by the target tissue, cell-toxic singlet oxygen (^1^O2) and reactive oxygen species (ROS) are generated through a series of photochemical reactions under the stimulation of an appropriate light source in the presence of oxygen. These reactive species kill the tumor tissue either by directly causing cell death through triggering signal cascade reactions, thereby achieving therapeutic purposes [425].

In recent years, researchers have combined immune checkpoint inhibitors (ICI) with PDT for adjuvant anti-tumor therapy, achieving preliminary success in animal models of breast cancer, colon cancer, renal cancer, lung cancer, and skin cancer [426]. For example, small-molecule nano-inhibitor targeting the PD-1/PD-L1 axis (BMS202) mediates ICI and PDT synergistically in the therapy of breast cancer, leading to primary tumor regression and reducing the occurrence of lung metastasis [427]. Hwang et al. use a combination of PDT and flagellin-assisted tumor-specific peptide vaccine (FlaB-Vax) in treating B16-F10 melanoma mouse models that had undergone PD-1 blockade therapy. This combination effectively induces systemic anti-tumor immune responses, elevating CD8^+^ T cell infiltration and systemic IFN-γ secretion, enhancing the accumulation of effector memory CD8^+^ T lymphocytes, further strengthening the efficacy of PD-1 blockade therapy [428]. Yuan et al. find that the multifunctional mTHPC@VeC/T-RGD NPs platform (a multifunctional nanoparticle loaded with photosensitive mTHPC) mediated PDT treatment enhances the anti-tumor efficacy of PD-L1 inhibitors in colorectal cancer therapy. Under 660 nm near-infrared (NIR) laser irradiation, mTHPC@VeC/T-RGD NPs can kill tumor cells by inducing apoptosis and stimulating a systemic immune response, while PD-L1 blockade further suppresses tumor growth, metastasis, and forms long-term host immune memory to prevent tumor recurrence [429]. Peng Huang’s team developed a biomimetic nanoemulsion expressing PD-1 on its cell membrane, which is used for synergetic photodynamic immunotherapy against hypoxic breast cancer, the treatments inhibit tumor growth by promoting dendritic cell maturation and tumor infiltration by cytotoxic T lymphocytes. Additionally, the co-delivering photosensitizer with PD-1 protein using this nanoemulsion achieved synergistic effects of PDT and immunotherapy [430].

Therefore, PDT possesses advantages such as precise targeting, repeatable treatment, and low toxic side effects, making it a good clinical application prospect for combination therapy with ICI. However, due to the limited penetration depth of traditional PDT light sources, it is more suitable for treating superficial tumors and less effective for deep-seated tumors. At present, there are few clinical studies combining ICI and PDT, with only a few case reports and one ongoing clinical trial. In the future, how to further improve the selectivity and specificity of photosensitizers to target tumor therapy, enhance the penetration depth of PDT, and develop new technologies and methods that combine nanophotonic technology with biomedical applications for diagnosis and treatment will be the new directions for PDT development. With the advancement of technology, PDT technology will make greater contributions to human health.

### Aptamer drug conjugates (ApDC) combined with PD-1/PD-L1 inhibitor for anti-tumor therapy

Aptamer drug conjugates(ApDC)are highly promising novel immunotherapies. Studies have shown that ApDC provides immune modulators to restrict immune co-stimulation to the tumor area and also induces new antigens within the tumor, thereby blocking immune checkpoints and activating functional immune cells, prolonging anti-tumor immunity [431]. With the development of cancer immunotherapy, therapeutic aptamers have been reported for targeting immune checkpoints, including CTLA-4, PD-1, and PD-L1. For example, self-assembling nanoparticles of multivalent aptamer-drug conjugates (ApMDC) enhance the anti-tumor responses of anti-PD-1 immunotherapy [432]. Engineered multifunctional aptamers (P1/C4-bi-apt) improve anti-tumor immune responses by blocking the dual immune checkpoints of CTLA-4 and PD-L1 [433]. Additionally, DNA aptamers targeting PD-1/PD-L1 have been developed to disrupt the immunosuppressive interaction between the immune-inhibitory PD-1 and PD-L1 molecules. Prodeus et al. discover that MP7 (a short synthetic anti-PD-1 DNA aptamer) specifically binds to the extracellular domain of PD-1, competitively antagonizes the direct binding between PD-1 and PD-L1 molecules, functionally blocking PD-L1-mediated immune suppression signals and ultimately inhibiting the growth of colon cancer [434]. LAI et al. discover that aptPD-L1 (a PD-L1 antagonistic DNA aptamer) specifically binds to PD-L1, thereby blocking the interaction between PD-1 and PD-L1. In vitro experiments show that aptPD-L1 promotes lymphocyte proliferation and inhibits tumor growth in vivo, it helps in restoring T cell function and altering the TME without causing hepatorenal toxicity [435]. Li Wenzhe et al. develop a metal-organic framework nanoparticle (M@O-A) based on the PD-L1 nucleic acid aptamer (aptPD-L1) to achieve efficient and low-toxic tumor photo-immunotherapy [436]. It first uses aptPD-L1 to block the immune suppressive function of PD-L1 protein on the surface of tumor cells, then under near-infrared light irradiation, the nanosystem can perform PDT simultaneously, thus inducing immunogenic cell death (ICD) in tumor cells, synergistically activating the immune system with potent tumor cell killing capability and lower immune-related toxicity.

These aptamers, due to their similar tumor inhibitory efficacy to antibodies and easier synthesis compared to antibodies, have advantages such as low immunogenicity, low risk of inducing autoimmune toxicity, high targeting specificity, and low toxicity. Therefore, they serve as alternative therapeutic agents for cancer immunotherapy. Even though nucleic acid aptamers have made numerous advances in the field of tumor immunotherapy research, applying them to clinical drug treatments still faces many challenges. For example, the screening process of nucleic acid aptamers is often long and nucleic acid aptamers usually require appropriate chemical modification to exert their anti-tumor effects. Further preclinical studies are needed to address issues such as the stability of nucleic acid aptamers in the bloodstream, the targeting efficiency of drug delivery systems, and the metabolism and clearance pathways of drugs in the body.

### PD-1/PD-L1 inhibitors combined with immune cell therapy

Adoptive cell therapy (ACT) involves the infusion of T cells derived from TILs, or T cells that have been genetically engineered to express tumor-recognizing receptors into the body, allowing them to directly exert anti-tumor effects and promote the host’s anti-tumor immune responses [437]. Preclinical studies on ACT using genetically engineered T cell receptors (TCRs) and chimeric antigen receptors (CARs) have achieved promising results, some T cell therapies have received regulatory approval for the therapy of patients with B cell malignancies.

Research has found that combining PD-1/PD-L1 blockade antibodies with ACT increases the speed of tumor therapy responses. For example, in NSCLC, combining anti-PD-1/PD-L1 with CAR-T cell therapy promotes the restoration of normal immune recognition function and maintains immune system homeostasis [438]. Phase 1 clinical trial results of TIL adoptive cell therapy combined with Nivolumab (NCT03215810) show that among 13 patients, 3 confirmed responses, 11 have reduced tumor burden, including 2 who achieved complete remission after 1.5 years, effectively preventing subsequent relapse associated with PD-L1 expression [439]. Therefore, TIL mediates an effective response in tumors that are insensitive to traditional ICIs. With the advancement of ACT cell immunotherapy technology, researchers are considering whether, in addition to using PD-1 or PD-L1 inhibitors, CAR-T cells secreting PD-1 antibodies can be used to treat recurrent solid tumors. Fang et al. first report that patients with advanced refractory ovarian cancer who received autologous PD-1-meso CAR-T cells (secreting full-length PD-1 antibodies) treatment have expanded CAR-T cell numbers in the bloodstream, and their own CD8^+^ T cell activity is also significantly enhanced, indicating that CAR-T cells infused back into the human body can mobilize immune cells to target tumor cells efficiently and precisely, rapidly improving the tumor local microenvironment’s immune function to kill cancer cells without causing significant adverse reactions [440]. In addition, Lu’s team conducted the first phase I clinical trial to inject CRISPR/Cas9 edited PD-1 knockout T cells into patients with advanced NSCLC. The results show that edited T cells are detected in peripheral blood, with a median PFS of 7.7 weeks and a median OS of 42.6 weeks, with 2 patients experiencing stable disease. This research demonstrates that the clinical application of CRISPR/Cas9 knocked-out PD-1 T cells is safe and feasible, but lacks significant efficacy in NSCLC patients [441]. Similarly, the Stadtmauer team uses CRISPR technology to genetically modify T cells in 3 patients with advanced cancer, they delete three genes, TCRα, TCRβ, and PD-1, to enhance the functions of T cells. They also introduce a synthetic cancer-specific TCR transgenic (NY-ESO-1) to recognize tumor cells, and finally the multiple-engineered T cells are reinfused into the patients. Studies show that all three patients tolerated the treatment well, these genetically edited T cells survive for up to 9 months in the patients, exhibiting the ability to consistently attack and kill tumors with few treatment-related serious adverse reactions [442]. Therefore, cell therapy that uses CRISPR-Cas9 technology to specifically knockout PD-1 in CART-T cells or T cells to improve the anti-tumor activity of effector T cells and overcome drug resistance in advanced metastatic tumors has been clinically proven to be safe and effective. In the future, new strategies should be developed to avoid the off-target risk of CRISPR/Cas9 gene editing. Meanwhile, the CRISPR/Cas9 system, as an emerging technology in cell therapy, is expected to achieve greater breakthroughs in combination therapy with PD-1/PD-L1 in the field of tumor immunotherapy.

### PD-1/PD-L1 inhibitors combined with oncolytic virus therapy

Oncolytic virus therapy is a series of immune cascade reactions that directly kill tumor cells by injecting oncolytic viruses (OVs) into tumors and causing systemic anti-tumor immune responses, which promote the antitumor responses of tumor-specific T cells [443]. Currently, various natural and transgenic viruses have been developed as oncolytic agents. OVs improve the therapeutic responses to PD-1 therapy by reversing immune suppressive factors, increasing the number and diversity of infiltrating lymphocytes and promoting PD-L1 expression in tumors [444]. For example, Hamdan et al. find that the oncolytic adenovirus Ad-Cab secretes a cross-hybrid Fc fusion peptide targeting PD-L1, thereby activating the Fc effector mechanisms of IgA1 (neutrophil activation) and IgG1 (natural killer and complement activation). Compared to the conventional PD-L1 inhibitor Atezolizumab, Ad-Cab demonstrates higher anti-tumor activity and activates multiple immune effector cell populations, thereby enhancing tumor killing capacity in vivo or in vitro, and in patient-derived tumor organoids [445]. Kiyokawa et al. find that treating mice with GBM using ICOVIR17 (an oncolytic adenovirus expressing hyaluronidase) increases CD8^+^ T-cell and macrophage infiltration and upregulates PD-L1 expression. Combining ICOVIR17 with PD-1 blockade antibodies induces tumor-associated pro-inflammatory macrophages and tumor-specific T cell cytotoxicity locally and systemically, enhancing tumor killing and further extending the survival of GBM mice [446]. Hence, OVs combined with PD-1/PD-L1 inhibitors may be beneficial to patients who have a poor response to immune checkpoint inhibition.

### PD-1/PD-L1 inhibitors combined with cancer vaccines

Cancer vaccines work by enhancing immune recognition by using genetically modified genes or new antigens, enabling stronger immune responses to kill cancer cells and preventing their growth and recurrence. Compared to chemotherapy and radiotherapy, cancer vaccines usually have minimal side effects [447]. In recent years, there has been extensive research on personalized cancer vaccines for cancer treatment. For example, FixVac (BNT111), a nanoparticulate liposomal RNA (RNA-LPX) vaccine for intravenous administration developed by Ugur Sahin et al., has shown that it induces PD-1^+^ effector memory T cells in Phase I clinical trials for patients with advanced melanoma. Some patients who received FixVac after failing anti-PD-1 therapy exhibit tumor regression. The combination of FixVac and anti-PD-1 treatment is particularly effective for patients with a lower mutation burden, with a tumor regression rate exceeding 35% [448]. Additionally, a clinical phase I/II trial shows that Nivolumab combined with IO102/IO103 (an immune regulatory vaccine targeting IDO and PD-L1) achieves a high ORR of 80% in metastatic melanoma patients, with 43% achieving complete remission, this combination significantly decreases the tumor burden and extends PFS to 26 months [449]. Therefore, tumor vaccination in conjunction with checkpoint inhibition may be an effective immunotherapy regimen.

In summary, combination therapy offers more advantages, can also reduce the toxicity of anti-PD-1/PD-L1 immunotherapy and the possibility of acquired drug resistance. However, the long-term impact of chemotherapy on long-term immune cells is still unknown due to the insufficient number of clinical patient samples. As clinical studies evaluating combined immunotherapies rapidly increase, it is crucial to comprehensively understand the molecular mechanisms of anti-PD-1/PD-L1 therapies and identify more valuable targets to enhance the effectiveness of immunotherapy, broaden the beneficiary population, and further improve immune efficacy.

### PD-1/PD-L1-mediated immune resistance and side effects

In recent years, although immune checkpoint blockade therapy targeting the PD-1/PD-L1 axis has shown significant therapeutic effects in the treatment of multiple malignancies, the response rate of anti-PD-1/PD-L1 mAb in the overall patient population is far from satisfactory. A large portion of patients do not respond, one of the major obstacles to the ICI therapy found in clinical use is that most patients develop resistance and relapse in the later stages of treatment [450]. Due to the high incidence of primary and acquired drug resistance, most patients cannot benefit from these therapies. Moreover, the complex resistance mechanisms of tumor cells to immunotherapy impact the clinical prognosis of patients [451]. Studies have found that the drug resistance to the PD-1/PD-L1 monoclonal antibody may be associated with the following factors, the intrinsic immune evasion genetic pathways in tumor cells, tumor antigen mutations and presentation processes, interactions among multiple immune checkpoints, suppressive tumor microenvironment (tumor intrinsic factors, inflammatory environment), oncogenic pathways in tumors, epigenetic changes in key tumor proteins, accumulation of metabolites, etc [452]. Therefore, it is urgent to understand the mechanisms of resistance to PD-1/PD-L1 inhibitors in immunotherapy, to discover universal biomarkers across multiple tumors, to develop combination therapies to sensitize resistant patients, and to find new strategies to enhance the efficacy of ICIs.

Among various types of cancer, although PD-1/PD-L1 blockade has clinical benefits, most patients do not experience durable or even curative remission after treatment and eventually relapse. The reasons for insufficient tumor immunotherapy response may be due to the heterogeneity of PD-1/PD-L1 expression levels in different tumor patients, low tumor mutation burden, and low tumor immunogenicity. Although the expression of PD-1/PD-L1 is crucial during ICI treatment, targeting PD-1 with antibodies disrupts PD-1 signaling on all cells, not just tumor-specific T cells, which may induce an imbalance in the immune system and cause collateral damage. Furthermore, studies have detected the same TCR sequences in both tumor and normal tissues [453], suggesting that during ICI treatment, T cells may infiltrate both sites, leading to indiscriminate immune attacks on systemic tissues during ICI treatment, thereby producing immune-related side effects and ultimately leading to treatment failure in cancer patients.

PD-1/PD-L1 immunotherapy is often accompanied by life-threatening immune-related adverse events (irAEs), irAEs induced by immunotherapy are usually insidious in onset, lack specificity, and have a broad toxic spectrum [454]. Physicians need to enhance the management of irAEs in terms of prevention, examination, and treatment to effectively control the diseases. Compared to conventional treatment, the main side effects caused by anti-PD-1/PD-L1 treatment include Atezolizumab causing hyperthyroidism, nausea, etc. Nivolumab causes endocrine toxicity and cardiotoxicity, etc. Pembrolizumab causes arthralgia, fatigue, hepatotoxicity, etc. Ipilimumab causes skin and gastrointestinal toxicity, etc. Tremelimumab causes rash, diarrhea, fatigue, etc [455]. Taking cardiotoxicity and myositis in irAEs as examples, Johnson et al. report fatal myocarditis in two melanoma patients after combination treatment with nivolumab and ipilimumab. Both patients developed myocarditis with rhabdomyolysis, cardiac electrical instability, and massive infiltration of T cells and macrophages. The severity of myocarditis is also more severe than monotherapy [456]. Subsequently, Wei et al. identify a preclinical mouse model of ICI-related myocarditis (Ctla4^+/−^Pdcd1^−/−^mice) induced by this combination therapy, and prove that intervention treatment with Abatacept (a recombinant soluble fusion protein of CTLA-4) inhibits T cell co-stimulation and improves disease progression [457]. In terms of early intervention, the latest study by the Joe-Elie SALEM team found that early management measures for ICI myocarditis patients, such as mechanical ventilation, high-dose Abatacept and Ruxolitinib (a JAK1/JAK2 inhibitor) treatment combined with CD86 RO (CD86 receptor occupancy on circulating monocytes) monitoring, hold promise for reducing the high mortality rate caused by ICI-induced myocarditis, but further research is still needed to evaluate the optimal drug combination and dosage [458]. Therefore, further research might be required to investigate the mechanisms of these checkpoint therapies to improve these toxicities, effective and accurate diagnosis and therapeutic approaches should be sought to reduce the severity of complications.

## Conclusion

The clinical application of immune checkpoint inhibitors (ICI) targeting PD-1/PD-L1 has completely transformed traditional cancer treatment. With the successful clinical trials of novel ICB drugs and combination therapies, as well as innovative breakthroughs in mRNA vaccines and nanomaterial delivery system technologies, ICIs show great promise in cancer therapy. Despite the significant effects of PD-1/PD-L1 tumor immunotherapy in prolonging patient survival and improving the prognosis of advanced cancer patients, the development of PD-1/PD-L antibodies in tumor immunity will also face more challenges, such as resistance developed from long-term use of PD-1 or PD-L1 inhibitors, how to improve drug utilization, and how to select the best combination therapy. In addition, PD-1/PD-L1 blockade is limited by the low response rates in certain cancers, the lack of known biomarkers, immune-related toxicity, and inherent and acquired resistance. Therefore, in basic mechanism research, PD-1/PD-L1 expression levels, IFN-γ, CD8^+^ T cell infiltration, and tumor mutational burden (TMB) are the most widely used biomarkers. In the future, we will explore new mechanisms of targeting PD-1 or PD-L1 and new directions in combination therapy, jointly targeting the adaptive stress pathways related to metabolism, oxidation, and DNA damage repair in the tumor, increasing the abundance of tumor immune cells, to provide options for improving response rates and preventing drug resistance. We also attempt to change the local tumor microenvironment, neutralize immune suppressive factors within the TME, block immune suppressive responses, restore the immune system’s homeostasis, and reactivate T cells’ immunity and the patient’s autoimmunity against cancer.

Beyond the conclusions discussed in this review, the regulatory differences in immune systems among species, the selection of preclinical animal models, the conduct of clinical trials, the clinical use of approved ICB drugs, the optimization of combination therapy regimens, and the individualization of immunotherapy based on the specificity of patients and the high heterogeneity of tumors are all important directions to consider. In clinical applications, when different clinical factors (such as age, gender, and obesity) are involved, patients’ responses to drug treatment may slightly vary. There is still a lack of optimal in vitro and in vivo experimental models for assessing the efficacy, immune mechanisms, and drug toxicity of new immunotherapies. Therefore, developing 3D co-culture models of tumor cells and immune cells or patient-derived xenografts (PDXs) and organoid model technologies represents the future of precision treatment, which is important for accelerating the development, screening, clinical translation, and evaluation of targeted therapies for the next generation of ICB drugs. Due to the complex dynamic equilibrium in the immune microenvironment and the heterogeneity of tumors in each patient, personalized immunotherapies need to be investigated in the future. The emergence of single-cell multi-omics studies and innovative high-throughput sequencing technologies opens new avenues towards personalized patient treatment. For example, in heterogeneous diseases such as bladder cancer, multi-omics sequencing and analysis may help provide stronger biomarkers for predicting chemosensitivity [459]. Gene expression models established based on multi-omics sequencing data can be used to screen specific patient groups that may respond well to cytotoxic drugs, achieving precise targeted therapy. We believe that in the future, the latest advancements in artificial intelligence-based algorithms and models will soon be able to predict responses to immunotherapy and combination therapy at a personalized level. Undeniably, since PD-1/PD-L1 inhibitors are still expensive drugs in many countries, adopting combination therapy regimens will impose a significant financial burden on patients. Hence, developing effective predictive biomarkers to select patients who may benefit from ICI treatment, optimizing combination therapy regimens (including drug dosages and usage sequences, etc.), optimizing pharmaceutical processes, reducing drug prices, and preventing patients from incurring huge healthcare costs due to over-treatment, is a key issue for improving people’s livelihoods.

In summary, the regulation of the PD-1/PD-L1 pathway in tumor immunity is complex and fascinating. A comprehensive understanding of the metabolic mechanisms behind immune escape, the accurate and reliable identification of predictive biomarkers, and therapeutic targets for the response of different cancers to immune checkpoint inhibitors are necessary to improve immunotherapy for tumors. Notably, during PD-1/PD-L1 therapy, the immune system may be in an over-activated state, damaging organ systems and even causing immune-related adverse events such as myocarditis, making it imperative to comprehensively monitor the immune system and prevent systemic diseases in a timely manner. It is believed that with continued research, the role and resistance mechanisms of the PD-1/PD-L1 pathway in the regulation of the body’s immunity and tumor immunotherapy will be thoroughly elucidated. This will provide new strategies for combined immunotherapy in the clinical treatment of solid tumors, increase the personalization of cancer treatment, and benefit more patients.

## Abbreviations

APC: Antigen-presenting cells
ACT: Adoptive cell therapy
ApDC: Aptamer drug conjugates
ALK: Anaplastic Lymphoma Kinase
BRD4: Bromodomain-containing protein 4
BET: Bromodomain and extra-terminal domain
CTC: Circulating tumor cell
CLL: Chronic lymphocytic leukemia
CBR: Clinical benefit response
CNV: Copy number variation
ceRNAs: Competitive endogenous RNAs
circRNAs: Circular RNAs
CMTM6: CKLF-like MARVEL transmembrane domain containing 6
CSCC: Cutaneous squamous cell carcinoma
CDK: Cyclin-dependent kinase
CAR: Chimeric antigen receptor
DLBCL: Diffuse large B-cell lymphoma
DC: Dendritic cell
DHHC3: Aspartate-histidine-histidine-cysteine 3
DHHC9: Aspartate-histidine-histidine-cysteine 9
DCR: Disease control rate
EAE: Experimental autoimmune encephalomyelitis
EMT: Epithelial-mesenchymal transition
EDMC: Erythroid-Derived Myeloid Cells
EGF: Epidermal growth factor
ER: Endoplasmic reticulum
ERAD: Endoplasmic reticulum associated degradation
FAO: Fatty acid oxidation
FTO: Obesity-associated protein
GVHR: Graft-versus-host reaction
GADA: Glutamic acid decarboxylase antibody
GC: Gastric cancer
GSDMC: Gasdermin C
GSK3β: Glycogen synthase kinase 3β
HLA/APM: Antigen-presenting machinery elements
HVGR: Host versus graft reaction
HCC: Hepatocellular carcinoma
HPD: Hyperprogressive disease
HIF-1α: Hypoxia-inducible factor-1α
HDAC3: Histone deacetylase 3
ITIM: Immunoreceptor tyrosine-based inhibitory motif
ITSM: Immunoreceptor tyrosine-based switch motif
ICIs: Immune checkpoint inhibitors
IgG: Immunoglobulin G
irAEs: Immune-related adverse events
ICT: Immune checkpoint therapy
JAK2: Janus kinase 2
LTi: Lymphoid tissue inducer cells
lncRNAs: Long non-coding RNAs
LSD1: Lysine-specific Demethylase 1
MHC: Major histocompatibility complex
MS: Multiple sclerosis
mAbs: Monoclonal antibodies
miRNAs: MicroRNAs
m^6^A: N6-methyladenosine
mRCC: Metastatic renal cell carcinoma
NOD: Non-obese diabetic
NK: Natural killer cell
NAMPT: Nicotinamide phosphoribosyltransferase
NAD^+^: Nicotinamide adenine dinucleotide
NSCLC: Non-small cell lung cancer
OS: Overall survival
ORR: Objective response rate
OVs: Oncolytic viruses
PD-1: Programmed cell death protein 1
PD-Ls: Programmed cell death ligands
PFS: Progression-free survival
PDT: Photodynamic therapy
RA: Rheumatoid arthritis
SHP2: Src homology region 2 domain-containing phosphatase-2
SLE: Systemic lupus erythematosus
sPD-1: Soluble PD-1
STS: Soft tissue sarcoma
SR: Structural rearrangement
SPOP: Speckle type BTB/POZ protein
STUB1: STIP1 homology and U-box containing protein 1
Tregs: Regulatory T cells
TCR: T-cell receptor
T1DM: Type 1 diabetes mellitus
TILs: Tumor-infiltrating lymphocytes
Tex cells: T cell exhaustion
TME: Tumor microenvironment
TdLN: Tumor-draining lymph node
TAM: Tumor-associated macrophage
TIC: Tumor-initiating cell
TNBC: Triple-negative breast cancer
TKI: Tyrosine kinase inhibitor
TLR: Toll-like receptor
VEGF: Vascular endothelial growth factor
3'-UTR: 3’ untranslated region
